# Multifocal Noninvasive Deep Brain Stimulation to Enhance Cognition in Mild Cognitive Impairment: A Crossover Trial

**DOI:** 10.1001/jamanetworkopen.2026.21756

**Published:** 2026-07-06

**Authors:** Umberto Nencha, Monika Pupíková, Margaux di Natale, Pablo Maceira-Elvira, Martin Gajdoš, Elena Beanato, Stavriani Skarvelaki, Rebecca Jones, Isabel Ericson, Martin Lamoš, Andrea Nuti, Fabienne Windel, David Ondráček, Adam Šimo, Ela Vojtková, Klára Špunarová, Vincent Alvarez, Oana Simionescu, Giovanni B. Frisoni, Estelle Raffin, Irena Rektorová, Friedhelm C. Hummel

**Affiliations:** 1 Defitech Chair of Clinical Neuroengineering, Neuro-X Institute, École Polytechnique Fédérale de Lausanne, Geneva, Switzerland; 2 Defitech Chair of Clinical Neuroengineering, INX, EPFL Valais, Clinique Romande de Réadaptation, Sion, Switzerland; 3 Geneva Memory Center, Department of Rehabilitation and Geriatrics, Geneva University Hospitals, Geneva, Switzerland; 4 Neuroscience Program, Central European Institute of Technology, Masaryk University, Brno, Czech Republic; 5 Wyss Center for Bio- and Neuroengineering, Geneva, Switzerland; 6 Faculty of Medicine, Masaryk University, Brno, Czechia; 7 LPNC, CNRS UMR 5105, Université Grenoble Alpes, Saint-Martin-d’Heres, France; 8 Department of Neurology, Hôpital du Valais, Sion, Switzerland; 9 First Department of Neurology, St Anne University Hospital, Faculty of Medicine, Masaryk University, Brno, Czech Republic; 10 Clinical Neuroscience, Geneva University Hospitals, Geneva, Switzerland

## Abstract

**Importance:**

Impairment of working memory (WM), which relies on distributed cortical and subcortical structures, including cerebello-striatal pathways, is a core contributor to functional decline in patients with mild cognitive impairment (MCI) and dementia. Because the striatum is affected early in dementia, whereas the cerebellum remains relatively preserved, the cerebellum may offer support for maintaining striatal function in neurodegenerative diseases.

**Objective:**

To evaluate whether sequential cerebellar transcranial magnetic stimulation (TMS) and striatal transcranial temporal interference stimulation (tTIS) enhances WM in patients with MCI, especially those with early striatal degeneration, like MCI with Lewy bodies (MCI-LB).

**Design, Setting, and Participants:**

This randomized, quadruple-blind, placebo-controlled, crossover trial was conducted from August 2023 to October 2024 at 1 site in Switzerland and 1 in Czech Republic. Participants included individuals with MCI-LB or amnestic MCI (aMCI) and age-matched healthy controls (HCs).

**Intervention:**

Three pseudorandomized single stimulation sessions: (1) cerebellar TMS followed by striatal tTIS, (2) control TMS followed by striatal tTIS, and (3) an active control combining control TMS with control striatal tTIS.

**Main Outcomes and Measures:**

The primary outcomes were changes in accuracy and correct answer reaction times in a visual WM task with varying memory loads and distractors. Modulators included resting-state functional connectivity and gray-matter volume in WM-related regions. Linear mixed-effects models were used to examine the main effects of stimulation condition on accuracy and reaction times.

**Results:**

Forty-one patients with MCI, including 21 with MCI-LB (16 female [76%]; mean [SD] age, 71.05 [6.84] years) and 20 with aMCI (11 female [55%]; mean [SD] age, 72.30 [7.50] years), and 20 HCs (10 female [50%]; mean [SD] age, 69.5 [4.42] years) were analyzed. In patients with MCI-LB, cerebellar TMS plus striatal tTIS increased accuracy in distractor trials compared with the active control (control TMS plus control tTIS, *P* < .001; *d* = −0.77) and striatal tTIS alone (control TMS plus striatal tTIS, *P* = .004; *d* = −0.60). Greater accuracy gains were associated with lower putaminal connectivity (*r* = −0.66; *P* = .03). In patients with aMCI, cerebellar volumes showed associations with task performance in both distractor trials (cerebellar TMS plus striatal tTIS, *R*^2^ = 0.27; *P* = .02) and high-load trials (control TMS plus striatal tTIS, *R*^2^ = 0.24; *P* = .03), and larger volumes were associated with faster responses; however, significant behavioral differences between active and control stimulation conditions were not observed. No serious adverse events occurred.

**Conclusions and Relevance:**

Synergistic dual-target cerebellar TMS and striatal tTIS improved WM in MCI-LB, particularly in patients with lower striatal connectivity, suggesting dual-targeted neuromodulation as a potential therapeutic avenue in neurodegenerative disorders.

**Trial Registration:**

ClinicalTrials.gov Identifier: NCT07090681

## Key Points

**Question:**

Does synergistic, dual-target, noninvasive neuromodulation of the striatum and cerebellum improve working memory (WM) in individuals with mild cognitive impairment (MCI), particularly those with early striatal degeneration?

**Findings:**

In this randomized, quadruple-blind, placebo-controlled crossover trial of 41 patients with MCI, dual-target stimulation significantly improved WM accuracy in individuals with early striatal degeneration. Stimulation-related gains were associated with striatal functional connectivity and cerebellar volume.

**Meaning:**

These findings suggest that targeted engagement of cerebello-striatal circuits through coordinated dual-target noninvasive neuromodulation may represent a promising strategy to enhance WM in early stages of a neurodegenerative disease.

## Teaser

This randomized crossover trial examines whether sequential cerebellar transcranial magnetic stimulation and striatal transcranial temporal interference stimulation enhance working memory in individuals with mild cognitive impairment (MCI).

## Introduction

Executive functions, and particularly working memory (WM), are essential in daily life, enabling individuals to select relevant information, plan complex actions, or adapt behavior in real time.^1^ WM is frequently impaired in patients with mild cognitive impairment (MCI), a condition that often precedes dementia, and is one of the main factors contributing to loss of daily functioning and autonomy. Therefore, we asked whether synergistic, dual-target, noninvasive neuromodulation of the striatum and cerebellum improves WM in individuals with MCI, particularly in those with early nigro-striatal degeneration.

WM is affected via distinct mechanisms in neurodegenerative diseases, such as Alzheimer disease or dementia with Lewy bodies (DLB), with deficits in the latter appearing earlier and being more pronounced.^2^ In Parkinson disease, deficits in visual WM have been related to abnormal distraction-dependent striatal connectivity, suggesting that a similar vulnerability may also contribute to early WM deficits in DLB.^3^ The cerebellum, on the other hand, has been associated with cognitive resilience,^4^ with the left hemisphere being more implicated in visual WM,^5^ and it has been shown to interact with the striatum during the temporal processing of complex tasks.^6^ Although both the striatum and the cerebellum are affected in these disorders, the former is impacted more severely and at earlier stages in DLB than in Alzheimer disease.^7^ In this context, early interventions targeting the striatum and the cerebellum could represent a promising strategy for cognitive rehabilitation and enhanced resilience against degeneration.

In mice, cerebellar output modulates striatal activity via di-synaptic dentato-striatal projections, targeting dopaminergic and cholinergic neurons.^8^ Extensive striato-cerebellar connections also exist in humans^9^ and support both motor and cognitive functions.^10^ Thus, leveraging the connections between these 2 hubs may offer a novel, transformative approach to enhance WM, particularly in patients with MCI. Although noninvasive stimulation of the striatum for modulating WM remains largely unexplored, cerebellar transcranial magnetic stimulation (TMS) can modulate WM performance, particularly when applied to the left inferior cerebellar hemisphere for visual WM.^11^

In this trial, we applied TMS to target the left inferior cerebellar hemisphere and striatal transcranial temporal interference stimulation (tTIS), a novel noninvasive method for focal deep-brain modulation, using 2 plasticity-inducing protocols.^12,13,14,15^ This dual-targeting protocol is unprecedented as an interventional strategy, especially in MCI. We hypothesized that cerebellar TMS followed by striatal tTIS would enhance WM, particularly in patients with MCI with Lewy bodies (MCI-LB), during a visual WM task manipulating distractor inhibition and memory load. We further assessed whether the 2 MCI cohorts would respond differently to various task conditions and whether individual resting-state (rs) functional connectivity and gray matter volume of stimulated regions might influence the behavioral effects of the synergistic dual-target stimulation.

## Methods

### Study Design

In this quadruple-blind, randomized, placebo-controlled, crossover-design trial, we enrolled patients with MCI and age-matched healthy controls (HCs) from 2 research sites (École Polytechnique Fédérale de Lausanne [EPFL], Geneva, Switzerland; and Masaryk University [MUNI], Brno, Czech Republic). Findings were reported using the Consolidated Standards of Reporting Trials (CONSORT) reporting guideline.^16^ All studies were conducted in accordance with the Declaration of Helsinki^17^ and were approved by the Cantonal Ethics Committee of Vaud, Switzerland, and ethics committee of Masaryk University. All participants provided written informed consent before the data acquisition. The trial protocol and statistical analysis plan are shown in Supplement 1.

### Participants

**Figure 1.**
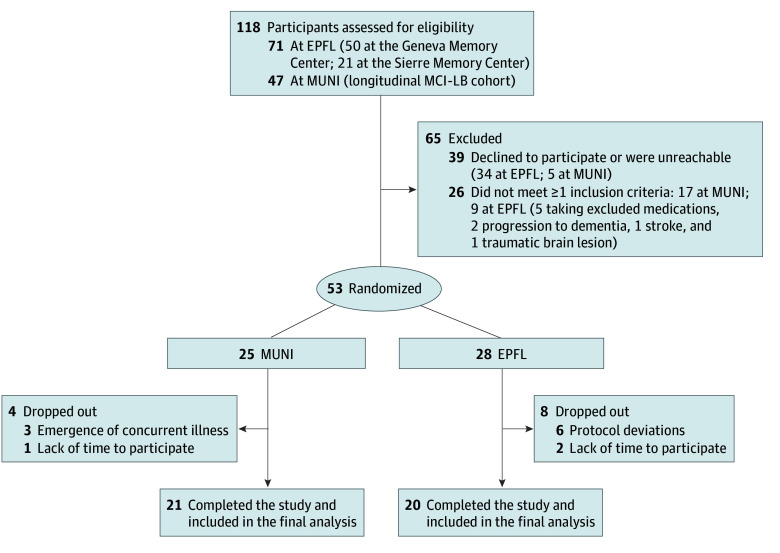
Study Flowchart EPFL indicates École Polytechnique Fédérale de Lausanne; MCI-LB, mild cognitive impairment with Lewy body; MUNI, Masaryk University.

The final clinical sample recruited from August 2023 to October 2024 comprised patients with a diagnosis of amnestic MCI (aMCI) syndrome^18^ recruited at 2 memory centers in Geneva and Sion, Switzerland (aMCI; EPFL cohort), and participants with a diagnosis of MCI-LB recruited in Brno, Czech Republic (MCI-LB; MUNI cohort) from a previously described longitudinal cohort of individuals at risk of DLB^19,20,21^ (Figure 1; see eTables 1 and 2 and the eMethods in Supplement 2 for power analysis, full recruitment details, and aMCI biomarker and medication details). The MCI-LB cohort met clinical research criteria^22^ for probable or possible MCI-LB and Movement Disorder Society level II criteria^23^ for MCI (eTable 1 in Supplement 2), was clinically monitored for 1 to 4 years, was L-dopa and cholinesterase inhibitor naive, and was stratified as having possible MCI-LB (1 core clinical feature) and probable MCI-LB (2 or more core clinical features).^22^ Transcranial ultrasonography of the substantia nigra was available for the majority of the participants with MCI-LB, with 69% showing substantia nigra hyperechogenicity, reflecting excessive iron accumulation and tissue degeneration^24^ (eMethods in Supplement 2). Both patients with MCI-LB and aMCI underwent detailed neuropsychological testing and clinical examination, patient interview, validated scales and questionnaires, and brain magnetic resonance imaging (MRI) assessment (see eMethods in Supplement 2 for details and cutoff scores). To better characterize the clinical population, we also recruited HCs.

Inclusion criteria for the HCs were normal cognitive performance assessed by baseline neuropsychological evaluation, age older than 60 years, right-handedness, and absence of clinical features of prodromal DLB. Exclusion criteria included dementia, major psychiatric disorder or other neurological disease, severe or repeated head injury, noncompensated systemic or oncological disease, and presence of MRI-incompatible material. Patients’ characteristics are listed in the Table.

**Table.** Demographic Characteristics of Cohorts

Characteristic	HC (n = 20)	MCI-LB (n = 21)	aMCI (n = 20)
Sex, No. of participants^a^			
Male	10	5	9
Female	10	16	11
Age, mean (SD), y	69.50 (4.42)	71.05 (6.84)	72.30 (7.50)
Education, mean (SD), y	14.45 (3.46)	13.24 (3.01)	12.65 (2.54)
Montreal Cognitive Assessment score, mean (SD)	26.65 (2.23)	25.48 (2.71)	22.45 (1.30)

Abbreviations: aMCI, amnestic MCI; HC, healthy control; MCI-LB, mild cognitive impairment with Lewy body.

^a^ Sex was determined by participant self-report. Gender identity, race, or ethnicity data were not collected.

**Figure 2.**
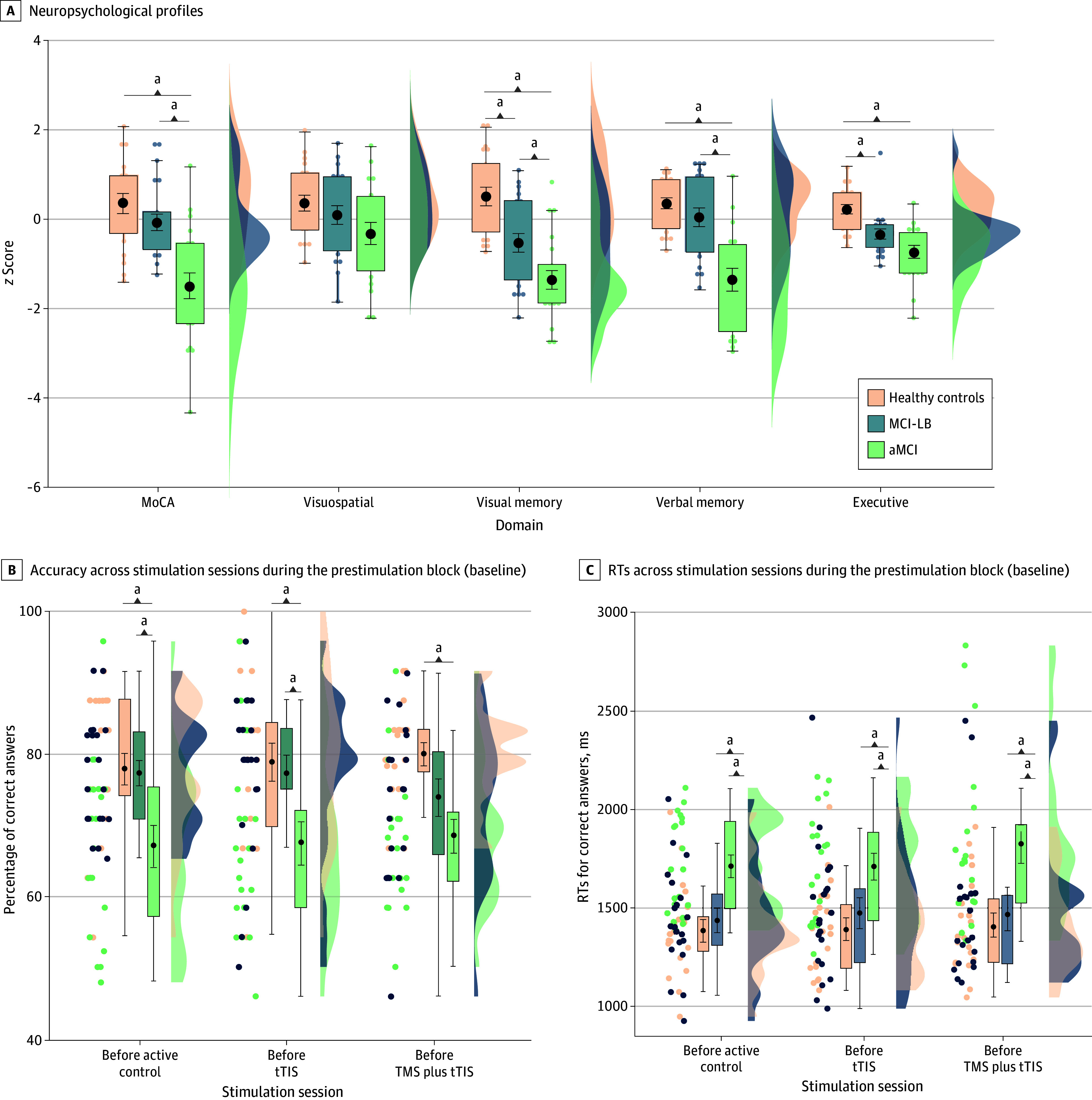
Box and Whisker and Violin Plots of Neuropsychological Profiles Across Mild Cognitive Impairment (MCI) Cohorts and Baseline Performances A, Patients with amnestic MCI (aMCI) were generally more impaired compared with healthy controls (HCs) and patients with MCI with Lewy bodies (MCI-LB). Patients with MCI-LB showed selective impairment in executive functions and visual memory. B, At baseline, patients with aMCI showed significantly lower accuracy and higher reaction times (RTs) than HC and MCI-LB groups, whereas no differences were observed between the HC and MCI-LB groups. For a full description of results, see eResults in Supplement 2. Box plots display group means (dots within boxes) with SDs (lines within boxes). Tops of boxes indicate 75th percentiles, bottoms of boxes denote 25th percentiles, error bars outside boxes denote 95% CIs, and black dots denote outliers. The shaded areas represent violin plots, illustrating the distribution and density of the data within each group for a given metric. MoCA indicates Montreal Cognitive Assessment; TMS, cerebellar transcranial magnetic stimulation; tTIS, striatal transcranial temporal interference stimulation. ^a^*P* < .05.

Hippocampal atrophy and white matter hyperintensities were rated on T1-weighted and T2-weighted MRIs using validated visual rating scales by 2 of 4 independent trained raters (A.Š., K.Š., D.O., or E.V.), with no cohort differences (eMethods and eFigure 1 in Supplement 2). At baseline, we assessed global cognition, episodic memory, visuospatial, and executive functions. Neuropsychological profiles across cohorts are illustrated in Figure 2A (see eTables 3 and 4 in Supplement 2 for neuropsychological batteries).

### Procedures

During the baseline visit, participants underwent consenting and structural and/or functional MRI (fMRI) (see eMethods in Supplement 2 for details on acquisition and preprocessing). During a second visit, they completed a neuropsychological evaluation (eTable 3 in Supplement 2), active motor threshold determination, and WM task familiarization (eFigure 2B in Supplement 2). Subsequently, participants completed a total of 3 single stimulation sessions (visits 3-5), either receiving a placebo stimulation (active control: control TMS plus control tTIS, with TMS applied over the neck surface 5 cm below the cerebellar target and tTIS delivering high-frequency stimulation), active striatal tTIS with control TMS, or combined active cerebellar TMS and striatal tTIS (eFigures 2C, 2D, and 3 in Supplement 2). The order of the conditions was pseudorandomized and counterbalanced across participants (eMethods in Supplement 2). The study employed a quadruple-blinding procedure: participants and tTIS and experimental operators were blinded to the stimulation conditions, and data analyses were performed blinded (or partially blinded) to the stimulation conditions. During the TMS protocol, TMS operators were not blinded. However, participants and tTIS and experimental operators were blinded to the TMS stimulation condition. The WM task was performed at baseline (Figure 2B; eTables 5 and 6 in Supplement 2), during stimulation (ie, online performance), and after the stimulation ceased (ie, poststimulation performance). Furthermore, in the MCI-LB cohort, we acquired a rs-fMRI, both before and after stimulation (eFigure 2A in Supplement 2). For details concerning the WM task, TMS and tTIS procedures, and stimulation order allocation, see the eMethods in Supplement 2.

### Outcomes

The primary outcomes were the changes in accuracy and mean correct answer reaction times (RTs) during the online WM performance, expressed as normalized scores of each participant’s prestimulation (ie, baseline) performance. Secondary end points included poststimulation changes in accuracy and RTs, and changes in resting functional connectivity for the MCI-LB cohort before and after stimulation. Baseline MRI parameters—rs functional connectivity and gray matter volumes in regions involved in WM performance—were assessed in both cohorts and defined as modulatory variables of the outcomes.

### Behavioral Analysis

All behavioral analyses were conducted separately for distraction and high-load task conditions (eFigure 2B and eMethods in Supplement 2), as these engage distinct WM mechanisms. Online analyses examined performance during tTIS, which was delivered following the TMS session, whereas offline analyses focused on poststimulation performance. For further details, see eMethods in Supplement 2. To address the heterogeneity observed in WM performance across cohorts, we adopted an exploratory, data-driven clustering approach, to assess stimulation effects within subgroups defined by baseline cognitive performance (eMethods and eFigure 5 in Supplement 2).

### Independent Component Analysis–Based rs-fMRI Connectivity Analysis

Connectivity analyses were restricted to the MCI-LB group, which showed significant behavioral effects following cerebellar TMS and striatal tTIS during trials involving a distractor. We compared this condition with the active control to examine whether (1) changes in connectivity (prestimulation vs poststimulation) and (2) baseline connectivity were associated with stimulation-induced behavioral changes. We identified rs brain networks involved in WM, including the basal ganglia brain network (eFigure 6 in Supplement 2), using independent component analysis of the preprocessed rs-fMRI data. The *z* values from the bilateral striatum and its subregions (bilateral caudate and/or putamen) were extracted from this component. See eMethods in Supplement 2 for preprocessing, motion exclusions, and full independent component analysis details.

### Structural Imaging Analysis

Volumetric measures were derived from T1-weighted structural images with FreeSurfer version 7.4.1,^25^ using the automatic segmentation function. Gray matter volumes from the left and right lateral frontal, superior temporal, and lateral parietal cortices were summed to calculate the cortical total volume (CTV).^26^ Volumes of specific regions of interest were extracted (hippocampus, caudate, putamen, striatum, and cerebellum). For regression analyses, region of interest volumes were normalized by the CTV.

### Statistical Analysis

For primary outcomes, we used linear mixed-effects models (LMMs) to examine the main effects of stimulation condition and block on online accuracy and RTs, with participants as a random intercept using an unstructured variance-covariance matrix. Statistical significance was defined as 2-sided *P* < .05. Significant effects were followed by posthoc comparisons with the Tukey correction and effect sizes calculation. In parallel, we fitted bayesian mixed-effects models to assess the strength of evidence for the respective effects of the stimulation condition (eMethods in Supplement 2).

For secondary outcomes, we used analogous linear mixed-effects models with posthoc comparisons where relevant. We examined associations between connectivity, brain structure, and behavioral changes using correlation and robust regression analyses (eMethods in Supplement 2). Data were analyzed with R statistical software version 4.4.1 (R Project for Statistical Computing).

## Results

### Enrollment and Patient Characteristics

The sample for the final analysis included 41 patients with MCI, including 21 with MCI-LB (16 female [76%]; mean [SD] age, 71.05 [6.84] years) and 20 with aMCI (11 female [55%]; mean [SD] age, 72.30 [7.50] years). There were 20 HCs, including 10 at MUNI and 10 at EPFL (10 female [50%]; mean [SD] age, 69.50 [4.42] years). See the Table and eTable 7 in Supplement 2 for details.

### Behavioral Results

**Figure 3.**
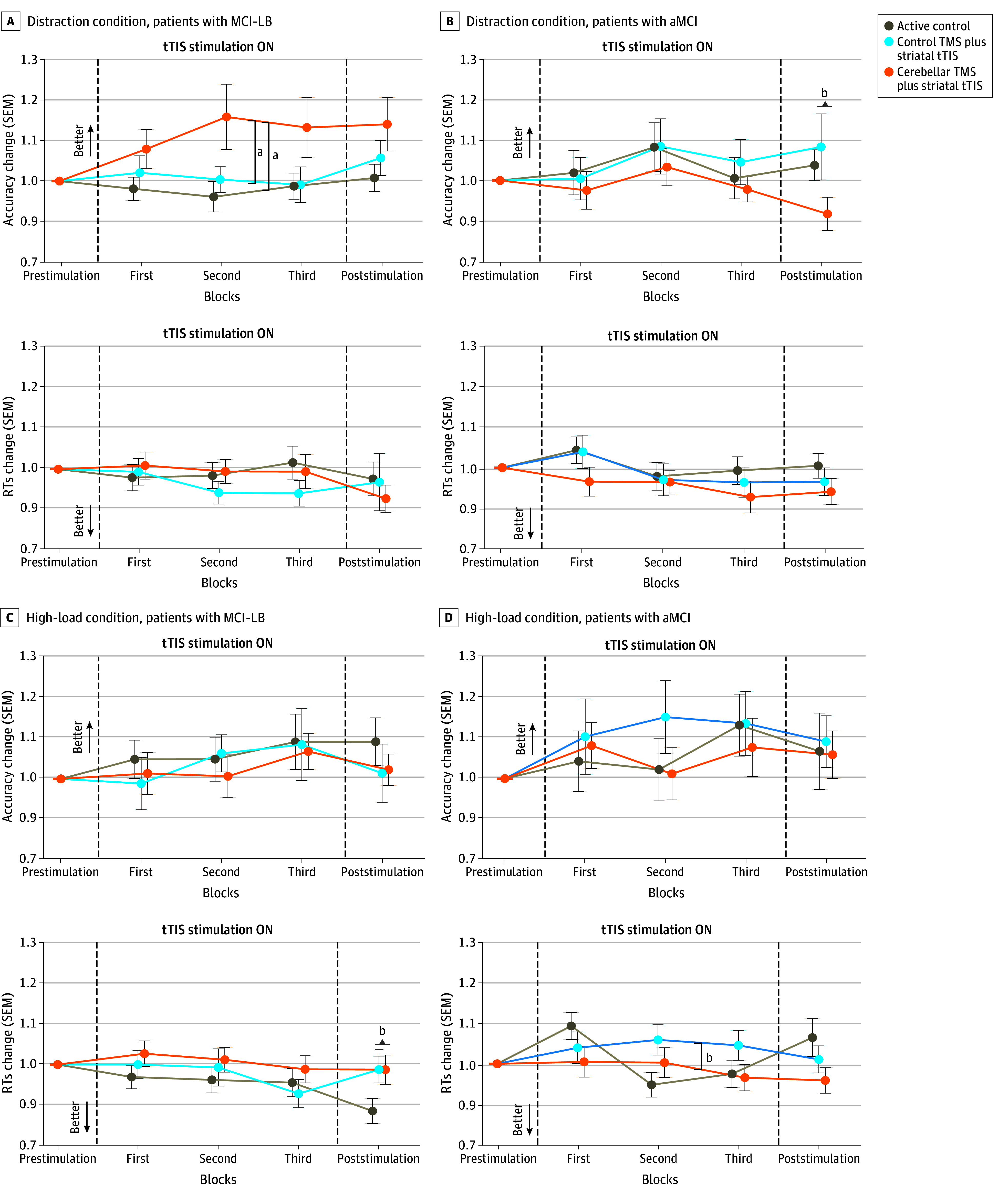
Line Graphs of Stimulation Effects on Visual Working Memory Performance in Mild Cognitive Impairment (MCI) Cohorts Relative changes in accuracy and reaction times (RTs) were calculated compared with each participant’s prestimulation performance (for means and 95% CIs, see eTables 7-10 in Supplement 2). Lines represent group means across blocks, with error bars indicating SEM. aMCI indicates amnestic MCI; LB, Lewy body; TMS, cerebellar transcranial magnetic stimulation; tTIS, striatal transcranial temporal interference stimulation. ^a^*P* < .05. ^b^*P *for trend <.10.

In patients with MCI-LB (Figure 3A; eTables 5-10 in Supplement 2), the factor stimulation had a significant main effect on accuracy during distraction trials (*P* < .001; pη^2^ = 0.11), whereas the interaction between stimulation and block was not significant. In the posthoc analysis (Tukey-adjusted for multiple comparisons, all reported *P* values are adjusted), cerebellar TMS plus striatal tTIS (mean normalized accuracy score, 1.12; 95% CI, 1.06-1.19) yielded higher accuracy compared with both active control (mean normalized accuracy score, 0.97; 95% CI, 0.90-1.04; *P* < .001; *d* = −0.77) and control TMS plus striatal tTIS (mean normalized accuracy score, 1.01; 95% CI, 0.94-1.07; *P* = .004; *d* = −0.60). This finding was corroborated by bayesian analysis (eResults and eFigure 7 in Supplement 2). No stimulation, block, or interaction effects were observed for RTs in distraction trials or for accuracy or RTs in the high-load condition.

For patients with aMCI (Figure 3B), the factor stimulation and the stimulation by block interaction did not show a significant main effect on RTs in high-load condition (see eResults and eFigure 8 in Supplement 2 for details). No significant effects on accuracy or RTs were observed in the high-load condition or during the distraction condition. In poststimulation analyses, no significant results were observed either for MCI-LB or aMCI groups. For full details, see the eResults and eTables 5 to 10 in Supplement 2.

For details concerning the HC cohort, refer to eResults and eFigure 9 in Supplement 2. No harms or serious adverse events occurred during the study. Perceived sensations, stimulation effects on attention, fatigue, sleep, decision-making, emotion, and blinding efficacy were only evaluated in the EPFL cohort (eResults and eFigures 10-13 in Supplement 2).

### Striatal Connectivity and Its Association With Stimulation-Induced Behavioral Effects

Baseline *z* values from the bilateral striatum (active control, 5.46 [95% CI, 4.36-6.57] vs cerebellar TMS and striatal tTIS, 5.40 [95% CI, 4.27-6.54]; *P* = .92) and its subregions—caudate (active control, 7.01 [95% CI, 5.0-9.02] vs cerebellar TMS and striatal tTIS, 7.02 [95% CI, 4.97-9.08]; *P* = .99) and putamen (active control, 3.92 [95% CI, 2.95-4.90] vs cerebellar TMS and striatal tTIS, 3.82 [95% CI 2.82-4.82]; *P* = .85)—did not significantly differ between the cerebellar TMS plus striatal tTIS and active control sessions in the MCI-LB group. To elucidate neural correlates of stimulation-induced effects, we first assessed whether changes in striatal connectivity (prestimulation vs poststimulation) were associated with changes in accuracy. However, no significant correlations emerged. We then tested whether prestimulation connectivity was associated with stimulation-induced effects in accuracy in the MCI-LB group. There was a significant negative correlation between prestimulation connectivity of the bilateral putamen and accuracy changes under distraction condition (*r* = −0.66; *P* = .03) (eFigure 14 and eTable 11 in Supplement 2), suggesting that lower initial connectivity was associated with greater behavioral improvement following cerebellar TMS plus striatal tTIS. No such association was present for the active control condition.

### Structural Imaging Results

We did not observe any significant differences in the CTV-normalized volumes of the examined regions (hippocampus, caudate, putamen, striatum, and cerebellum) across the 2 cohorts. Regression analyses assessing the association between regional brain volumes and behavioral performance across stimulation conditions did not find any significant correlation in the MCI-LB cohort. In the aMCI group, the normalized cerebellar volume was negatively correlated with RTs during cerebellar TMS plus striatal tTIS in trials involving a distractor (*R*^2^ = 0.27; β = −1.47; 95% CI, −1.50 to −1.43; *P* = .02) (eFigure 15A in Supplement 2) and with control TMS plus striatal tTIS in high-load trials (*R*^2^ = 0.24; β = −1.09; 95% CI, −1.13 to −1.06; *P* = .03) (data not shown). More preserved cerebellar volume was associated with faster RTs during cerebellar TMS plus striatal tTIS in the detection of distractors, and with faster RTs in high-load trials during control TMS plus striatal tTIS. CTV-normalized cerebellar volume also correlated negatively with the difference in RTs between the cerebellar TMS plus striatal tTIS and active control condition (*R*^2^ = 0.39; β = −1.98; 95% CI, −2.02 to −1.95; *P* = .004) (eFigure 15B in Supplement 2).

## Discussion

In this randomized, quadruple-blind, placebo-controlled crossover trial, we provide the first preliminary evidence, to our knowledge, that synergistic dual-target noninvasive stimulation of the left inferior cerebellar hemisphere and the striatum, can transiently enhance WM in patients with MCI-LB, specifically during trials involving a distractor. We demonstrate the feasibility, safety and efficacy of this dual-target neuromodulation strategy to enhance higher cognitive functions in patients at high risk of dementia.

Striatal activity plays a crucial role in WM via its reciprocal interaction with the prefrontal cortex^27,28^ and is tightly regulated by dense cerebellar connections.^6,9^ The striatum is affected early in the course of Parkinson disease and DLB,^29^ with dopaminergic depletion and loss of striato-cortical connections being specifically associated with executive dysfunction.^21^ In DLB, early degeneration affects the putamen and fronto-striatal medium spiny neurons, which is associated with reduced dopamine transporter single-photon emission computed tomography uptake.^29,30^ On the other hand, the cerebellum is largely preserved^31^ and is tightly connected via dento-thalamic connections to a larger network including the striatum.^9^ Therefore, we hypothesized that the cerebellum could represent a promising target to modulate WM through its striatal connections. Recent studies have shown cognitive benefits by noninvasively targeting cortical areas of the frontoparietal network with transcranial electrical stimulation^32^ or subcortical areas such as the cerebellum with TMS.^33,34,35,36^ In the current work, sequentially applying cerebellar TMS followed by striatal tTIS using plasticity-supporting, long-term potentiation–like patterned stimulation protocols, enhanced WM performance in patients with MCI-LB. These results suggest that cerebellar TMS might have supported striatal activity during tTIS through reciprocal striato-cerebellar connections, as tTIS and the task were performed in the time window of expected maximal effect of intermittent theta burst stimulation, between 10 and 30 minutes after stimulation,^37^ possibly enhancing local dopamine release and modulating the medium spiny neurons output during the distractor selection. Indeed, striatal dopamine modulates putaminal connectivity during WM-related processes and striato-cortical interactions.^38^ Accordingly, we found that weaker putaminal connectivity was associated with greater accuracy gains during cerebellar TMS plus striatal tTIS, suggesting that less-integrated striatal networks may benefit from stimulation via enhanced dopaminergic modulation of specific neuronal populations (ie, medium spiny neurons). Although promising, this hypothesis must be validated in upcoming studies in prodromal DLB.

In contrast, in the aMCI cohort, cerebellar TMS plus striatal tTIS did not produce any significant behavioral results, as we observed only a trend toward shorter online RTs in the high-load condition compared with control TMS plus striatal tTIS (see eResults in Supplement 2). Notably, faster performance was associated with more preserved cerebellar volumes, during both active stimulation conditions, which potentially suggest a more prominent role of cerebellar modulation in aMCI. Nonetheless, this hypothesis requires formal investigation in future studies.

More generally, the absence of a significant behavioral effect in patients with aMCI, despite similar levels of executive dysfunction compared with patients with MCI-LB, may result from the greater cognitive impairment and the single-session design, which might have been insufficient to produce a significant behavioral change.^35^ Thus, another aspect to consider for future studies might be the disease stage (corroborated with fluid and/or imaging biomarkers).

A key challenge in such studies is the pronounced interindividual variability in baseline WM performance, which likely contributes to the heterogeneity of the stimulation response.^39^ To address this, we clustered patients by their individual baseline WM performance, identifying 3 subgroups characterized by different WM and cognitive profiles, cerebral brain volumes (eResults and eFigures 5 and 16 in Supplement 2), and stimulation responses (eResults and eFigure 17 in Supplement 2). Although limited by cluster sizes (n = 11 to 17), this exploratory analysis suggests baseline WM performance as a potential predictor of stimulation response, which, if validated, could emerge as an additional tool for patient selection and treatment personalization.

### Limitations

This study followed a rigorous protocol, demonstrated the safety and feasibility of circuit-based multimodal noninvasive stimulation, and provided evidence that coordinated targeting of subcortical WM networks may offer a promising strategy to enhance cognition in MCI. We note some limitations that warrant consideration. First, the moderate sample size in the single groups may limit the generalizability of the results. Second, the lack of cerebellar TMS plus control tTIS restricts our ability to fully evaluate the cerebellar contributions to WM, although cerebellar iTBS alone failed to show a significant cognitive enhancement to date.^33^ Nevertheless, future studies should investigate the role of cerebellar TMS stimulation in WM, perhaps using a multiple session design. Third, the lack of disease-specific biomarkers for the MCI-LB cohort might have led to the inclusion of patients without LB pathology. The suggested biomarkers are limited by their moderate sensitivity (ie, 59% for dopamine transporter single-photon emission computed tomography in MCI-LB),^40^ and by their restricted accessibility for clinical use in Czech Republic (eg, meta-iodobenzylguanidine cardiac scintigraphy or polysomnography). To minimize this risk, we selected patients following extensive clinical evaluations within a well-described longitudinal cohort.^19,20,21^ Fourth, the single-session nature of the intervention and the lack of follow-up limit our capacity to assess the long-term clinical relevance of the stimulation and should be further investigated in future studies.

## Conclusions

Our findings provided converging evidence for the involvement of both the striatum and the cerebellum in WM and demonstrated that their coordinated modulation through combined cerebellar TMS and striatal tTIS enhanced cognitive performance. Future studies in larger and phenotypically stratified MCI cohorts will be needed to disentangle the distinct and interactive contributions of the striatum and cerebellum to WM. Longitudinal trials incorporating repeated and individualized stimulation protocols will be essential to determine whether these effects can be optimized, sustained over time, and translated into durable clinical benefit for specific neurodegenerative populations.

## Notes

Supplement 1. Trial Protocol and Statistical Analysis Plan

Supplement 2. eMethods. eTable 1. Measures of core clinical features of DLB in the MCI-LB group eTable 2. Molecular biomarkers for aMCI cohort eTable 3. Neuropsychological testing batteries used during baseline assessment of MCI patients and HC eTable 4. Neuropsychological comparisons eFigure 1. Radiological visual rating scales across MCI cohorts eFigure 2. Overview of Study Design, Cognitive Task, Stimulation Protocols, and Modeled Striatal E-Field Distribution eFigure 3. iTBS waveform pattern used during tTIS eFigure 4. Baseline and normalized behavioural performance across MCI cohorts eTable 5. Baseline (Pre-stimulation) Accuracy Across Cohorts and Conditions eTable 6. Baseline (Pre-stimulation) RTs Across Cohorts and Conditions eFigure 5. MCI patient clustering based on baseline performance eFigure 6. ICA component representing basal ganglia network eResults. eTable 7. Normalized Distraction Accuracy Across Blocks and Stimulation Conditions eTable 8. Normalized High-Load Accuracy Across Blocks and Stimulation Conditions eTable 9. Normalized Distraction RT Across Blocks and Stimulation Conditions eTable 10. Normalized High-Load RT Across Blocks and Stimulation Conditions eFigure 7. Posterior predictive checks for the Bayesian model assessing stimulation effects on Accuracy during the distraction condition in the MCI-LB Cohort eFigure 8. Posterior predictive checks for the Bayesian model assessing stimulation effects on RTs during the distraction condition in the aMCI Cohort eFigure 9. Stimulation effects on visuospatial WM performance in HC cohort (n = 20) eFigure 10. Effects of stimulation on attention, fatigue, sleep, decision-making, and emotion in the EPFL MCI cohort eFigure 11. Effects of stimulation on attention, fatigue, sleep, decision-making, and emotion in the EPFL HC cohort eFigure 12. Sensations reported for tTIS by EPFL participants eFigure 13. Evaluation of perceived tTIS sensations and blinding efficacy to tTIS stimulation condition for EPFL participants eFigure 14. Associations between pre-stimulation putaminal connectivity and stimulation-induced behavioural changes in the MCI-LB group eTable 11. Correlations between pre-stimulation basal ganglia connectivity and stimulation-induced changes in Accuracy under distraction in the MCI-LB group eFigure 15. Associations between cerebellar volume and behavioural performance in the aMCI group eFigure 16. Stimulation effects on visuospatial WM performance across patient clusters eFigure 17. Group differences across clusters in baseline neuropsychological performance (a) and demographic variables and regional brain volumes (b) eReferences

Supplement 3. Data Sharing Statement
