# Supplementary material for: Multifocal Noninvasive Deep Brain Stimulation to Enhance Cognition in Mild Cognitive Impairment: A Crossover Trial
Source: JAMA Netw Open. 2026 Jul 6;9(7):e2621756. doi: 10.1001/jamanetworkopen.2026.21756 (PMC13338810; doi:10.1001/jamanetworkopen.2026.21756)
Supplement: Supplement 1. — Trial Protocol and Statistical Analysis Plan [file jamanetwopen-e2621756-s001.pdf]

Clinical Trial Protocol

Multifocal non-invasive deep brain stimulation to enhance cognition in mild cognitive impairment: a multicenter, randomized, placebo-controlled study

Principal Investigators: Irena Rektorová, Friedhelm Cristoph Hummel

TABLE OF CONTENTS

1. Study Title 6
2. Trial Institutions 7
3. Principal Investigators and collaborators 8
    Principal investigators 8
    Collaborators 8
4. Objectives and Background 9
    4.1 Objective: 9
    4.1.1 Primary Objectives 9
    4.1.2 Secondary Objectives 9
    4.2 Background 9
5. Research Hypothesis, Inclusion and Exclusion Criteria, and Sample Size Determination. 10
    5.1 Research Hypothesis 10
    5.2 Inclusion Criteria 10
    5.3 Exclusion Criteria 10
    5.4 Sample Size Determination 10
5. Trial Duration 11
6. Methods 12
    7.1 Trial Design, Randomized Allocation, Blinding 12
    a. Trial Procedures 12
    b. Randomized allocation and Masking 13
7. Observation items and Observation methods 14
8. Predicted Side Effects and Precautions during use 15
    9.1 Intermittent theta burst cerebellar transcranial magnetic stimulation (TMS) 15
    9.2 Transcranial Temporal Interference stimulation (tTIS) 15
    9.3 MRI 15
9. Outcome Measure, Statistical Analysis, and Interpretation 16
11. Statistical analyses of the outcomes 16
11.1 Primary outcomes 16
12. References 17

Trial Protocol

**Objectives**	This proof-of-concept multicenter, randomized trial aims to assess whether dual-target, sequential cerebellar transcranial magnetic stimulation (TMS) and striatal transcranial temporal interference stimulation (tTIS) could enhance working memory in individuals with mild cognitive impairment (MCI), especially in individuals with early striatal degeneration.	
**Trial Institutions**	Masaryk University (MUNI), Czech Republic ;<br>École Polytechnique Fédérale de Lausanne (EPFL), Switzerland.	
**Trial Duration**	from August 2023 to October 2024 (approximately 14 months).	
**Trial design**	randomized, quadruple-blind, placebo-controlled, crossover design.	
**Participants**	Inclusion criteria	MCI:<br>1. age > 18 years old;<br>2. Clinical diagnosis of MCI due to possible or probable Lewy Bodies Dementia[1] or amnestic MCI following core clinical and neuropsychological criteria.[2,3]<br><br>Healthy controls (HC)<br>1. age > 60 years;<br>2. right-handedness;<br>3. absence of clinical features of prodromal DLB;[1]<br>4. Normal performance on baseline cognitive screening.
	Exclusion criteria	1. dementia;<br>2. major psychiatric disorder;<br>3. other neurological disease;<br>4. severe or repeated head injury;<br>5. non-compensated systemic or oncological disease;<br>6. presence of Magnetic Resonance Imaging (MRI)-incompatible material.
	Number of participants	MCI:<br>1. 21 MCI with Lewy bodies (MCI-LB) (MUNI)<br>2. 20 amnestic MCI (aMCI) (EPFL)<br>HC:<br>1. 10 HC (MUNI)<br>2. 10 HC (EPFL)
**Methods**	Target selection	Striatum: the bilateral striatum was targeted by placing the electrodes on the skin according to the 10-10 EEG system: one anterior pair at F3-F4 and one posterior pair at TP7-TP8 positions, based on previous published work.[4]<br><br>Cerebellum: MNI coordinates (MNI = -30 -74 -51) targeting the left VIIb/VIIIa lobule were projected onto the patient T1-weighted MRI, based on previous imaging[5] and stimulation works.[6] This point was used as a target using a neuronavigated TMS during the active stimulation sessions and as a reference to calculate the neck target 5cm vertically downward during the control stimulation sessions.
	Stimulation protocols	tTIS (delivered during the realisation of the cognitive task):<br>1. Active condition ($tTIS_{str}$): delivering an intermittent theta-burst pattern (iTBS) to the bilateral striatum, as described in a previous published work.[4]<br>2. Control condition ($tTIS_{control}$): high-frequency stimulation at 2kHz, as described in a previous published work.[4]<br><br>TMS (delivered 10 minutes before the beginning of tTIS):<br>1. Active condition (TMS_{cb}): delivering an iTBS pattern at 80% of each participant's active motor threshold (aMT), targeting the left inferior cerebellum (MNI = -30 -74 -51), based on previous neuroimaging[5] and TMS studies.[6] The iTBS protocol consisted of delivering ten bursts, each made

		up of three pulses at 50Hz, repeated at 5Hz. This block was repeated twenty times every 10 sec to give a total of 600 pulses, as previously described.[7]<br>2. Control condition ($TMS_{control}$): the same iTBS pattern at the same individual intensity was delivered in other separate sessions targeting the neck.
	Stimulation conditions	Session A: $TMS_{control}$+ $tTIS_{control}$<br>Session B: $TMS_{control}$+$tTIS_{str}$<br>Session C: TMS_{cb}+$tTIS_{str}$<br><br>The order of the session was pseudorandomized by two persons who were not involved in the study.
	Masking and Blinding	The allocation sequence was independently generated by two individuals who were not involved in the study, data collection, or analyses (one at EPFL, one at MUNI). The sequence was concealed from participants, care providers, investigators performing the stimulation, outcome assessors, and staff performing the analyses.<br><br>The order of the stimulation session was pseudo-randomized and participants, tTIS and experimental operators were blinded to the stimulation conditions, and data analyses were performed blinded (or partially blinded) to the stimulation conditions.
	Devices	MUNI: Deymed DuoMAG XT stimulator (non-air cooled). Frameless stereotactic neuro-navigation system: BrainSight (BrainSight Rogue Research Inc., Canada);<br><br>EPFL: MC-B70 coil connected to the Magventure MagPro X100 stimulator (MagVenture, Farum, Denmark). Frameless stereotactic neuro-navigation system: Localite (Localite GmbH, Bonn, Germany).
	MRI	MRI was acquired with 3.0T Magnetom Siemens Prisma at both centers.<br><br>Structural MRI sequence: T1 MPRAGE. TR 2300 ms; TE 2.96 ms; voxel size 1 × 1 × 1 mm; FoV 256 × 256 mm; flip angle 9°; 192 transversal slices<br><br>Functional resting state MRI sequence: gradient-echo, T2 echo-planar. TR 1250 ms; TE 32 ms; voxel size 2 × 2 × 2 mm; FoV 224 mm; flip angle 65°; 76 transversal slices; 480 scans; multiband factor 4.
	Baseline neuropsychological evaluation	MUNI:<br>1. Montreal Cognitive Assessment (MoCA)[8];<br>2. Unified Parkinson's Disease Rating Scale Motor Examination (UPDRS III)[9];<br>3. Mayo Fluctuation Scale (MFS)[10];<br>4. Geriatric Depression Scale (GDS)[11];<br>5. REM sleep behavior disorder screening questionnaire (RBDSQ)[12];<br>6. Neuropsychiatric Inventory (NPI)[13];<br>7. Epworth Sleepiness Scale (ESS)[14];<br>8. Brief visuospatial memory test-revised[15];<br>9. Philadelphia Verbal Learning Test[16];<br>10. Wechsler Adult Intelligence Scale−III: Letter-Number Sequencing and Digit Symbol Substitution[17];<br>11. Semantic and phonemic verbal fluency[18];<br>12. Picture arrangement test[17];<br>13. Judgment of Line Orientation.[19]

		EPFL:<br>1. Montreal Cognitive Assessment (MoCA)[8];<br>2. Brief Visuospatial Memory Test-revised[15];<br>3. Hopkins Verbal Learning Test-revised[20];<br>4. Wechsler Adult Intelligence Scale−IV: Letter-Number Sequencing and Digit Symbol Substitution[21];<br>5. Semantic and phonemic verbal fluency[22];<br>6. Color Trail Making Test A and B[23];<br>7. Rey-Osterrieth Figure - Copy[24];<br>8. Line Bisection Test.[25]<br><br>All participants also completed the Pittsburgh Sleep Quality Index (PSQI)[26] and the Functional Activities Questionnaire (FAQ).[27]
	Cognitive task	We adopted a computerized visuospatial working memory (WM) task adapted from Harrington *et al.* 2020[28], involving low-load, distractors and high-load trials.
**Outcomes**	Primary outcomes	Changes in Accuracy and Mean Reaction Times for correct answers (RTs) during the online WM performance, expressed as normalized scores of each participant's pre-stimulation (i.e., baseline) performance.
	Secondary outcomes	1. post-stimulation changes in Accuracy and RTs ;<br>2. changes in resting functional connectivity for the MCI-LB cohort before and after stimulation.
	Modulators of the outcomes	1. baseline MRI resting-state functional connectivity.<br>2. MRI gray matter volumes in regions involved in WM performance.

1. Study Title

Multifocal non-invasive deep brain stimulation to enhance cognition in mild cognitive impairment: a multicenter, randomized, placebo-controlled study.

2. Trial Institutions

Name	Location	Telephone	e-mail
Masaryk University	Brno, Czech Republic	+420 54949 7825	irena.rektorova@ceitec.muni.cz
École Polytechnique Fédérale de Lausanne	Geneva, Switzerland	+41 21 693 54 40	friedhelm.hummel@epfl.ch

3. Principal Investigators and collaborators

Principal investigators

Name	Institution	Department	Position
Irena Rektorovà	Masaryk University	First Department of Neurology	Professor
Friedhelm Cristoph Hummel	École Polytechnique Fédérale de Lausanne	Defitech Chair of Clinical Neuroengineering	Professor

Collaborators

Name	Institution	Department	Position
**Umberto Nencha**	École Polytechnique Fédérale de Lausanne	Defitech Chair of Clinical Neuroengineering	Researcher
**Monika Pupíkova**	Masaryk University	Central European Institute of Technology	Researcher
**Margaux Di Natale**	École Polytechnique Fédérale de Lausanne	Defitech Chair of Clinical Neuroengineering	Researcher
**Pablo Maceira-Elvira**	Wyss Center for Bio- and Neuroengineering	Campus Biotech Geneva	Researcher
**Martin Gajdoš**	Masaryk University	Central European Institute of Technology	Researcher
**Elena Beanato**	École Polytechnique Fédérale de Lausanne	Defitech Chair of Clinical Neuroengineering	Researcher
**Stavriani Skarvelaki**	École Polytechnique Fédérale de Lausanne	Defitech Chair of Clinical Neuroengineering	Researcher
**Rebecca Jones**	École Polytechnique Fédérale de Lausanne	Defitech Chair of Clinical Neuroengineering	Researcher
**Isabel Ericson**	École Polytechnique Fédérale de Lausanne	Defitech Chair of Clinical Neuroengineering	Researcher
**Martin Lamoš**	Masaryk University	Central European Institute of Technology	Researcher
**Andrea Nuti**	École Polytechnique Fédérale de Lausanne	Defitech Chair of Clinical Neuroengineering	Researcher
**Fabienne Windel**	École Polytechnique Fédérale de Lausanne	Defitech Chair of Clinical Neuroengineering	Researcher
**David Ondráček**	Masaryk University	Central European Institute of Technology	Researcher
**Adam Šimo**	Masaryk University	Central European Institute of Technology	Researcher
**Ela Vojtková**	Masaryk University	Central European Institute of Technology	Researcher
**Klára Špunarová**	Masaryk University	Central European Institute of Technology	Researcher
**Vincent Alvarez**	Hôpital du Valais	Neurology	Professor
**Oana Simionescu**	Hôpital du Valais	Neurology	Clinician
**Giovanni Battista Frisoni**	Geneva Memory Center	Rehabilitation and Geriatrics	Professor
**Estelle Raffin**	Grenoble Alpes University	Laboratoire de Psychologie et NeuroCognition	Researcher

4. Objectives and Background

4.1 Objective:

This proof-of-concept multicenter, randomized trial aims to assess whether dual-target, sequential neuromodulation approach combining cerebellar transcranial magnetic stimulation (TMS) and striatal transcranial temporal interference stimulation (tTIS) can enhance working memory (WM) in individuals with mild cognitive impairment (MCI), especially in individuals with early striatal degeneration.

4.1.1 Primary Objectives

Change in Accuracy and Mean Reaction Times for correct answers (RTs) during online WM task performance, expressed as normalized scores of each participant's pre-stimulation (i.e., baseline) performance.

4.1.2 Secondary Objectives

Change in Accuracy and RTs in the performance of the same working memory task, assessed after the end of tTIS stimulation.

In the MCI-LB cohort, changes in the resting-state functional connectivity were assessed before and after stimulation.

4.2 Background

Executive functions and particularly WM play a crucial role in daily life and are frequently impaired in patients with mild cognitive impairment (MCI)[29], constituting one of the driving factors that compromise quality of life and autonomy. WM specifically relies on a vast brain network of cortical and subcortical regions. Among the different subcortical structures implicated in executive functions, both striatum and cerebellum have been linked to WM performance with complementary roles[30–32]. Striatum is variably affected in the course of different neurodegenerative diseases, while in contrast, the cerebellum has been associated with cognitive resilience and brain reserve[33], making it a promising target for interventions for cognitive enhancement.

Hence, leveraging reciprocal striato-cerebellar connections[34], in this multi-center, randomized, placebo-controlled proof-of-concept trial, the investigators implemented a sequential multifocal plasticity-inducing stimulation of the striatum and the cerebellum to enhance WM in MCI patients.

The investigators applied an excitatory intermittent theta burst (iTBS) over the left inferior cerebellar hemisphere, together with transcranial temporal interference stimulation (tTIS) using a similar pattern to focally neuromodulate the striatum in MCI patients.

The investigators hypothesized that sequential stimulation of multiple subcortical brain regions supporting WM (i.e., the striatum and cerebellum), using transcranial temporal interference stimulation (tTIS) and transcranial magnetic stimulation (TMS), could enhance WM performance in MCI patients. Moreover, by combining different stimulation techniques with multimodal neuroimaging and computational modeling, the investigators aimed to gain a better mechanistic understanding of how different non-invasive brain stimulation (NIBS) approaches act on the brain and improve cognitive functions.

5. Research Hypothesis, Inclusion and Exclusion Criteria, and Sample Size Determination.

5.1 Research Hypothesis

1. We hypothesised that cerebellar TMS followed by striatal tTIS would enhance working memory (WM) performance, particularly in MCI with Lewy Bodies (MCI-LB), during a visual WM task manipulating distractor inhibition and memory load.

2. We expected a differential stimulation response between MCI-LB and amnestic MCI (aMCI), possibly also related to the task condition (distractor selection vs high load).

3. We explored whether individual resting-state functional connectivity and gray matter volume of stimulated regions might influence the behavioural effects of the stimulation.

5.2 Inclusion Criteria

MCI:

1. age > 18 years old;
2. Clinical diagnosis of MCI due to possible or probable Lewy Bodies Dementia[1] or amnestic MCI following core clinical and neuropsychological criteria.[2,3]

Healthy controls (HC):

1. age > 60 years;
2. right-handedness;
3. absence of clinical features of prodromal DLB;[1]
4. Normal performance on baseline cognitive screening.

5.3 Exclusion Criteria

1. Dementia;
2. Major psychiatric disorder;
3. Other neurological disease;
4. Severe or repeated head injury;
5. Non-compensated systemic or oncological disease;
6. Presence of Magnetic Resonance Imaging (MRI)-incompatible material.

5.4 Sample Size Determination

The sample size for the current study was calculated with a power analysis based on prior tTIS research targeting the striatum ($d = 0.76$)[4] adjusted for an additional stimulation condition ($\alpha = 0.02$, $\beta = 0.8$). This analysis indicated that 22 participants per group would be required, which we closely matched. It is noteworthy that this study serves as a proof-of-concept to help determine sample size and evaluate changes in cognitive function for future confirmatory clinical trials.

5. Trial Duration

Duration from start to completion: 27 months;
Participant recruitment period: 14 months;
Clinical observation and trial execution period: 14 months;
Statistical analysis and report writing period: 13 months.

6. Methods

7.1 Trial Design, Randomized Allocation, Blinding

Primary outcomes

1. Study Design: crossover design;
2. Allocation: pseudorandom assignment to stimulation order;
3. Changes in Accuracy and mean Reaction Times (RTs) for correct answers during the performance of the working memory task;
4. Masking: evaluators and participants were unaware of the sequence allocation.

Secondary outcomes

1. Study Design: crossover design;
2. Allocation: pseudorandom assignment to stimulation order;
3.1 Change in Accuracy and RTs in the same working memory task, assessed after the end of tTIS stimulation.
3.2 In the MCI-LB cohort, changes in the resting-state functional connectivity were assessed before and after stimulation.
4. Masking: evaluators and participants were unaware of the sequence allocation.

a. Trial Procedures

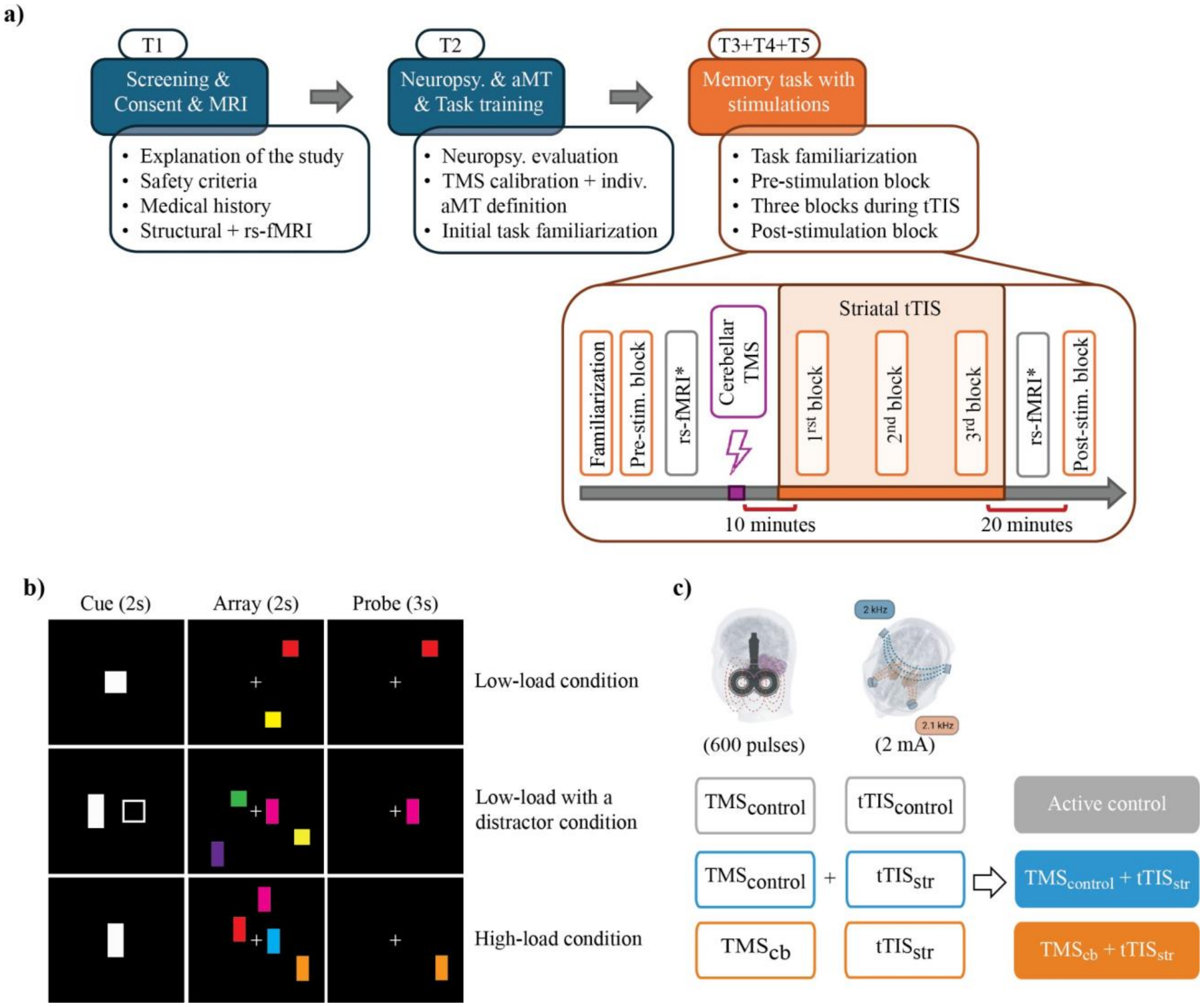

The study included 3 groups:
- 21 MCI with Lewy Bodies (MCI-LB) patients;
- 20 amnestic MCI (aMCI) patients;
- 20 Healthy Controls (HC) elderly patients.

A total of 41 MCI patients underwent a total of 5 sessions to complete the study. During the baseline visit (T1), participants underwent consenting and structural/functional MRI. During a second visit (T2) they completed a baseline neuropsychological evaluation, active Motor Threshold (aMT) determination, and working memory (WM) task familiarization. Subsequently, participants completed a total of three single stimulation sessions (T3-T5), either receiving a placebo stimulation (Active control: $TMS_{control}$+$tTIS_{control}$; TMS applied over neck surface 5 cm below the cerebellar target; tTIS delivering high-frequency stimulation), active striatal tTIS with control TMS ($TMS_{control}$+$tTIS_{str}$), or combined active cerebellar TMS and striatal tTIS (TMS_{cb}+$tTIS_{str}$). The order of the conditions was pseudorandomized and counterbalanced across participants. The cognitive task was performed at baseline, during stimulation, and after the stimulation ceased. Furthermore, in the MCI-LB cohort a resting-state fMRI was realized both before and after stimulation.

The HC cohort underwent the same procedure and was analysed separately, although it was used as a comparator of the clinical cohorts and a formal hypothesis was not formulated for this specific group.

b. Randomized allocation and Masking

The allocation sequence was independently generated by two individuals who were not involved in the study, data collection, or analyses (one at EPFL, one at MUNI). The sequence was concealed from participants, care providers, investigators performing the stimulation, outcome assessors, and data analysts.

c. Blinding

The study employed a quadruple-blinding procedure: participants, tTIS and experimental operators were blinded to the stimulation conditions, and data analyses were performed blinded (or partially blinded) to the stimulation conditions. During the TMS protocol, TMS operators were not blinded. However, participants, tTIS and experimental operators were blinded to the TMS stimulation condition.

d. Stimulation Protocols

i. Transcranial Magnetic Stimulation (TMS)

Calibration of stimulation intensity: single-pulse TMS was applied to the left $M1_{HAND}$ to determine the resting and active motor thresholds (rMT and aMT) at the right first dorsal interosseous (FDI) muscle. The coil was positioned posterior to anterior with a 45 degrees angle towards the midsagittal line to determine the hotspot for activating the FDI muscle. aMT was defined as the lowest intensity of stimulation output that eliciting motor evoked potentials (MEPs) of ~200 µV in 50% of trials, while the right FDI muscle was contracted isometrically at 10-20% of maximum voluntary contraction. TMS was applied using an iTBS protocol at 80% of each participant's aMT, targeting the left inferior cerebellum (MNI = -30 -74 -51) (TMS_{cb} based on previous neuroimaging[5] and TMS studies[6]).

Coil positioning: during iTBS delivery, the coil was oriented vertically with the handle pointing upwards, targeting the left inferior cerebellum (TMS_{cb}) (MNI = -30 -74 -51). For the Active control condition ($TMS_{control}$), the same coil and stimulation parameters were used, but the coil was positioned 5 cm vertically downwards from the target and placed on the neck surface with the handle pointing upwards.

Stimulation parameters:
- Active stimulation: iTBS pattern over the cerebellum (TMS_{cb}), delivering 10 bursts, each made up of three pulses at 50Hz, repeated at 5Hz. This block was repeated 20 times every 10 sec to give a total of 600 pulses, as previously described.[7]
- Control stimulation: iTBS pattern over the neck target ($TMS_{ccontrol}$), using the same parameters for the Active stimulation condition.

ii. Transcranial Temporal Interferences Stimulation (tTIS)

Calibration of stimulation intensity: evaluation of perceived sensations was performed with the intensity per channel increasing step-wise, i.e. 0.5 > 1 > 1.5 > 2 mA.[35–37] Stimulation was performed for both active and control condition with an amplitude of 2mA peak-to-baseline.

Electrode positioning: two pairs of circular rubber electrodes (3cm^2 in surface) were placed on the skin according to the 10-10 EEG system: one anterior pair at F3-F4 and one posterior pair at TP7-TP8 positions, based on previous work.[35]

Stimulation parameters:

- Active stimulation: iTBS pattern targeting the bilateral striatum ($tTIS_{str}$). iTBS consisted of bursts of three pulses at 100 Hz, repeated every 200 ms, for 2 sec. To generate this pattern using tTIS ($tTIS_{str}$), the first channel continuously delivered a 2 kHz current (f_1), while the second channel alternated between f_1=2 kHz and $f_1+\Delta f$=2.1 kHz every 200 ms. This alternation lasted 30 ms during each 2-sec train, producing the 100 Hz bursts. During the interburst and intertrain intervals (8 sec), non-amplitude-modulated HF stimulation was applied.
- Control condition : high-frequency stimulation ($tTIS_{control}$), as previously described.[35,36] Two oscillatory HF currents were delivered at 2 kHz without any frequency shifts, leading to a flat envelope of $tTIS_{control}$.[35]

7. Observation items and Observation methods

Visit Number[1)]	VISIT 1	VISIT 2	VISIT 3	VISIT 4	VISIT 5
	Screening and consenting	Baseline evaluation	Cerebellar TMS + Striatal tTIS + Cognitive task	Cerebellar TMS + Striatal tTIS + Cognitive task	Cerebellar TMS + Striatal tTIS + Cognitive task
Visit window				+ 3 days minimum	+ 3 days minimum
Clinical trial explanation and informed consent	●				
Eligibility determination (inclusion /exclusion criteria)	●				
Demographic data	●				
History taking	●				
Concomitant therapy	●				
Structural MRI<br>Functional MRI[3)]	●		(●)	(●)	(●)
Neuropsychological tests[2)]		●			
Task familiarization		●			
aMT calculation		●			
Randomized allocation		●			
Stimulation session : (TMS_{cb}/ $TMS_{control}$) + ($tTIS_{str}$ $tTIS_{control}$) + Cognitive task			●	●	●
Primary outcome			●	●	●
Secondary outcome			●	●	●

1) Screening and Baseline evaluation can be conducted on the same day based on patient's availability.
2) Neuropsychological evaluation include the tests detailed in the section Baseline neuropsychological evaluation.
3) For the MCI-LB cohort, a resting state functional MRI was performed before and after every stimulation session.

8.1 Cognitive task

The computerized working memory (WM) task was adapted from Harrington *et al.*[28] and included three different conditions (i.e. low-load, low-load with distraction, high-load). Each trial began with a cue (2 sec) indicating the shape category (squares or rectangles) to be remembered. In the low-load and high-load conditions, the cue consisted of a single filled white square or rectangle. In the low-load condition with a distraction, both a rectangle and a square were presented as cues. The filled shape indicated the target to be remembered, while the empty shape signified the distractors to be ignored. In the high-load condition, the cue remained the same, but the array contained six shapes to be remembered. After a jittered delay of 2,000-2,900 ms, an array of shapes was shown for 2 sec for encoding. After each array, a jittered delay (2,000-3,300 ms) was followed by a 2 sec probe display, presenting a single shape. Participants responded whether the probe matched any target from the preceding array in both color and spatial location. The time between trials (from the end of the probe to the start of the next cue) varied between 4 and 5,3 sec. Trials were pseudo-randomly distributed across five blocks of 28 trials each: one pre-stimulation, three during tTIS, and one post-stimulation block. Each block comprised 12 high-load, 12 distraction, and four low-load trials, totaling 140 trials

per session. Each block included an equal number of square and rectangle probes, as well as an equal number of matching and non-matching trials (50% each). In non-matching trials, the probe differed in either color or position.

8. Predicted Side Effects and Precautions during use

The potential side effects that could occur during this clinical trial include:

9.1 Intermittent theta burst cerebellar transcranial magnetic stimulation (TMS)

In a recent systematic review of original research studies published between September 2008 and November 2019 on cerebellar theta burst stimulation, 61 research articles investigated interventions of TBS to the cerebellum.[38] A total of 3176 active sessions of cerebellar TBS were reported in 1203 healthy participants and patients with various neurologic conditions, including brain injuries. No serious adverse events were reported. In 48 studies of this sample, the risk of adverse events was 4.1%. Adverse events consisted mostly of: 1) discomfort attributable to involuntary muscle contractions, 2) dizziness, 3) stress associated with the stimulation, 4) nausea, 5) mild headache, and 6) local discomfort.

In our study, no participants reported any significant adverse event, notably none among the ones mentioned above.

9.2 Transcranial Temporal Interference stimulation (tTIS)

TTIS has been reported to cause potential side effects similarly to low-intensity transcranial electrical stimulation techniques. TTIS might produce: 1) a tingling sensation at the stimulation site particularly at the beginning of stimulation, 2) itching, 3) feeling of warmth, 4) local discomfort, and 5) light flashes during the stimulation. In rare cases, a temporary redness below the electrodes, a feeling of nausea, dizziness, or mild headache has been reported.

In our study, during the evaluation of perceived tTIS sensations, prior to stimulation at the experimental stimulation intensity (2mA), nearly half of the patients and participants reported no sensation during $tTIS_{control}$ or $tTIS_{str}$ in the titration phase. Only 6.7% reported a strong sensation during $tTIS_{control}$ and 4.4% during $tTIS_{str}$. Among individuals who did report a sensation, tingling was the most frequently described. These findings are comparable with current literature.[35,36,39,40]

9.3 MRI

Magnetic resonance imaging (MRI) is a non-hazardous imaging technique, which can illustrate brain structure and function. It does not use radioactive material and is not resulting in radiation exposure. Because of the spatial confinement, in rare cases, subjects with claustrophobia could experience panic. Furthermore, due to the scanning, loud noises will occur. Caution is advised with persons suffering from severe heart diseases or with metal implants.

In our study, we inquired during the screening about the presence of claustrophobia. For loud noises, we employed noise-cancelling ear plugs during the scanning session. We also ran safety questionnaires to determine the presence of MRI-incompatible material, and we required patients who did not reach menopause to check for their pregnancy status.

9. Outcome Measure, Statistical Analysis, and Interpretation

Accuracy: was calculated as the number of correct answers divided by the sum of correct and incorrect responses, not taking into consideration the omission trials (which accounted for a total of 1.70% of all trials).

Mean Reaction Time (RTs): was calculated as the time interval from the presentation of the probe to the choice made during the task (match vs no match). RTs were calculated only for correct responses.

Resting-state fMRI connectivity: Pre-post rs-fMRI analysis compared baseline pre-stimulation Z-values using a separate LMM with stimulation condition as a fixed factor and participant as a random intercept. Associations between (i) connectivity changes (Z-values), (ii) pre-stimulation connectivity and stimulation-induced normalized behavioural performance changes were assessed using Pearson correlations with FDR correction for multiple comparisons. Specifically, we analysed differences in Accuracy and RTs across stimulation (Active control, $TMS_{control}$+$tTIS_{str}$, and TMS_{cb}+$tTIS_{str}$) and task conditions (high-load vs. distraction condition) in which our intervention showed some behavioural changes.

11. Statistical analyses of the outcomes

11.1 Primary outcomes

We used linear mixed-effects models (LMMs) to examine the main effects of stimulation condition and block of online Accuracy and RTs. Significant effects and trends were followed by post-hoc comparisons with Tukey's correction and effect sizes calculation. In parallel, we fitted Bayesian mixed-effects models to assess the strength of evidence for the respective effects of the stimulation condition.

11.2 Secondary outcomes

We used analogous LMMs with post-hoc comparisons where relevant. We examined associations between connectivity, brain structure, and behavioural changes using correlation and robust regression analyses.

12. References

1. McKeith IG, Ferman TJ, Thomas AJ, et al. Research criteria for the diagnosis of prodromal dementia with Lewy bodies. *Neurology*. 2020;94(17):743-755. doi:10.1212/WNL.0000000000009323

2. Albert MS, DeKosky ST, Dickson D, et al. The diagnosis of mild cognitive impairment due to Alzheimer's disease: Recommendations from the National Institute on Aging−Alzheimer's Association workgroups on diagnostic guidelines for Alzheimer's disease. *Alzheimers Dement*. 2011;7(3):270-279. doi:10.1016/j.jalz.2011.03.008

3. Verdon V, Candal-Zurcher A. Appréciation du degré de gravité en neuropsychologie et estimation de la capacité de travail. 2021.

4. Wessel MJ, Beanato E, Popa T, et al. Noninvasive theta-burst stimulation of the human striatum enhances striatal activity and motor skill learning. *Nat Neurosci*. 2023;26(11):2005-2016. doi:10.1038/s41593-023-01457-7

5. Sobczak-Edmans M, Lo YC, Hsu YC, et al. Cerebro-Cerebellar Pathways for Verbal Working Memory. *Front Hum Neurosci*. 2019;12:530. doi:10.3389/fnhum.2018.00530

6. Viñas-Guasch N, Ng THB, Heng JG, et al. Cerebellar Transcranial Magnetic Stimulation (TMS) Impairs Visual Working Memory. *The Cerebellum*. 2022;22(3):332-347. doi:10.1007/s12311-022-01396-2

7. Huang YZ, Edwards MJ, Rounis E, Bhatia KP, Rothwell JC. Theta Burst Stimulation of the Human Motor Cortex. *Neuron*. 2005;45(2):201-206. doi:10.1016/j.neuron.2004.12.033

8. Nasreddine ZS, Phillips NA, Bédirian V, et al. The Montreal Cognitive Assessment, MoCA: A Brief Screening Tool For Mild Cognitive Impairment. *J Am Geriatr Soc*. 2005;53(4):695-699. doi:10.1111/j.1532-5415.2005.53221.x

9. Movement Disorder Society Task Force on Rating Scales for Parkinson's Disease. The Unified Parkinson's Disease Rating Scale (UPDRS): Status and recommendations. *Mov Disord*. 2003;18(7):738-750. doi:10.1002/mds.10473

10. Ferman TJ, Smith GE, Boeve BF, et al. DLB fluctuations: Specific features that reliably differentiate DLB from AD and normal aging. *Neurology*. 2004;62(2):181-187. doi:10.1212/WNL.62.2.181

11. Yesavage JA, Brink TL, Rose TL, et al. Development and validation of a geriatric depression screening scale: A preliminary report. *J Psychiatr Res*. 1982;17(1):37-49. doi:10.1016/0022-3956(82)90033-4

12. Stiasny−Kolster K, Mayer G, Schäfer S, Möller JC, Heinzel−Gutenbrunner M, Oertel WH. The REM sleep behavior disorder screening questionnaire—A new diagnostic instrument. *Mov Disord*. 2007;22(16):2386-2393. doi:10.1002/mds.21740

13. Cummings JL, Mega M, Gray K, Rosenberg-Thompson S, Carusi DA, Gornbein J. The Neuropsychiatric Inventory: Comprehensive assessment of psychopathology in dementia. *Neurology*. 1994;44(12):2308-2308. doi:10.1212/WNL.44.12.2308

14. Johns MW. A New Method for Measuring Daytime Sleepiness: The Epworth Sleepiness Scale. *Sleep*. 1991;14(6):540-545. doi:10.1093/sleep/14.6.540

15. Benedict RHB, Schretlen D, Groninger L, Dobraski M, Shpritz B. Revision of the Brief Visuospatial Memory Test: Studies of normal performance, reliability, and validity. *Psychol Assess*. 1996;8(2):145-153. doi:10.1037/1040-3590.8.2.145

16. Bezdicek O, Libon DJ, Stepankova H, et al. Development, Validity, and Normative Data Study for the 12-Word Philadelphia Verbal Learning Test [czP(r)VLT-12] Among Older and Very Old Czech Adults. *Clin Neuropsychol*. 2014;28(7):1162-1181. doi:10.1080/13854046.2014.952666

17. Wechsler D. Wechsler Adult Intelligence Scale--Third Edition. Published online February 11, 2019. doi:10.1037/t49755-000

18. Nikolai T, Štěpánková H, Michalec J, et al. Tests of Verbal Fluency, Czech Normative Study in Older Patients. *Čes Slov Neurol Neurochir*. 2015;78/111(3):292-299. doi:10.14735/amcsnn2015292

19. Woodard JL, Benedict RHB, Salthouse TA, Toth JP, Zgaljardic DJ, Hancock HE. Normative Data for Equivalent, Parallel Forms of the Judgment ofLine Orientation Test. *J Clin Exp Neuropsychol*. 1998;20(4):457-462. doi:10.1076/jcen.20.4.457.1470

20. Benedict RHB, Schretlen D, Groninger L, Brandt J. Hopkins Verbal Learning Test – Revised: Normative Data and Analysis of Inter-Form and Test-Retest Reliability. *Clin Neuropsychol*. 1998;12(1):43-55. doi:10.1076/clin.12.1.43.1726

21. Sudarshan NJ, Bowden SC. Common Factor Structure of the Ten Subtest Wechsler Adult Intelligence Scale-Fourth Edition in a Clinical Sample and 15 Subtest Version in the Standardization Sample. *Arch Clin Neuropsychol Off J Natl Acad Neuropsychol*. 2023;38(8):1646-1658. doi:10.1093/arclin/acad035

22. St-Hilaire A, Hudon C, Vallet GT, et al. Normative data for phonemic and semantic verbal fluency test in the adult French–Quebec population and validation study in Alzheimer's disease and depression. *Clin Neuropsychol*. 2016;30(7):1126-1150. doi:10.1080/13854046.2016.1195014

23. Gaudreau AS, Macoir J, Hudon C. Normative data for the Color Trails Test in middle-aged and elderly Quebec-French people. *Appl Neuropsychol Adult*. 2025;32(1):116-124. doi:10.1080/23279095.2022.2156291

24. Tremblay MP, Potvin O, Callahan BL, et al. Normative Data for the Rey-Osterrieth and the Taylor Complex Figure Tests in Quebec-French People. *Arch Clin Neuropsychol*. 2015;30(1):78-87. doi:10.1093/arclin/acu069

25. Bradshaw JL, Nettleton NC, Nathan G, Wilson L. Head and body space to left and right, front and rear—II. Visuotactual and kinesthetic studies and left-side underestimation. *Neuropsychologia*. 1983;21(5):475-486. doi:10.1016/0028-3932(83)90004-0

26. Buysse DJ, Reynolds CF, Monk TH, Berman SR, Kupfer DJ. The Pittsburgh sleep quality index: A new instrument for psychiatric practice and research. *Psychiatry Res*. 1989;28(2):193-213. doi:10.1016/0165-1781(89)90047-4

27. Pfeffer RI, Kurosaki TT, Harrah CH, Chance JM, Filos S. Measurement of Functional Activities in Older Adults in the Community. *J Gerontol*. 1982;37(3):323-329. doi:10.1093/geronj/37.3.323

28. Harrington DL, Shen Q, Vincent Filoteo J, et al. Abnormal distraction and load-specific connectivity during working memory in cognitively normal Parkinson's disease. *Hum Brain Mapp*. 2020;41(5):1195-1211. doi:10.1002/hbm.24868

29. Noe E, Marder K, Bell KL, Jacobs DM, Manly JJ, Stern Y. Comparison of dementia with Lewy bodies to Alzheimer's disease and Parkinson's disease with dementia. *Mov Disord*. 2004;19(1):60-67. doi:10.1002/mds.10633

30. Voytek B, Knight RT. Prefrontal cortex and basal ganglia contributions to visual working memory. *Proc Natl Acad Sci U S A*. 2010;107(42):18167-18172. doi:10.1073/pnas.1007277107

31. Steiger TK, Herweg NA, Menz MM, Bunzeck N. Working memory performance in the elderly relates to theta-alpha oscillations and is predicted by parahippocampal and striatal integrity. *Sci Rep*. 2019;9(1):706. doi:10.1038/s41598-018-36793-3

32. Brissenden JA, Tobyne SM, Halko MA, Somers DC. Stimulus-Specific Visual Working Memory Representations in Human Cerebellar Lobule VIIb/VIIIa. *J Neurosci*. 2021;41(5):1033-1045. doi:10.1523/JNEUROSCI.1253-20.2020

33. Liang KJ, Carlson ES. Resistance, vulnerability and resilience: A review of the cognitive cerebellum in aging and neurodegenerative diseases. *Neurobiol Learn Mem*. 2020;170:106981. doi:10.1016/j.nlm.2019.01.004

34. Milardi D, Arrigo A, Anastasi G, et al. Extensive Direct Subcortical Cerebellum-Basal Ganglia Connections in Human Brain as Revealed by Constrained Spherical Deconvolution Tractography. *Front Neuroanat*. 2016;10. doi:10.3389/fnana.2016.00029

35. Wessel MJ, Beanato E, Popa T, et al. Noninvasive theta-burst stimulation of the human striatum enhances striatal activity and motor skill learning. *Nat Neurosci*. 2023;26(11):2005-2016. doi:10.1038/s41593-023-01457-7

36. Vassiliadis P, Beanato E, Popa T, et al. Non-invasive stimulation of the human striatum disrupts reinforcement learning of motor skills. *Nat Hum Behav*. 2024;8(8):1581-1598. doi:10.1038/s41562-024-01901-z

37. Beanato E, Moon HJ, Windel F, et al. Noninvasive modulation of the hippocampal-entorhinal complex during spatial navigation in humans. *Sci Adv*. 2024;10(44):eado4103. doi:10.1126/sciadv.ado4103

38. Hurtado-Puerto AM, Nestor K, Eldaief M, Camprodon JA. Safety Considerations for Cerebellar Theta Burst Stimulation. *Clin Ther*. 2020;42(7):1169-1190.e1. doi:10.1016/j.clinthera.2020.06.001

39. Vassiliadis P, Stiennon E, Windel F, Wessel MJ, Beanato E, Hummel FC. Safety, tolerability and blinding efficiency of non-invasive deep transcranial temporal interference stimulation: first experience from more than 250 sessions. *J Neural Eng*. 2024;21(2):024001. doi:10.1088/1741-2552/ad2d32

40. Piao Y, Ma R, Weng Y, et al. Safety Evaluation of Employing Temporal Interference Transcranial Alternating Current Stimulation in Human Studies. *Brain Sci*. 2022;12(9):1194. doi:10.3390/brainsci12091194
