# Supplementary material for: Multifocal Noninvasive Deep Brain Stimulation to Enhance Cognition in Mild Cognitive Impairment: A Crossover Trial
Source: JAMA Netw Open. 2026 Jul 6;9(7):e2621756. doi: 10.1001/jamanetworkopen.2026.21756 (PMC13338810; doi:10.1001/jamanetworkopen.2026.21756)
Supplement: Supplement 2. — eMethods. eTable 1. Measures of core clinical features of DLB in the MCI-LB group eTable 2. Molecular biomarkers for aMCI cohort eTable 3. Neuropsychological testing batteries used during baseline assessment of MCI patients and HC eTable 4. Neuropsychological comparisons eFigure 1. Radiological visual rating scales across MCI cohorts eFigure 2. Overview of Study Design, Cognitive Task, Stimulation Protocols, and Modeled Striatal E-Field Distribution eFigure 3. iTBS waveform pattern used during tTIS eFigure 4. Baseline and normalized behavioural performance across MCI cohorts eTable 5. Baseline (Pre-stimulation) Accuracy Across Cohorts and Conditions eTable 6. Baseline (Pre-stimulation) RTs Across Cohorts and Conditions eFigure 5. MCI patient clustering based on baseline performance eFigure 6. ICA component representing basal ganglia network eResults. eTable 7. Normalized Distraction Accuracy Across Blocks and Stimulation Conditions eTable 8. Normalized High-Load Accuracy Across Blocks and Stimulation Conditions eTable 9. Normalized Distraction RT Across Blocks and Stimulation Conditions eTable 10. Normalized High-Load RT Across Blocks and Stimulation Conditions eFigure 7. Posterior predictive checks for the Bayesian model assessing stimulation effects on Accuracy during the distraction condition in the MCI-LB Cohort eFigure 8. Posterior predictive checks for the Bayesian model assessing stimulation effects on RTs during the distraction condition in the aMCI Cohort eFigure 9. Stimulation effects on visuospatial WM performance in HC cohort (n = 20) eFigure 10. Effects of stimulation on attention, fatigue, sleep, decision-making, and emotion in the EPFL MCI cohort eFigure 11. Effects of stimulation on attention, fatigue, sleep, decision-making, and emotion in the EPFL HC cohort eFigure 12. Sensations reported for tTIS by EPFL participants eFigure 13. Evaluation of perceived tTIS sensations and blinding efficacy to tTIS stimulation condition for EPFL participants eFigur [file jamanetwopen-e2621756-s002.pdf]

Supplemental Online Content

Nencha U, Pupíková M, di Natale M, et al. Multifocal noninvasive deep brain stimulation to enhance cognition in mild cognitive impairment: a crossover trial. *JAMA Netw Open*. 2026;9(7):e2621756. doi:10.1001/jamanetworkopen.2026.21756

eMethods.
eTable 1. Measures of core clinical features of DLB in the MCI-LB group
eTable 2. Molecular biomarkers for aMCI cohort
eTable 3. Neuropsychological testing batteries used during baseline assessment of MCI patients and HC
eTable 4. Neuropsychological comparisons
eFigure 1. Radiological visual rating scales across MCI cohorts
eFigure 2. Overview of Study Design, Cognitive Task, Stimulation Protocols, and Modeled Striatal E-Field Distribution
eFigure 3. iTBS waveform pattern used during tTIS
eFigure 4. Baseline and normalized behavioural performance across MCI cohorts
eTable 5. Baseline (Pre-stimulation) Accuracy Across Cohorts and Conditions
eTable 6. Baseline (Pre-stimulation) RTs Across Cohorts and Conditions
eFigure 5. MCI patient clustering based on baseline performance
eFigure 6. ICA component representing basal ganglia network
eResults.
eTable 7. Normalized Distraction Accuracy Across Blocks and Stimulation Conditions
eTable 8. Normalized High-Load Accuracy Across Blocks and Stimulation Conditions
eTable 9. Normalized Distraction RT Across Blocks and Stimulation Conditions
eTable 10. Normalized High-Load RT Across Blocks and Stimulation Conditions
eFigure 7. Posterior predictive checks for the Bayesian model assessing stimulation effects on Accuracy during the distraction condition in the MCI-LB Cohort
eFigure 8. Posterior predictive checks for the Bayesian model assessing stimulation effects on RTs during the distraction condition in the aMCI Cohort
eFigure 9. Stimulation effects on visuospatial WM performance in HC cohort (n = 20)
eFigure 10. Effects of stimulation on attention, fatigue, sleep, decision-making, and emotion in the EPFL MCI cohort
eFigure 11. Effects of stimulation on attention, fatigue, sleep, decision-making, and emotion in the EPFL HC cohort
eFigure 12. Sensations reported for tTIS by EPFL participants

© 2026 Nencha U et al. *JAMA Network Open*.

eFigure 13. Evaluation of perceived tTIS sensations and blinding efficacy to tTIS stimulation condition for EPFL participants
eFigure 14. Associations between pre-stimulation putaminal connectivity and stimulation-induced behavioural changes in the MCI-LB group
eTable 11. Correlations between pre-stimulation basal ganglia connectivity and stimulation-induced changes in Accuracy under distraction in the MCI-LB group
eFigure 15. Associations between cerebellar volume and behavioural performance in the aMCI group
eFigure 16. Stimulation effects on visuospatial WM performance across patient clusters
eFigure 17. Group differences across clusters in baseline neuropsychological performance (a) and demographic variables and regional brain volumes (b)
eReferences

This supplemental material has been provided by the authors to give readers additional information about their work.

© 2026 Nencha U et al. *JAMA Network Open*.

eMethods

1. Patient allocation to stimulation order and inclusion assessments

1.1. Determining sample size

A power analysis based on prior tTIS research targeting the striatum (d = 0.76)[1] adjusted for an additional stimulation condition ($\alpha = 0.02$, $\beta = 0.8$), indicated that 22 participants per group would be required, which we closely matched while considering dropouts.

1.2. Full recruitment details

For the clinical population, from August 2023 to October 2024 participants were clinically evaluated by neurologists and neuropsychologists at the Geneva Memory Center or the Sierre Memory Center (Switzerland) for the EPFL cohort and selected from a longitudinal "at risk of DLB" cohort described previously[2–4] in MUNI (Czech Republic) for the MUNI cohort. At EPFL, we screened 71 people and included 28 patients that had received a clinical diagnosis of amnestic MCI syndrome[5] (aMCI; 18 from the Geneva Memory Center, 10 from the Sierre Memory Center) with eight dropouts. 65% of aMCI patients had at least one positive AD biomarker (12 CSF, 2 amyloid-PET, 4 both, eTable 2). Six patients were treated with cholinesterase inhibitors (4 with donepezil, 2 with rivastigmine), one of them with concomitant memantine and two others with selective serotonin reuptake inhibitors (SSRI). At MUNI, we screened 47 "at risk of DLB" subjects who were clinically followed for 1 to 4 years and included 25 L-dopa and cholinesterase inhibitors naïve patients with probable or possible MCI-LB based on clinical research criteria[6], and MDS level II criteria[7], having four dropouts. In total, 41 MCI completed the study and were included in the final analyses. In the HC group, there was one dropout at MUNI (lack of time).

1.3. Inclusion assessments for the MCI-LB cohort

We selected MCI-LB participants from our longitudinal cohort of at-risk DLB subjects described previously.[2–4] Participants were recruited from the community through advertisements and comprehensive telephone screening questionnaires. This screening assessed subjective symptoms of MCI-LB, including subjective cognitive decline, along with at least one of the following: sleep disturbances (restless sleep, screaming or other noises during sleep, sudden limb movements), bradykinesia and rigidity (slowed movements and muscle stiffness, reduced arm swings, decreased voice loudness and melody of speech), fluctuating memory and attention, visual hallucinations or misperceptions. We also recorded additional symptoms such as reduced olfaction, excessive daytime sleepiness, chronic constipation, symptomatic hypotension (without taking hypertension medication), and mood disorders. All symptoms had to have occurred within the past two months. Following the phone interview, participants underwent in-person clinical and neuropsychological examinations, up to one year prior to their inclusion in the present study. Core clinical features of Dementia with Lewy Bodies (DLB), that is parkinsonism, visual hallucinations, cognitive fluctuations and REM sleep behavior disorder, were assessed using the following standardized instruments: Montreal Cognitive Assessment (MoCA) with a cut-off score < 26 or ≤ 26 depending on years of education[8], Unified Parkinson's Disease Rating Scale Motor Examination (UPDRS III) with a cut-off score > 3 points[9], Mayo Fluctuation Scale (MFS) with a cut-off score ≥ 3 points[10], Geriatric Depression Scale (GDS) with a cut-off score of 10 points[11], REM sleep behavior disorder screening questionnaire (RBDSQ) with a cut-off score ≥ 5 points[12], Neuropsychiatric Inventory (NPI) for detecting hallucinations, misperceptions, or psychosis (Y/N)[13], and Epworth Sleepiness Scale (ESS) with a cut-off score of 11 points.[14] Neuropsychological battery for evaluation of Movement Disorder Society level II criteria for MCI in Parkinson's disease patients (PD-MCI) was also conducted, with cutoff scores in at least two neuropsychological tests under 1 standard deviation (SD) below the age appropriate norms: memory (brief visuospatial memory test-revised[15] and the Philadelphia Verbal Learning Test[16]); attention (Wechsler Adult Intelligence Scale-III: Letter-Number Sequencing and Digit Symbol Substitution[17]); executive functions (semantic and phonemic verbal fluency[18] and the picture arrangement test[17]); and visuospatial functions (Judgment of Line Orientation[19]). Based on the number of core clinical features, the participants are classified as possible MCI-LB (one core clinical feature) and probable MCI-LB (two or more core clinical features).

© 2026 Nencha U et al. *JAMA Network Open*.

	UPDRS	MoCA	ESS	RBDSQ	GDS	MFS	NPI
Mean	4.78	24.33	7.78	3.83	8.28	0.83	-
SD	4.20	2.85	3.97	2.06	6.36	0.83	-
% abnormal	48%	52%	19%	33%	24%	5%	14%

eTable 1. Measures of core clinical features of DLB in the MCI-LB group.
UPDRS = Unified Parkinson's Disease Rating Scale, MoCA = Montreal Cognitive Assessment, ESS = Epworth Sleepiness Scale, RBDSQ = REM sleep behavior disorder screening questionnaire, GDS = Geriatric Depression Scale, MFS = Mayo Fluctuation Scale, NPI = Neuropsychiatric Inventory.

1.3.1. Examination of MCI-LB cohort by transcranial sonography (TCS)

The patients were examined in a supine position. The temporal window was used, the transducer was placed on the temple parallel to the orbitomeatal line. Based on recommendations published elsewhere[20] we investigated with 14-16cm image depth, dynamic range 45-55 dB and with center frequency of insonation 2,0-3,5 MHz. The examination of SN was performed from both sides, echogenicity was measured only in the ipsilateral SN with respect to the insonated side.[20] A butterfly-shaped hypoechoic structure of mesencephalon is visible in the plane of the midbrain, bordered by hyperechogenic basal cisterns.[21]According to the recommendations, planimetric measurement was used, the echogenic signal of the SN was manually encircled and then the echogenic area was automatically calculated by an ultrasound device.[20] The higher value of SN echogenicity was used when bilateral measurements were available. Our laboratory established cut-off values for TCS of SN were determined using the 90th and 75th percentiles. For the purpose of this study, we define an echogenic area of ≥ 0,2 cm2 as a clearly hyperechogenic, an echogenic area of 0,16 – 0,19 cm2 as a mildly hyperechogenic and an area of ≤ 0,15 cm2 as clearly negative. These values also accord with the literature.[20]

1.4. Inclusion assessments for the aMCI cohort

Participants were enrolled following a clinical diagnosis of MCI, defined by objective memory impairment with at least two memory test scores < −1 SD below age-adjusted norms, or with impairment in memory along with at least one additional cognitive domain. The patients were examined by experienced clinical neuropsychologists, and the MCI diagnosis was formulated on the basis of the Swiss Neuropsychological Association Criteria.[22] The patients were assessed in two memory centers in Geneva and Sion (Switzerland) and were subsequently referred for screening at EPFL sites.

Following inclusion in the present study, all the participants underwent a similar neuropsychological evaluation as the MCI-LB cohort at baseline. The neuropsychological testing battery evaluated different cognitive domains such as: global cognition (MoCA with a cut-off score < 26 or ≤ 26 depending on years of education[8]); visual memory (Brief Visuospatial Memory Test-revised[15]) and verbal memory (Hopkins Verbal Learning Test-revised[23]); executive functions (Wechsler Adult Intelligence Scale-IV[24]: Letter-Number Sequencing and Digit Symbol Substitution[25], semantic and phonemic verbal fluency[26], Color Trail Making Test A and B[27]); and visuospatial functions (Rey-Osterrieth Figure - Copy[28] and Line Bisection Test[29]). All participants also completed self-reported questionnaires assessing their sleep quality (Pittsburgh Sleep Quality Index, PSQI[30]) and level of independence (Functional Activities Questionnaire, FAQ[31]).

	**Aβ-42 (n = 15)**	**Aβ-40 (n = 13)**	**Aβ-42/Aβ-40 (n = 13)**	**tau (n = 15)**	**p-tau (n = 15)**	**amy-PET (n = 6)**
Mean ± SD	808.8 ± 279.67	16066.69 ± 4563.76	0.050 ± 0.02	668.4 ± 276.10	105.46 ± 52.60	-
% positive	46.67	-	92.31	73.33	80	100

eTable 2. Molecular biomarkers for aMCI cohort.
Thresholds for positivity: CSF: Aβ-42 <725 ng/l; Aβ-42/Aβ-40 >0.069; tau >400 ng/l; p-tau >56.5 ng/l; amy-PET SUVr > 0.6. Aβ-42 = amyloid-beta-42; Aβ-40 = amyloid-beta-40; p-tau = phospho-tau; amy-PET = amyloid Positron Emission Tomography.

© 2026 Nencha U et al. *JAMA Network Open*.

A summary of the demographics and neuropsychological performances across the two MCI cohorts and HC is reported in Supplementary Table 1. All references for neuropsychological tests administered in each site can be found in Supplementary Table 2. Participants received a stipend for taking part in the study.

1.5. Randomization and masking

The allocation sequence was independently generated by two individuals who were not involved in the study, data collection or analyses (one at EPFL, one at MUNI). The sequence was concealed from participants, care providers, investigators performing the stimulation, outcome assessors, and data analysts. Blinding was maintained until the primary outcomes were analysed.

1.6. Allocation of patients to specific stimulation order

Once included in the study, MCI patients from both EPFL and MUNI were allocated to a specific order of stimulation sessions, following a predefined pseudorandomized order by two persons who were not involved in the study (one in EPFL, one in MUNI). Patients were allocated to the following stimulation sequences:
Order A = T3 (Active control), T4 ($TMS_{control}$+$tTIS_{str}$), T5 (TMS_{cb}+$tTIS_{str}$). n = 9 (4 EPFL, 5 MUNI);
Order B = T3 ($TMS_{control}$+$tTIS_{str}$), T4 (Active control), T5 (TMS_{cb}+$tTIS_{str}$). n = 9 (4 EPFL, 5 MUNI);
Order C = T3 (TMS_{cb}+$tTIS_{str}$), T4 (Active control), T5 ($TMS_{control}$+$tTIS_{str}$). n = 10 (4 EPFL, 5 MUNI);
Order D = T3 (Active control), T4 (TMS_{cb}+$tTIS_{str}$), T5 ($TMS_{control}$+$tTIS_{str}$). n = 6 (4 EPFL, 2 MUNI);
Order E = T3 ($TMS_{control}$+$tTIS_{str}$), T4 (TMS_{cb}+$tTIS_{str}$), T5 (Active control). n =11 (7 EPFL, 4 MUNI);
Order F = T3 (TMS_{cb}+$tTIS_{str}$), T4 ($TMS_{control}$+$tTIS_{str}$), T5 (Active control). n = 8 (5 EPFL, 3 MUNI).

There were two dropouts from the group allocated to Order A, three from the group allocated to Order B, one from the group allocated to Order C, one from the group allocated to Order D, three from the group allocated to Order E, two from the group allocated to Order F.

	MUNI		EPFL	
	**Test/Scale name (abbreviation)**	**reference for norms**	**Test/Scale name (abbreviation)**	**reference for norms**
Global cognition	Montreal Cognitive Assessment (MoCA)	Nikolai et al., 2018[32]	Montreal Cognitive Assessment (MoCA)	Larouche et al., 2016[33]
Visuospatial	Rey-Osterrieth Figure - Copy	Drozdova et al., 2014[34]	Rey-Osterrieth Figure - Copy	Tremblay et al., 2015[28]
	Judgement of Line orientation (JLO)	Woodard et al., 1998)[19]	Line Bisection Test	Bradshaw et al. 1983[29]
Visual memory	Brief Visuospatial Memory Test (BVMT)	Havlík et al., 2020[35]	Brief Visuospatial Memory Test (BVMT)	Benedict et al., 1996[15]
Verbal memory	Rey Auditory Verbal Learning test (RAVLT)	Bezdíček et al., 2014[16]	Hopkins Verbal Learning Test-revised (HVLT)	Benedict et al., 1998[23]
Executive functions	Trail Making Test: A and B (TMT-A, TMT-B)	Bezdíček et al., 2012[36]	Color Trail Making Test A and B (cTMT-A, cTMT-B)	Gaudreau et al., 2025[27]
	Wechsler Adult Intelligence Scale-IV: Digit Span	Nikolai et al., 2018[32]	Wechsler Adult Intelligence Scale-IV: Digit Span	Bowden et al., 2011[25]
	**MUNI**		**EPFL**	
	**Test/Scale name (abbreviation)**	**reference for norms**	**Test/Scale name (abbreviation)**	**reference for norms**
	Wechsler Adult Intelligence Scale-IV: Digit symbol substitution	Nikolai et al., 2018[37]	Wechsler Adult Intelligence Scale-IV: Digit symbol substitution	Bowden et al., 2011[25]
	Semantic Verbal Fluency (Animals)	Nikolai et al., 2015[18]	Semantic Verbal Fluency (Animals)	St-Hilaire et al., 2016[26]
	Phonetic Verbal Fluency (S-P-K)	Nikolai et al., 2015[18]	Phonetic Verbal Fluency (P)	St-Hilaire et al., 2016[26]
Scales	Functional Activities Questionnaire (FAQ)	Bezdíček et al., 2011[38]	Functional Activities Questionnaire (FAQ)	Pfeffer et al., 1982[31]
	Pittsburgh Sleep Quality Index (PSQI)	Manková et al., 2021[39]	Pittsburgh Sleep Quality Index (PSQI)	Buysse et al., 1989[30]

eTable 3. Neuropsychological testing batteries used during baseline assessment of MCI patients and HC.

© 2026 Nencha U et al. *JAMA Network Open*.

	Cohort				
	HC	MCI-LB	aMCI		
	n = 20	n = 21	n = 20	Statistic (df)	Adj. P-value
Neuropsychological Z-scores					
MoCA	0.32 ± 1.01	-0.10 ± 0.85	-1.52 ± 1.30[a,b]	$F_{(2, 58)}$= 16.41	p < 0.001
Visuospatial domain	0.33 ± 0.80	0.06 ± 0.95	-0.36 ± 1.14	$F_{(2, 58)}$ = 2.51	p = 0.09
Memory domain					
Visual Memory	0.48 ± 0.93	-0.55 ± 0.98[a]	-1.38 ± 0.96[a,b]	$F_{(2, 58)}$= 18.96	p < 0.001
Verbal Memory	0.33 ± 0.59	0.02 ± 0.95	-1.38 ± 1.15[a,b]	$F_{(2, 58)}$= 19.51	p < 0.001
Executive domain	0.20 ± 0.49	-0.36 ± 0.51[a]	-0.76 ± 0.63[a]	$F_{(2, 58)}$= 15.42	p < 0.001
FAQ	0.00 ± 0.00	0.00 ± 0.00	2.15 ± 2.50[a,b]	$F_{(2, 58)}$= 15.20	p < 0.001
PSQI	5.30 ± 2.72	7.82 ± 2.88[a]	5.40 ± 3.38[b]	$F_{(2, 58)}$= 4.03	p = 0.02

eTable 4. Neuropsychological comparisons.

Group comparisons were performed using one-way ANOVA. Post-hoc t-tests were Tukey corrected and p-values are presented in comparison-adjusted form. Post-hoc comparisons: [a] = significant difference from HC (adj. p < 0.05); [b] = significant difference from MCI-LB (adj. p < 0.05). MoCA = Montreal Cognitive Assessment, FAQ = Functional Activities Questionnaire, PSQI = Pittsburgh Sleep Quality Index.

© 2026 Nencha U et al. *JAMA Network Open*.

2. Visual Rating of White Matter Hyperintensities and Medial Temporal Atrophy

White matter hyperintensities (WMHs) were visually evaluated using the Fazekas scale, and medial temporal atrophy (MTA) was rated according to the Scheltens' MTA scale.[40,41] Both ratings were based on axial and coronal T1- and T2-weighted images. Trained assessors (AŠ, EV, DO, KŠ), blinded to participant identity and study condition, independently scored each patient. In cases of discrepancy, the final score was determined by consensus. The aMCI group tended to show higher MTA scores, reflecting greater medial temporal lobe atrophy, although this difference did not reach significance when compared to the MCI-LB group ($p = 0.07$, Supplementary eFigure 1). Conversely, the MCI-LB group showed a trend toward higher Fazekas scores, indicating greater white matter lesion burden, again without statistical significance ($p = 0.06$; Supplementary eFigure 1).

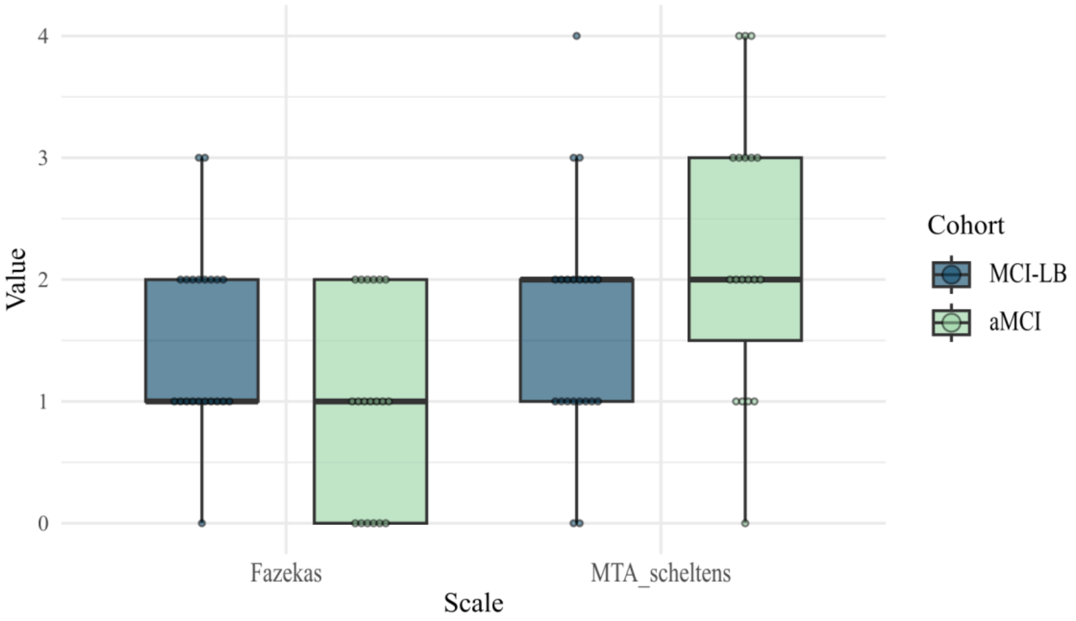

eFigure 1. Radiological visual rating scales across MCI cohorts.
Values represent semi-quantitative ratings of brain pathology severity. Higher values indicate greater pathology and 0 denotes no visible signs of pathology.

© 2026 Nencha U et al. *JAMA Network Open*.

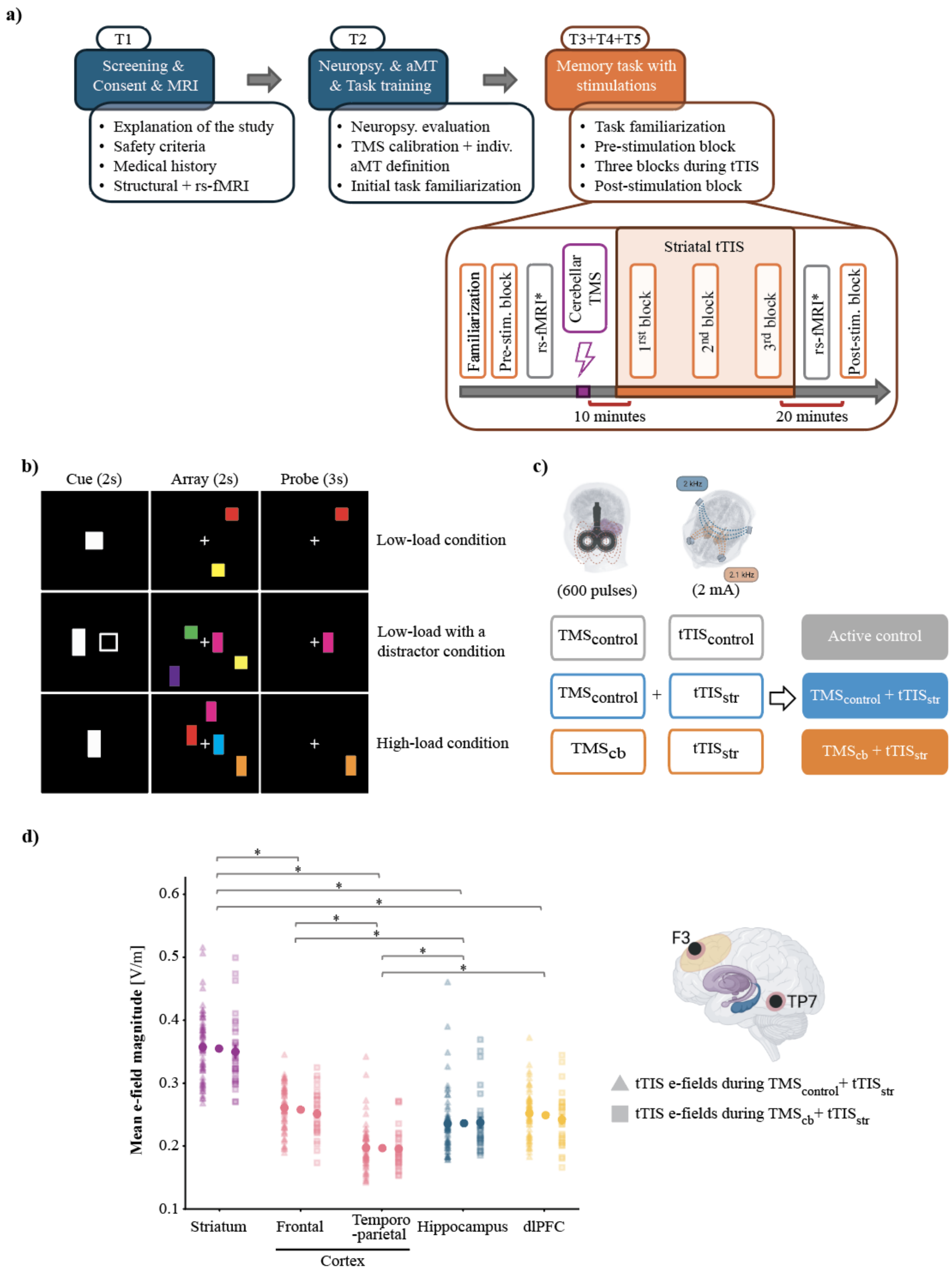

eFigure 2. Overview of Study Design, Cognitive Task, Stimulation Protocols, and Modeled Striatal E-Field Distribution.

a) Experimental protocol. Participants underwent baseline assessments (consent, MRI, neuropsychological testing, aMT), followed by task familiarization and three stimulation sessions. b) Visuospatial working memory (WM) task adapted from Harrington et al.[42], involving low-load, distractors and high-load trials. Participants responded to probes after encoding arrays of target (± distractor) shapes. For more details, see eMethods in

Supplement. c) Experimental stimulation conditions. Across sessions, subjects received one of three stimulation combinations in a pseudorandomized order: Active control (TMScontrol+tTIScontrol), active striatal tTIS with TMS active control (TMScontrol+tTISstr), or combined active cerebellar TMS and striatal tTIS (TMScb+tTISstr). d) Average electrical field (e-field) magnitude for bilateral striatum, across stimulation conditions. Details about the individualized head modeling and the e-field simulations are reported in eMethods in Supplement. For the full trial protocol see Supplement. rs-fMRI = resting-state functional MRI, rs-fMRI* = resting-state functional MRI performed only for MUNI cohort, neuropsy. evaluation = neuropsychological evaluation, indiv. aMT definition = individual active Motor Threshold definition, TMS = Transcranial Magnetic Stimulation, TMScontrol = TMS targeting the neck surface 5 cm below the cerebellar target, TMScb = TMS targeting the left inferior cerebellum, tTIS = transcranial Temporal Interference Stimulation, tTIScontrol = tTIS delivering high-frequency stimulation, tTISstr= tTIS delivering an intermittent theta-burst stimulation, Pre-stim. = pre-stimulation, Post-stim. = post-stimulation, dlPFC = dorsolateral prefrontal cortex, *$p < 0.05$.

3. Cognitive Task Timing and Trial Structure

The WM task was adapted from Harrington *et al.*[42] and included three different conditions (i.e. low-load, low-load with distraction, high-load). Each trial began with a cue (2 sec) indicating the shape category (squares or rectangles) to be remembered (Supplementary eFigure 2b). In the low-load condition, the cue consisted of a single filled white square or rectangle. In the low-load condition with a distraction, both a rectangle and a square were presented as cues. The filled shape indicated the target to be remembered, while the empty shape signified the distractors to be ignored. In the high-load condition, the cue remained the same, but the array contained six shapes to be remembered. After a jittered delay of 2,000-2,900 ms, an array of shapes was shown for 2 sec for encoding. After each array, a jittered delay (2,000-3,300 ms) was followed by a 2 sec probe display, presenting a single shape. Participants responded whether the probe matched any target from the preceding array in both color and spatial location. The time between trials (from the end of the probe to the start of the next cue) varied between 4 and 5,3 sec. Trials were pseudo-randomly distributed across five blocks of 28 trials each (Supplementary eFigure 2a): one pre-stimulation, three during tTIS, and one post-stimulation block. Each block comprised 12 high-load, 12 distraction, and four low-load trials, totaling 140 trials per session. Each block included an equal number of square and rectangle probes, as well as an equal number of matching and non-matching trials (50% each). In non-matching trials, the probe differed in either color or position.

4. Transcranial Magnetic Stimulation (TMS)

TMS was delivered with the MC-B70 coil connected to the Magventure MagPro X100 stimulator (MagVenture, Farum, Denmark) at EPFL and the Deymed DuoMAG XT stimulator (non-air cooled) at MUNI, using a frameless stereotactic neuro-navigation system: Localite at EPFL (Localite GmbH, Bonn, Germany) and BrainSight at MUNI (BrainSight Rogue Research Inc., Canada). The TMS coil was positioned vertically during iTBS delivery, with the handle pointing upwards targeting the left inferior cerebellum (TMS_{cb}) (MNI = -30 -74 -51). For the Active control condition ($TMS_{control}$), the same coil and stimulation parameters were used, but the coil was positioned 5 cm vertically downwards from the target and placed on the neck surface with the handle pointing upwards. This control condition and placement was chosen to mimic sound, vibration, and scalp sensation of TMS, without directly stimulating the cerebellar surface or its output[43–45], and we estimate that the half value depth ($S_{1/2}$) of our figure-of-eight coils to be as low as 5cm^2, as previously described in a comparative study.[46] In order to define personalized TMS intensities, single-pulse TMS was applied to the left $M1_{HAND}$ was applied to determine the resting and active motor thresholds (rMT and aMT) at the right first dorsal interosseous (FDI) muscle. The coil was positioned posterior to anterior with a 45 degrees angle towards the midsagittal line to determine the hotspot for activating the FDI muscle. aMT was defined as the lowest intensity of stimulation output that elicitings motor evoked potentials (MEPs) of ~200 µV in 50% of trials, while the right FDI muscle was contracted isometrically at 10-20% of maximum voluntary contraction. MEPs were recorded using Signal software (Cambridge Electronic Design, Cambridge, UK) at EPFL and integrated TruTrace EMG system integrated with the Deymed DuoMAG XT stimulator at MUNI.

TMS was applied using an iTBS protocol at 80% of each participant's aMT, targeting the left inferior cerebellum (MNI = -30 -74 -51) (TMS_{cb} based on previous neuroimaging[47] and TMS studies[48]). The stimulation target was calculated for each subject and projected as an entry point onto the scalp. Either iTBS over the cerebellum (TMS_{cb}) or iTBS over the neck as Active control ($TMS_{control}$) was delivered in three separate sessions, in a pseudo-randomized order, ending approximately 10 minutes before the beginning of the WMcognitive task with tTIS. The iTBS protocol consisted of delivering ten bursts, each made up of three pulses at 50Hz, repeated at 5Hz. This block was repeated twenty times every 10 sec to give a total of 600 pulses, as previously described.[49]

During the procedure, participants were seated in a comfortable chair and were instructed to stay awake and not to talk, move excessively, or use electronic devices. During the TMS protocol, the lead experimenter left the room to preserve blinding to the stimulation condition.

© 2026 Nencha U et al. *JAMA Network Open*.

5. Transcranial temporal Interference Stimulation (tTIS) waveform pattern and electric fields induced during stimulation

TTIS was delivered using two pairs of circular rubber electrodes ($3cm^2$ in surface) connected to two independent DS5 isolated bipolar constant current stimulators (Digitimer Ltd). The electrodes were placed on the skin according to the 10-10 EEG system: one anterior pair at F3-F4 and one posterior pair at TP7-TP8 positions, based on previous work.[1] Active tTIS stimulation delivered an intermittent theta-burst pattern ($tTIS_{str}$) and Active control condition delivered a high-frequency (HF) stimulation ($tTIS_{control}$), as previously described.[1,50] For details about the waveform pattern, see Supplementary eFigure 3. Prior to the main stimulation experiments, evaluation of perceived sensations was performed with the intensity per channel increasing step-wise, i.e. 0.5 > 1 > 1.5 > 2 mA, in line with recent works.[1,50,51] At the end of all three sessions, participants at EPFL were asked to report any sensations experienced during concurrent tTIS and task performance for each session, and to guess whether they had received $tTIS_{str}$ or $tTIS_{control}$ in each case. The application of tTIS was conducted in accordance with established safety, ethical, legal, regulatory, and application guidelines.[52]

Intermittent theta burst stimulation (iTBS) consisted of bursts of three pulses at 100 Hz, repeated every 200 ms (i.e., at a 5 Hz theta rhythm), for 2 sec (a single train). To generate this pattern using tTIS ($tTIS_{str}$), the first channel continuously delivered a 2 kHz current (f_1), while the second channel alternated between f_1=2 kHz and $f_1+\Delta f$=2.1 kHz every 200 ms. This alternation lasted 30 ms during each 2-sec train, producing the 100 Hz bursts. During the interburst and intertrain intervals (8 sec), non-amplitude-modulated HF stimulation was applied (Supplementary eFigure 3). For control tTIS, two oscillatory HF currents were delivered at 2 kHz without any frequency shifts, leading to a flat envelope of $tTIS_{control}$.[1] The stimulation waveforms were generated using a custom-based MATLAB graphical user interface and transmitted to the current sources using a standard digital-analogue converter (DAQ USB-6216, National Instruments - aMCI and HC in EPFL, HC in MUNI) or waveform generator (EDU33212A, Keysight - MCI-LB in MUNI). An audio transformer was added between stimulators and participants to avoid possible direct current accumulation. The maximal current intensity per stimulation channel was set to 2 mA (peak-to-baseline). The stimulation was delivered in three blocks, each lasting 7 min 21 sec, with 50 sec breaks between each of the three training blocks during each stimulation session. At EPFL, 5 sec ramp-up and ramp-down were delivered in-between each block. At MUNI, 30 sec ramp-up was delivered in the first block, with 10 sec ramp-up in the 2nd and 3rd blocks.

To confirm the accuracy of striatal targeting (Supplementary eFigure 2d), we simulated the individual induced electrical fields during tTIS. Individual head models were built using both T1 and T2-weighted MRI images, following the standard pipeline from SimNIBS[44] (i.e., using the *charm* command). Then, we simulated the electric field resulting from each electrode pair, using a quasi-static approximation, and calculating the maximum amplitude of the modulation envelope resulting from the interference of both fields.[53,54] The Brainnetome Atlas[55] was used to define the Regions of Interest (ROI) for each individual, which were then used to extract the average field magnitude for each ROI. For the EPFL participants, electrode locations were determined according to the coordinates recorded using the same neuro-navigation at the beginning of each experimental session. For MUNI participants, theoretical electrode locations were used based on the 10-10 EEG system and adjusted using individual fiducial points (Nasion, Inion, and bilateral preauricular points). We observed that the simulated electric fields induced by $tTIS_{str}$ were highest in the striatum (0.36 ± 0.06 V/m., Supplementary eFigure 2d), particularly relative to the cortical regions closest to the electrodes, superficial cortical areas (i.e. dlPFC), and other deep structures (i.e. hippocampus) (all $p < 0.0001$). In contrast, the weakest fields were observed in cortical regions near the temporo-parietal electrode pair, compared to frontal, dlPFC, and hippocampal regions (all $p < 0.0001$). Additionally, the cortex beneath the frontal electrodes showed a significantly lower electric field compared to hippocampus ($p < 0.01$).

© 2026 Nencha U et al. *JAMA Network Open*.

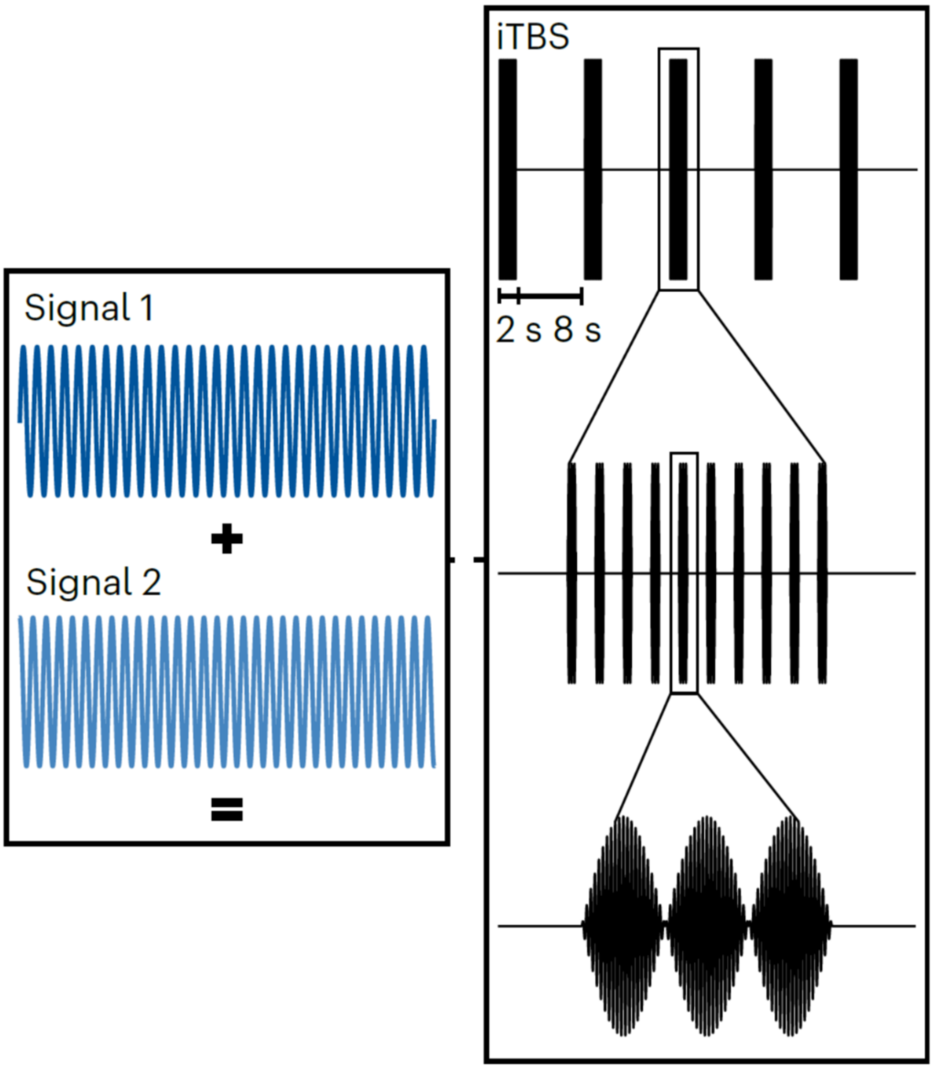

eFigure 3. iTBS waveform pattern used during tTIS.
Reproduced from Wessel et al., 2023[1] with permission.

6. **MRI Data Acquisition and Preprocessing**

The MRI data was acquired via 3.0T Magnetom Siemens Prisma at both centers. For the structural MRI data, the T1 MPRAGE sequence was used. A gradient-echo, T2 echo-planar imaging sequence was used for the resting state functional MRI (rs-fMRI).

7. **Magnetic resonance Imaging (MRI) sequence parameters**
   - Structural MRI sequence information: TR 2300 ms; TE 2.96 ms; voxel size 1 × 1 × 1 mm; FoV 256 × 256 mm; flip angle 9°; 192 transversal slices.
   - Functional MRI (fMRI) sequence information:
     - resting-state (rs-fMRI): TR 1250 ms; TE 32 ms; voxel size 2 × 2 × 2 mm; FoV 224 mm; flip angle 65°; 76 transversal slices; 480 scans; multiband factor 4. During the acquisition of rs-fMRI data, all subjects were instructed to stare at a white fixation cross (EPFL) / close their eyes (MUNI) on a blank screen and to try not to think about any specific subject while not falling asleep.

8. **Behavioral data preprocessing and exclusion criteria**

Low-load trials and omissions were excluded, and accuracy was calculated as the number of correct answers divided by the sum of correct and incorrect responses. RTs were calculated only for correct responses. Low-load trials were excluded as they were underrepresented and primarily intended to facilitate task engagement. Omissions were removed because they could reflect either technical issues (e.g., unregistered button presses) or attentional lapses, representing only 1.70% of all trials (>14,000 in total). One session of one subject was excluded from the main analysis due to a protocol violation, and another due to missing baseline data. All data were retained for the analyses, and no outlier removal procedures were applied.

© 2026 Nencha U et al. *JAMA Network Open*.

9. Baseline behavior and WM task performance across cohorts

At baseline, aMCI patients showed significantly lower Accuracy and higher RTs than HC and MCI-LB groups, while no differences were observed between HC and MCI-LB. Baseline measures were taken from the pre-stimulation block at the start of each session. Data were analysed using a linear mixed-effects model (LMM) with fixed effects of cohort (HC, MCI-LB, aMCI) and pre-session block (pre-Active control, pre-($TMS_{control}$+$tTIS_{str}$), pre-(TMS_{cb}+$tTIS_{str}$), followed by post-hoc comparisons with Tukey's correction. A significant main effect of cohort was found for both Accuracy ($p < 0.001$, $p\eta^2 = 0.26$) and RTs ($p < 0.001$, $p\eta^2 = 0.26$), whereas no significant effect of pre-session block was observed, reflecting the absence of carry-over effect of stimulation across the different sessions at baseline. Post-hoc comparisons revealed that the aMCI group (Accuracy: mean 67.5%, 95% CI 63.7%-71.2%; RTs: mean 1738ms, 95% CI 1620ms-1856ms) performed significantly worse than both the HC group (Accuracy: mean 78.8%, 95% CI 75.1%-82.5%; $p < 0.001$, $|d| = 1.38$; RTs: mean 1386ms, 95% CI 1268ms-1504ms; $p < 0.001$, $|d| = -2.09$) and the MCI-LB group (Accuracy: mean 76.1%, 95% CI 72.4%-79.7%; $p = 0.004$, $|d| = 1.05$; RTs: mean 1450ms, 95% CI 1334ms-1565ms; $p = 0.003$, $|d| = -1.71$), while no significant differences were observed between the HC and MCI-LB cohorts (Accuracy: $p = 0.56$, $|d| = 0.33$; RTs: $p = 0.72$, $|d| = -0.38$). Boxplots display group means with SD. For means and CI see eTable 5-6.

9.1. Baseline behavior and WM task performance across MCI cohorts

To ensure comparability across experimental conditions, we first evaluated baseline behavioral performance (Accuracy and RTs) across all pre-stimulation sessions within the merged MCI cohort (Supplementary eFigure 4a). There were no significant differences in either Accuracy ($p = 0.83$) or RTs ($p = 0.22$) at baseline across the pre-Active control, pre-tTIS ($TMS_{control}$+$tTIS_{str}$), pre-TMS+tTIS (TMS_{cb}+$tTIS_{str}$) sessions, with no evidence of carry-over effects.

We next compared baseline performance between the two MCI cohorts (i.e., aMCI and MCI-LB; Supplementary eFigure 4b). Linear mixed-effects model (LMM) analyses revealed that aMCI exhibited significantly lower Accuracy (aMCI: mean 67.5%, 95% CI 63.5%-71.4%; MCI-LB: mean 76.1%, 95% CI 72.2%-79.9%; $p = 0.003$, $p\eta^2 = 0.21$) and higher RTs (aMCI: mean 1738ms, 95% CI 1608ms-1868ms; MCI-LB: mean 1450ms, 95% CI 1323ms-1577ms; $p = 0.003$, $p\eta^2 = 0.21$) compared to MCI-LB. Furthermore, the pseudorandomization was successful, without carry-over effect between sessions.

To assess relative changes of the WM natural performance over time, we normalized block-wise scores (Accuracy and RTs) to each patient's baseline within the Active control condition (Supplementary eFigure 4c). After normalization, group differences were no longer significant (Accuracy: $p = 0.68$; RTs: $p = 0.41$) during the three training blocks. Additionally, no significant Accuracy improvement was observed across blocks ($p = 0.68$). However, RTs decreased across blocks, with a significant block x cohort interaction ($p = 0.02$, $p\eta^2 = 0.10$). In the aMCI group, RTs were significantly lower in the second and third blocks compared to the first (1^{rst} block: mean 1.06, 95% CI 1.01-1.10; 2nd block: mean 0.96, 95% CI 0.91-1.00; 3rd block: mean 0.98, 95% CI 0.93-1.03; $p < 0.001$, $d = 1.26$ and $p = 0.01$, $d = 0.96$, respectively). In the post-stimulation block, group differences in Accuracy were non-significant ($p = 0.85$), while RTs showed a significant cohort effect ($p = 0.01$, $p\eta^2 = 0.15$), driven by lower RTs for the MCI-LB cohort compared to the aMCI cohort (MCI-LB: mean 0.92, 95% CI 0.87-0.98; aMCI: mean 1.02, 95% CI 0.97-1.08; $p = 0.01$, $d = 0.82$).

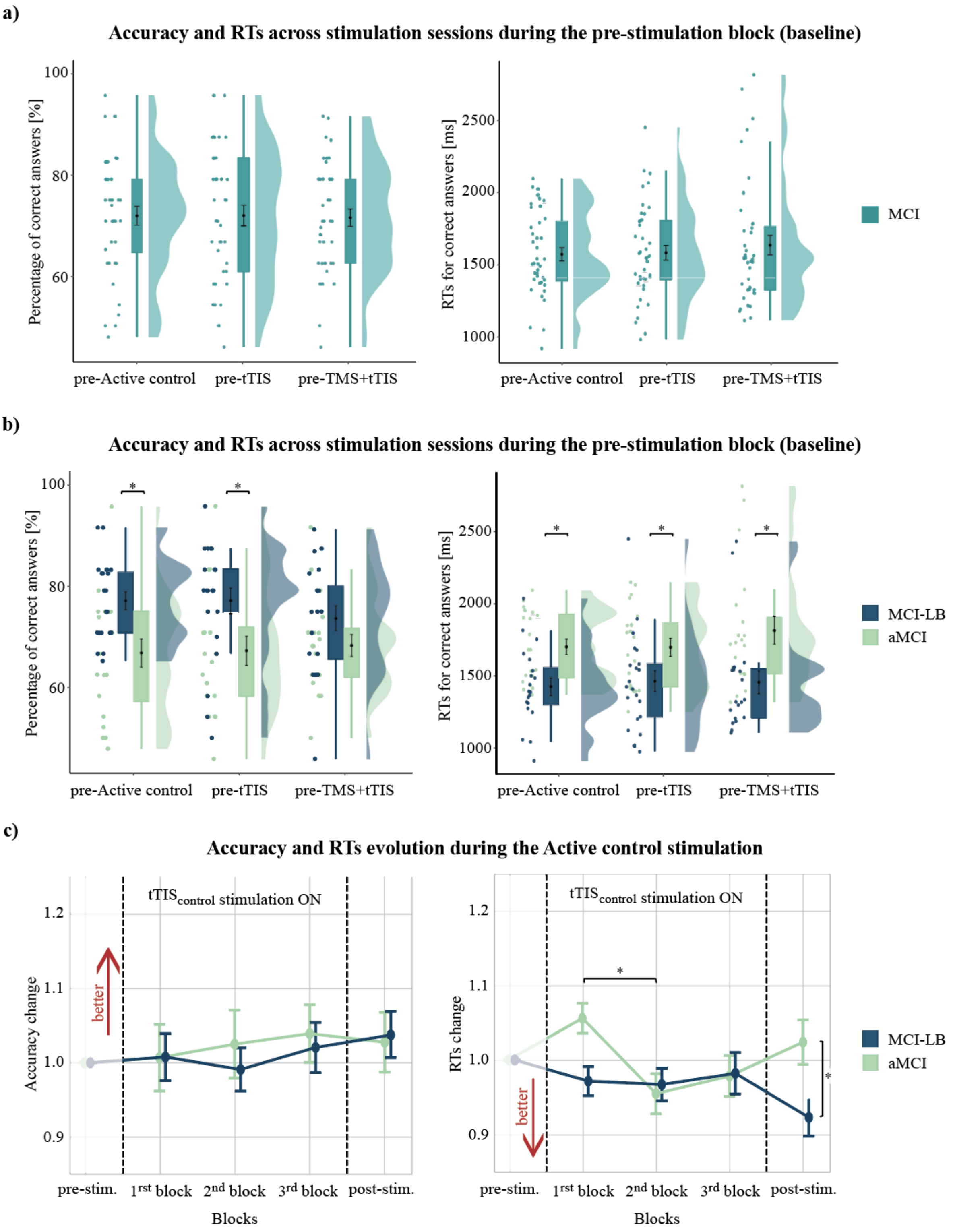

© 2026 Nencha U et al. *JAMA Network Open*.

eFigure 4. Baseline and normalized behavioural performance across MCI cohorts.
a) Baseline behavioural performance (Accuracy and RTs) at the start of each session was comparable across pre-Active control, pre-($TMS_{control}$+$tTIS_{str}$) and pre-(TMS_{cb}+$tTIS_{str}$) conditions in the merged MCI cohort, confirming the absence of session-order or carry-over effects. Boxplots display group means with SD.
b) At baseline, aMCI patients showed significantly lower Accuracy and higher RTs than MCI-LB cohort. Boxplots display group means with SD.
c) After normalization to baseline performance, no significant group differences were observed in the evolution of Accuracy or RTs during the Active control condition. A general decrease of RTs was observed across blocks, especially between the first and second blocks. Additionally, in the post-block MCI-LB patients had lower RTs compared to aMCI. Lines represent group means across blocks, with error bars indicating standard error of the mean (SEM).
*$p < 0.05$. pre-tTIS = pre-($TMS_{control}$+$tTIS_{str}$), pre-TMS+tTIS = pre-(TMS_{cb}+$tTIS_{str}$), Pre-stim. = pre-stimulation block, Post-stim. = post-stimulation block.

	**Pre-stimulation ACC (%)**					
	**Active control**		**$TMS_{control}$+$tTIS_{str}$**		**TMS_{cb}+$tTIS_{str}$**	
**Cohort**	**Mean**	**SD**	**Mean**	**SD**	**Mean**	**SD**
HC	77,71	10,14	78,75	11,62	79,87	6,84
n	20		20		20	
LB-MCI	77,11	7,96	77,14	11,63	73,67	11,14
n	20		21		20	
aMCI	66,82	12,41	67,25	12,74	68,29	9,87
n	20		20		20	

eTable 5. Baseline (Pre-stimulation) Accuracy Across Cohorts and Conditions

	**Pre-stimulation RT (ms)**					
	**Active control**		**$TMS_{control}$+$tTIS_{str}$**		**TMS_{cb}+$tTIS_{str}$**	
**Cohort**	**Mean**	**SD**	**Mean**	**SD**	**Mean**	**SD**
HC	1373,71 ±	237,61	1380,67 ±	235,02	1402,97 ±	242,70
n	20		20		20	
LB-MCI	1426,06 ±	268,96	1463,98 ±	336,92	1457,30 ±	357,93
n	20		21		20	
aMCI	1701,77 ±	243,88	1698,81 ±	277,04	1814,59 ±	425,50
n	20		20		20	

eTable 6. Baseline (Pre-stimulation) RTs Across Cohorts and Conditions

10. Statistical behavioral analysis methods

All statistical analyses were conducted in R (version 4.4.1, University of Auckland, https://www.R-project.org/). Baseline neuropsychological Z-scores were compared across cohorts using one-way ANOVA with cohort as main effect, followed by Tukey’s post-hoc correction. Assumptions of ANOVA were verified by testing normality (Shapiro-Wilk test) together with visual inspection of distribution and Q-Q plots, and homogeneity of variances (Levene’s test). Linear mixed-effects models (LMMs) were fitted using the *lmer()* function from the *lme4*

© 2026 Nencha U et al. *JAMA Network Open*.

package[61] with default setting for variance-covariance structure, significance testing and summary tables were computed using the *lmerTest* package.[62] For the analysis of tTIS online performance, block and stimulation conditions were included as fixed effects, with a random intercept for each participant. For the post-stimulation analysis, only stimulation conditions were included as a fixed effect, with participants again modeled as a random intercept. Significance testing was performed using the *anova()* function with Satterthwaite's approximation for degrees of freedom, implemented via the *lmerTest* package.[62] Post-hoc comparisons were conducted using estimated marginal means (EMMs) with Tukey's correction via the *emmeans* package.[63]
Residuals of the models were investigated to assess normality, skewness (between -2 and 2)[64] and homoskedasticity. One model (RTs during post-block for the MCI-LB during distraction condition) showed substantial positive skewness in the residuals. To address this, RTs were log-transformed, which improved residual normality, and the model was refitted with the transformed variable.
Effect sizes were calculated using the *effectsize* package[65], and reported as partial eta-squared ($p\eta^2$) for F-tests and Cohen's *d* (*d*) value for post hoc comparisons. Interpretation followed standard thresholds[66]:

- $p\eta^2$: < 0.01 (micro), 0.01 (small), 0.06 (medium), 0.14 (large)
- |d|: < 0.2 (micro), 0.2 (small), 0.5 (medium), 0.8 (large)

We fitted Bayesian generalized linear mixed-effects models using the *brm()* function from the *brms* package[67] for effects of stimulation conditions that were significant or showed a trend in the frequentist analysis. The models assessed the effects of stimulation condition and block on outcomes (Accuracy or RTs), including a random intercept for each participant.

- Model Specification
  Outcomes were modeled using a Gamma family with a log link function to account for positive and right-skewed distributions. Priors were specified as weakly informative: normal(0, 5) for population-level fixed effects, gamma(2, 0.1) for the Gamma shape parameter, and cauchy(0, 0.5) for the SD of the participant-level random intercepts.
- Sampling and Convergence
  Models were run with four Markov chains, each with 11,000 iterations and 1,000 warm up iterations. Convergence diagnostics included potential scale reduction factors (Rhat) and effective sample sizes (ESS) for bulk and tail distributions.[68]
- Model Diagnostics and Outputs
  Posterior summaries display mean estimates and 95% credible intervals (CIs) for each parameter. These CIs represent the range within which the parameter lies with 95% posterior probability, given the data and priors.[67]
- Model Checks and Validation
  Prior predictive checks were performed by fitting models with *sample_prior = "only"*. Bayes factors were calculated using the Savage-Dickey density ratio method implemented in bayesfactor_parameters().[69] Posterior predictive checks were conducted using the *bayesplot* package, comparing observed and predicted data distributions via density overlays, SD, quantiles, and empirical cumulative distribution functions (ECDF).[70]
- Model fit was evaluated using approximate leave-one-out cross-validation (LOO) with Pareto k diagnostics, expected log predictive density (elpd_loo), effective number of parameters (p_loo), and LOO information criterion (LOOIC).[71]

We set a fixed random seed (*seed = 123*) for all sampling procedures in the Bayesian models to guarantee reproducibility of posterior samples and results.

11. Clustering analysis

Previous studies have shown that effects of brain stimulation may vary as a function of the state of the neural system, i.e. the ability of an individual to integrate task-relevant information and the respective performance of the task.[71] Consequently, an additional analysis was conducted, in which MCI patients were regrouped based on their performance at baseline. The parameters used to characterize performance and to cluster the patients were, respectively, the speed and the Accuracy at the baseline block of the task (i.e., previous to any stimulation), on the first session (i.e., irrespective of the condition assigned for the first visit). The features extracted to capture the state of the neural system were the speed and the Accuracy in the high-load condition, and those same parameters relative to the speed and the Accuracy in the distractor condition (i.e., ratio of performance in high-load to distraction) to capture the difference between these two types of trials. These four features were used to identify clusters among the participants, using K-means clustering. The number of clusters was determined based on the score obtained using three criteria (i.e., Silhouette, Calinski-Harabasz, and Davies-Bouldin), with the maximum score obtained for K = 3, which resulted in three clusters being identified (Supplementary eFigure 5a). These three groups were labelled Cluster 0 (n = 17; eight aMCI and nine MCI-LB), Cluster 1 (n = 11; three aMCI and eight MCI-LB), and Cluster 2 (n = 13; nine aMCI and four MCI-LB).

© 2026 Nencha U et al. *JAMA Network Open*.

To visualize the distribution of individuals across the three clusters, a Radial Visualization (RadViz)[72] was employed (Supplementary eFigure 5b). In this plot, each feature is represented as an anchor point positioned on the perimeter of a circle. The proximity of an individual to a particular anchor indicates the relative dominance of that feature in their performance profile. Subsequently, we examined the baseline task performance of patients within each cluster (Supplementary eFigure 5c). Patients in Cluster 0 demonstrated stronger WM performance, as evidenced by higher Accuracy scores in the high-load condition. Cluster 1 included patients with intermediate WM performance, characterized by moderate Accuracy in high-load trials but relatively high Accuracy in the distraction condition. This cluster also showed the fastest RTs in both high-load and distraction trials. In contrast, Cluster 2 encompassed individuals with poorer WM performance, reflected by the lowest Accuracy in the high-load condition at baseline.

To investigate potential demographic and anatomical differences across the identified clusters, we compared participants' age, sex, years of education (YOE), neuropsychological profiles at baseline between groups, as well as regional brain volumes. For the anatomical analysis, gray matter volumes were extracted from specific ROIs including the hippocampus, caudate, putamen, striatum, and cerebellum, using the recon-all function in FreeSurfer (version 7.4.1 https://surfer.nmr.mgh.harvard.edu/) and normalized by the individual cortical total volume (CTV). All parameters (e.g., age, YOE, Neuropsychological Z-scores, normalized ROI volumes) were assessed for normality. Depending on distributional properties, between-group comparisons were performed using either a one-way analysis of variance (ANOVA) or the Kruskal–Wallis test. Sex distribution was compared using Chi-square test. When a significant effect of group was found, pairwise post-hoc comparisons were performed using Tukey correction for ANOVA and Bonferroni correction for Kruskal-Wallis. All post-hoc analyses were implemented using the *multcompare()* function in MATLAB for the anatomical analysis.

© 2026 Nencha U et al. *JAMA Network Open*.

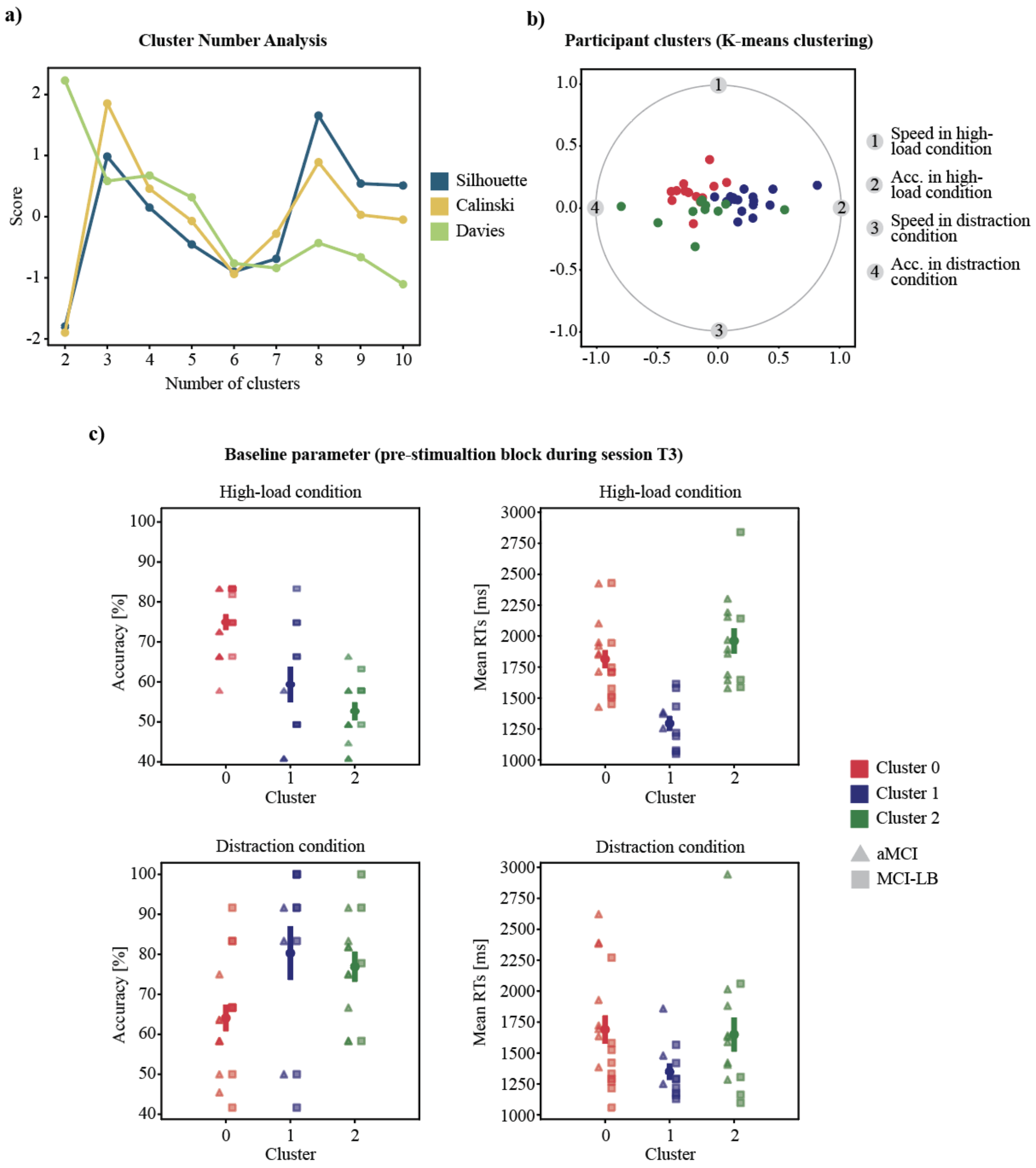

eFigure 5. MCI patient clustering based on baseline performance.

a) Cluster analysis using Silhouette, Calinski-Harabasz and Davies-Bouldin scores indicating that the optimal number of clusters was three.

b) Radial visualization of patient distribution across clusters. Each gray anchor point represents a key performance feature (speed and Accuracy in high-load and distraction conditions). The closer a subject's point is to one of these anchors, the more dominant that feature is in their profile.

c) Baseline performance profiles for the three clusters. Cluster 0 includes patients with stronger WM, as indicated by high accuracy in the high-load condition. Cluster 1 represents patients with moderate WM. These individuals show mid-level Accuracy in high-load trials but high Accuracy in distraction trials. This cluster also exhibits the fastest RTs in both high-load and distraction conditions. Cluster 2 includes patients with weaker WM, showing the lowest Accuracy in the high-load condition at baseline.

Acc. = Accuracy.

© 2026 Nencha U et al. *JAMA Network Open*.

12. rs-fMRI data analysis

Pre-processing pipeline and ICA analysis

The rs-fMRI data were preprocessed and analyzed with SPM12 (The Wellcome Department of Cognitive Neurology, http://fil.ion.ucl.ac.uk/spm/) in MATLAB R2019a (Mathworks). The data preprocessing pipeline steps included realignment and unwarping, spatial normalization, and spatial smoothing (6 mm FWHM). This was followed by nuisance regression using a general linear model (GLM), accounting for signals from white matter and cerebrospinal fluid, as well as 24 motion parameters (translations and rotations, their differences, their squares, and their squares of the differences). Finally, a high-pass filter with a cut-off frequency of 1/128 Hz was applied. We controlled the data for spatial abnormalities (e.g., dropouts) with the Mask Explorer tool[56], as well as for artifacts related to excessive head movement using framewise displacement (FD) criterion.[57]
To identify different resting-state brain networks, we run independent component analysis (ICA) using the GIFT toolbox (https://trendscenter.org/software/gift/).[58] ICA was performed with the INFOMAX and GICA back-reconstruction algorithms. Component reliability was determined with the ICASSO toolbox.[59] The optimal number of components was based on the minimum description length criterion.[60] Altogether 18 stable components were identified, including the basal ganglia brain network (Supplementary eFigure 6).

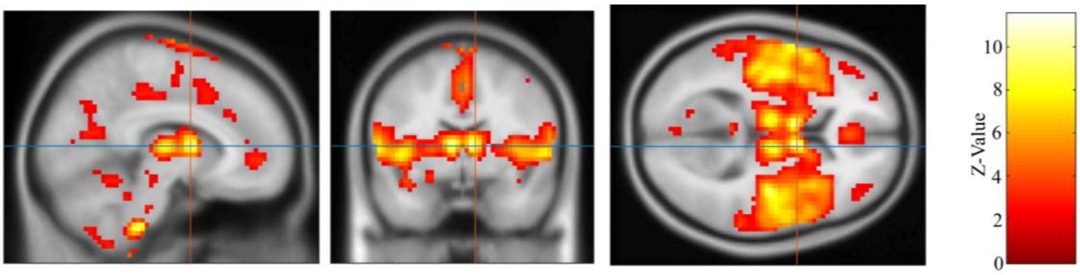

eFigure 6. ICA component representing basal ganglia network.
We thresholded the component for the visualization on $z > 1.96$.

13. Details on neuroimaging statistical analysis

Pre-post rs-fMRI analysis compared baseline pre-stimulation Z-values using a separate LMM with stimulation condition as a fixed factor and participant as a random intercept. Associations between (i) connectivity changes (Z-values) (ii) pre-stimulation connectivity and stimulation-induced normalized behavioural performance changes were assessed using Pearson correlations with FDR correction for multiple comparisons. To investigate the relationship between normalized regional brain volumes (i.e., T1-weighted MRI images) and behavioural performance, we employed a custom-developed MATLAB (version 2024b) pipeline implementing bisquare robust linear regression analyses through the built-in *fitlm* function. Specifically, we analysed differences in Accuracy and RTs across stimulation (Active control, $TMS_{control}$+$tTIS_{str}$, and TMS_{cb}+$tTIS_{str}$) and task conditions (high-load vs. distraction condition) in which our intervention showed some behavioural changes.

eResults

1. Neuropsychological profiles across cohorts

Domain Z-scores (average z-scores of tests within each domain) were compared across cohorts using one-way ANOVA with cohort as main effect, followed by Tukey's post-hoc correction. A significant main effect of cohort was found for MoCA ($p = 0.002$, $p\eta^2 = 0.36$), visual/verbal memory ($p = 0.001$, $p\eta^2 = 0.4$; $p = 0.005$, $p\eta^2 = 0.4$), and executive functions ($p < 0.001$, $p\eta^2 = 0.35$). After post-hoc comparisons, the MCI-LB group showed significant deficits in visual memory (adj. $p < 0.001$, $|d| = 1.08$) and executive functions (adj. $p = 0.001$, $|d| = 1.02$). The aMCI had significantly lower scores in MoCA (adj. $p < 0.001$, $|d| = 1.73$), visual memory (adj. $p < 0.001$, $|d| = 1.86$), verbal memory (adj. $p < 0.001$, $|d| = 1.85$), and executive functions (adj. $p < 0.001$, $|d| = 0.96$). The aMCI group exhibited significantly lower scores as compared to MCI-LB in visual (adj. $p = 0.02$, $|d| = 0.87$) and verbal memory (adj. $p < 0.001$, $|d| = 1.52$), and MoCA (adj. $p < 0.001$, $|d| = 1.33$).

		Normalized ACC Distraction											
		1st block			2nd block			3rd block			Post-block		
**Cohort**	**Stimulation Condition**	**Mean**	**±**	**SE**	**Mean**	**±**	**SE**	**Mean**	**±**	**SE**	**Mean**	**±**	**SE**
MCI-LB	Active control	0,98	±	0,03	0,96	±	0,04	0,99	±	0,03	1,01	±	0,03
	n		20			20			20			20	
	$TMS_{control}$+$tTIS_{str}$	1,02	±	0,04	1,00	±	0,03	0,99	±	0,04	1,06	±	0,04
	n		21			21			21			20	
	TMS_{cb}+$tTIS_{str}$	1,08	±	0,05	1,16	±	0,08	1,13	±	0,07	1,14	±	0,07
	n		20			20			20			20	
aMCI	Active control	1,02	±	0,05	1,08	±	0,06	1,01	±	0,05	1,04	±	0,04
	n		20			20			20			20	
	$TMS_{control}$+$tTIS_{str}$	1,00	±	0,05	1,08	±	0,07	1,05	±	0,06	1,08	±	0,08
	n		20			20			20			20	
	TMS_{cb}+$tTIS_{str}$	0,98	±	0,05	1,03	±	0,05	0,98	±	0,03	0,92	±	0,04
	n		20			20			20			20	

eTable 7. Normalized Distraction Accuracy Across Blocks and Stimulation Conditions

		Normalized ACC High load											
		1st block			2nd block			3rd block			Post-block		
**Cohort**	**Stimulation Condition**	**Mean**	**±**	**SE**	**Mean**	**±**	**SE**	**Mean**	**±**	**SE**	**Mean**	**±**	**SE**
MCI-LB	Active control	1,05	±	0,05	1,05	±	0,05	1,09	±	0,07	1,09	±	0,06
	n		20			20			20			20	
	$TMS_{control}$+$tTIS_{str}$	0,99	±	0,06	1,06	±	0,05	1,08	±	0,09	1,01	±	0,07
	n		21			21			21			20	
	TMS_{cb}+$tTIS_{str}$	1,01	±	0,05	1,01	±	0,05	1,07	±	0,04	1,02	±	0,04
	n		20			20			20			20	
aMCI	Active control	1,04	±	0,08	1,02	±	0,08	1,13	±	0,08	1,07	±	0,09
	n		20			20			20			20	

© 2026 Nencha U et al. *JAMA Network Open*.

	TMS$_{control}$+tTIS$_{str}$	1,10	±	0,09	1,15	±	0,09	1,14	±	0,08	1,09	±	0,06
	n		20			20			20			20	
	TMS$_{cb}$+tTIS$_{str}$	1,08	±	0,06	1,01	±	0,06	1,08	±	0,07	1,06	±	0,06
	n		20			20			20			20	

eTable 8. Normalized High-Load Accuracy Across Blocks and Stimulation Conditions

		Normalized RT Distraction											
		1st block			2nd block			3rd block			Post-block		
Cohort	Stimulation Condition	Mean	±	SE	Mean	±	SE	Mean	±	SE	Mean	±	SE
MCI-LB	Active control	0,98	±	0,03	0,98	±	0,03	1,02	±	0,04	0,98	±	0,04
	n		20			20			20			20	
	TMS$_{control}$+tTIS$_{str}$	0,99	±	0,03	0,94	±	0,03	0,94	±	0,03	0,97		0,07
	n		21			21			21			20	
	TMS$_{cb}$+tTIS$_{str}$	1,01	±	0,03	0,99	±	0,03	0,99	±	0,04	0,93	±	0,03
	n		20			20			20			20	
aMCI	Active control	1,04	±	0,03	0,98	±	0,03	0,99	±	0,03	1,00	±	0,03
	n		20			20			20			20	
	TMS$_{control}$+tTIS$_{str}$	1,04	±	0,04	0,97	±	0,04	0,96	±	0,04	0,97	±	0,03
	n		20			20			20			20	
	TMS$_{cb}$+tTIS$_{str}$	0,97	±	0,04	0,96	±	0,03	0,93	±	0,04	0,94	±	0,03
	n		20			20			20			20	

eTable 9. Normalized Distraction RT Across Blocks and Stimulation Conditions

		Normalized RT High Load											
		1st block			2nd block			3rd block			Post-block		
Cohort	Stimulation Condition	Mean	±	SE	Mean	±	SE	Mean	±	SE	Mean	±	SE
MCI-LB	Active control	0,97	±	0,03	0,96	±	0,03	0,96	±	0,03	0,89	±	0,03
	n		20			20			20			20	
	TMS$_{control}$+tTIS$_{str}$	1,00	±	0,04	0,99	±	0,05	0,93	±	0,03	0,99	±	0,03
	n		21			21			21			20	
	TMS$_{cb}$+tTIS$_{str}$	1,03	±	0,03	1,01	±	0,03	0,99	±	0,03	0,99	±	0,04
	n		20			20			20			20	
aMCI	Active control	1,09	±	0,03	0,95	±	0,03	0,98	±	0,03	1,06	±	0,05
	n		20			20			20			20	
	TMS$_{control}$+tTIS$_{str}$	1,04	±	0,04	1,06	±	0,04	1,05	±	0,04	1,01	±	0,03
	n		20			20			20			20	
	TMS$_{cb}$+tTIS$_{str}$	1,00	±	0,04	1,00	±	0,04	0,97	±	0,03	0,96	±	0,03
	n		20			20			20			20	

© 2026 Nencha U et al. *JAMA Network Open*.

eTable 10. Normalized High-Load RT Across Blocks and Stimulation Conditions

1.1. aMCI online behavioral performance

During online performance, the factor stimulation did show a trend toward a main effect on RTs in high-load condition ($p = 0.07$, p$\eta^2 = 0.04$). Post-hoc comparisons indicated faster RTs during TMS$_{cb}$+tTIS$_{str}$ (0.99, 95% CI 0.94-1.04) compared to TMS$_{control}$+tTIS$_{str}$ (1.05, 95% CI 1.00-1.10; $p = 0.07$, $d = 0.41$). This was further supported by the Bayesian analysis, indicating moderate evidence (eResults in Supplement, eFigure 7). There was no significant main effect of block ($p = 0.10$). However, a trend in the stimulation × block interaction was observed ($p = 0.06$), with significant decreases in RTs (block 1 - block 2, $p = 0.003$; block 1 - block 3, $p = 0.02$) in the Active control.

2. Post-stimulation behavioral changes

In MCI-LB, RTs for high-load trials showed a trend for stimulation effect ($p = 0.05$, p$\eta^2 = 0.10$), with smaller RTs for Active control (mean 0.89, 95% CI 0.82-0.95) versus TMS$_{cb}$+tTIS$_{str}$ and TMS$_{control}$+tTIS$_{str}$ (both mean 0.99, 95% CI 0.92-1.10; $p = 0.09$, $d = -0.68$). Accuracy was unaffected in post-block analyses (all $p > 0.13$). No trends or significant effects were observed for Accuracy in post-block analyses, either in distraction or high-load trials. Similarly, RTs in distraction trials showed no stimulation effects, including after log-transformation to meet normality assumptions, which did not alter the results.
In aMCI, a trend toward higher Accuracy during TMS$_{control}$+tTIS$_{str}$ (mean 1.08, 95% CI 0.97-1.20) compared to TMS$_{cb}$+tTIS$_{str}$ was found in the distraction condition (mean 0.92, 95% CI 0.80-1.03; $p = 0.08$, $d = 0.71$); all other effects on RTs and Accuracy were non-significant (all $p > 0.16$).

3. Bayesian Analysis of Accuracy in the Distraction Condition – MCI-LB Cohort

Model convergence was excellent, with all Rhat values equal to 1.00 and high effective sample sizes for all parameters (Bulk and Tail ESS > 29,000), indicating stable and well-mixed Markov chains. Posterior summaries revealed a credible positive effect for the TMS$_{cb}$+tTIS$_{str}$ stimulation compared to Active control stimulation (Estimate = 0.14, 95% CI = [0.07, 0.21]), suggesting improved Accuracy under this condition. In contrast, the effect of TMS$_{control}$+tTISstr stimulation was small and uncertain (Estimate = 0.03, 95% CI = [−0.04, 0.10]).
Bayes factors calculated using the Savage–Dickey density ratio method further supported these findings. There was substantial evidence in favor of a TMS$_{cb}$+tTIS$_{str}$ effect (BF$_{10}$ = 6.09), while all other terms showed strong evidence against an effect (BF$_{10}$ < 0.02), reinforcing the null hypothesis that these parameters have no impact on the outcome. This pattern suggests a selective benefit of TMS$_{cb}$+tTIS$_{str}$ on task Accuracy, which was not modulated by block.
Posterior predictive checks demonstrated strong model suitability. Simulated data from the posterior closely matched the observed data in terms of density, spread, and quantiles. For example, the predicted SD of the outcome (mean = 0.231) was nearly identical to the observed SD (0.229). Predicted quantiles (25%: 0.87, 50%: 1.01, 75%: 1.18) also aligned well with observed values (25%: 0.91, 50%: 1.00, 75%: 1.10). The posterior predictive checks (density overlays, quantiles, SD comparisons, ECDF plots) show that the model-generated data looks very similar to the actual observed data (Supplementary eFigure 7).
LOO cross-validation confirmed good model fit, with a LOOIC of −60.1 and all Pareto k values < 0.7, indicating reliable estimation and no influential data points.

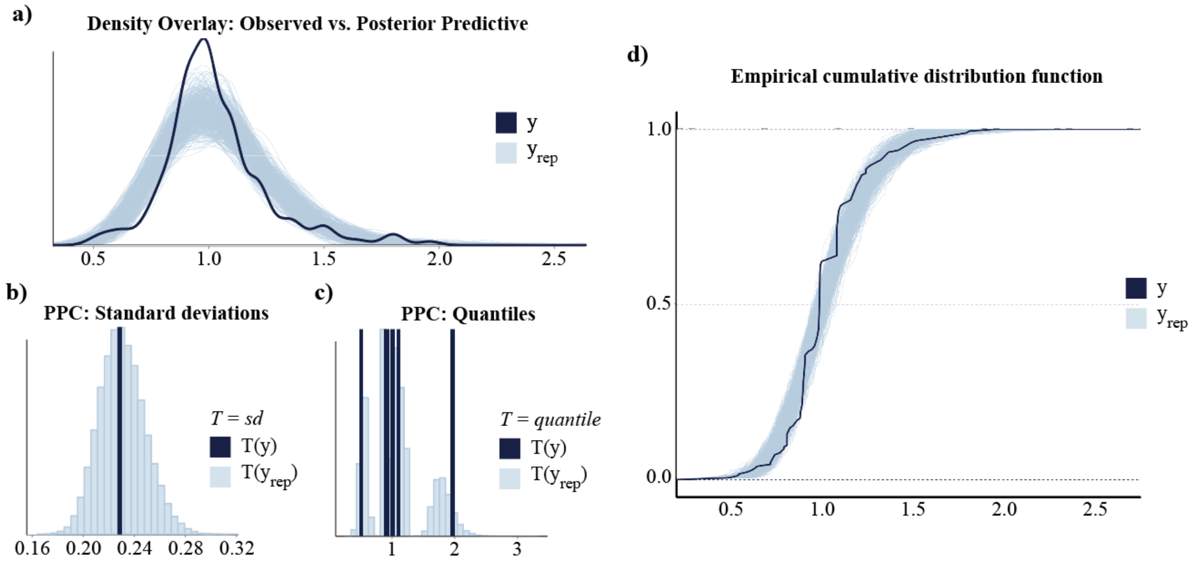

© 2026 Nencha U et al. *JAMA Network Open*.

eFigure 7. Posterior predictive checks for the Bayesian model assessing stimulation effects on Accuracy during the distraction condition in the MCI-LB Cohort.
a) Overlay of the observed data distribution (black line) and replicated data from the posterior predictive distribution (blue lines) across 500 draws, showing that the model reproduces the empirical density.
b) Comparison of the observed and predicted SD, indicating accurate recovery of variability.
c) Comparison of the 25th, 50th, and 75th percentiles between observed and predicted data.
d) Empirical cumulative distribution function (ECDF) comparison further confirms alignment between the observed and model-predicted distributions.
PPC = Posterior Predictive Checks, y = observed data, y_{rep} = posterior predictive replicated data, T(y) = summary statistic of the observed data, T(y_{rep}) = summary statistic calculated across all replicated dataset.

4. Bayesian Analysis of Reaction Times in the High-load Condition – aMCI Cohort

Model diagnostics indicated excellent convergence, with all Rhat values at 1.00 and very large effective sample sizes across parameters (Bulk and Tail ESS > 29,000), suggesting well-behaved and efficiently mixed Markov chains. Posterior estimates revealed a modest but credible reduction in RTs for the TMS_{cb}+$tTIS_{str}$ condition compared to $TMS_{control}$+tTISstr condition (Estimate = −0.05, 95% CI = [−0.11, −0.00]), pointing to faster task performance under this stimulation type. Conversely, the estimated effect for the Active control compared to $TMS_{control}$+$tTIS_{str}$ was statistically inconclusive (Estimate = −0.04, 95% CI = [−0.09, 0.01]).
Bayes factors computed via the Savage–Dickey method provided additional evidence: while support for a TMS_{cb}+$tTIS_{str}$ effect was weak-to-moderate (BF_{10} = 0.047), all remaining terms favored the null hypothesis strongly (BF_{10} < 0.03).
Posterior predictive checks confirmed the model's strong performance in capturing the distribution of observed data. Simulated RTs closely matched the empirical distribution in density, spread, and key quantiles; the model-predicted SD (mean = 0.173) was nearly indistinguishable from the observed SD (0.162). Predicted quantiles (25%: 0.90, 50%: 1.00, 75%: 1.12) also tracked closely with the empirical values (25%: 0.90, 50%: 1.01, 75%: 1.12), demonstrating good fidelity to the shape of the data (Supplementary eFigure 8).
LOO cross-validation further validated the model's robustness, yielding a LOOIC of −170.0 and Pareto k estimates all below 0.7, indicating reliable estimates and no influence data point.

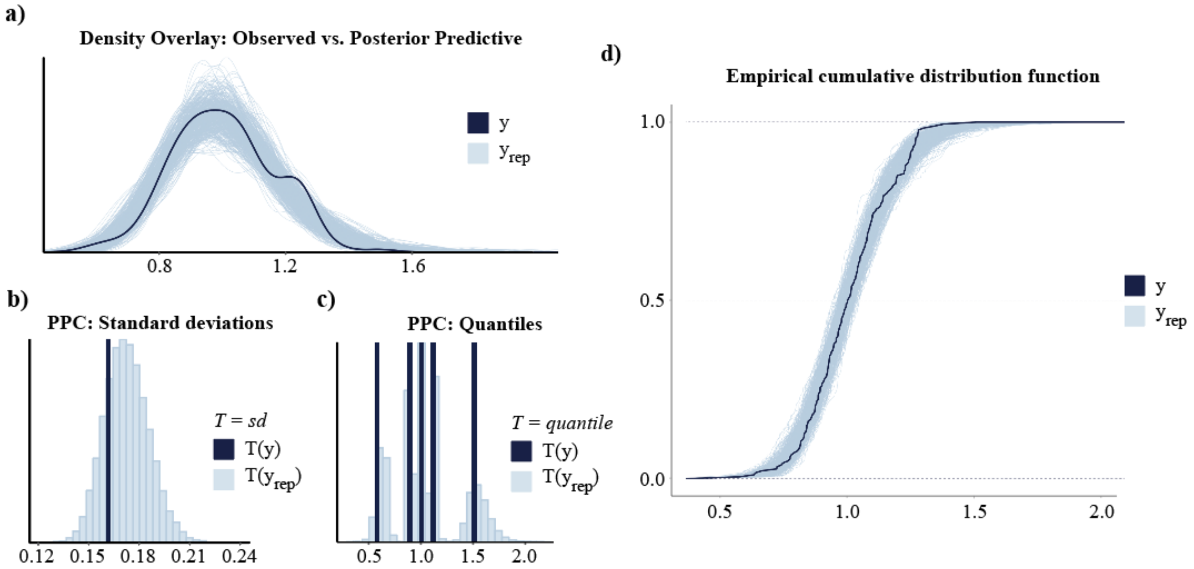

eFigure 8. Posterior predictive checks for the Bayesian model assessing stimulation effects on RTs during the distraction condition in the aMCI Cohort.
a) Overlay of the observed data distribution (black line) and replicated data from the posterior predictive distribution (blue lines) across 500 draws, showing that the model reproduces the empirical density.
b) Comparison of the observed and predicted SD, indicating accurate recovery of variability.
c) Comparison of the 25th, 50th, and 75th percentiles between observed and predicted data.
d) Empirical cumulative distribution function (ECDF) comparison further confirms alignment between the observed and model-predicted distributions.
PPC = Posterior Predictive Checks, y = observed data, y_{rep} = posterior predictive replicated data, T(y) = summary statistic of the observed data, T(y_{rep}) = summary statistic calculated across all replicated datasets.

5. Stimulation effect in the healthy control cohort

In the HC cohort (Supplementary eFigure 9), during the distraction condition, there was a trend toward a main effect of stimulation on RTs ($p = 0.06$), although post-hoc comparisons did not reveal any significant differences. A Bayesian analysis suggested a modest but statistically credible reduction in RTs during TMS_{cb}+$tTIS_{str}$ compared to Active control condition (Estimate = −0.05, 95% CI [−0.09, −0.00]). However, the Bayes Factor ($BF_{10} = 0.033$) provided moderate evidence in favor of the null hypothesis, indicating that this apparent effect should be interpreted with caution. All checks confirmed good model fit and reliable parameter estimation. There were no significant main effects of block ($p = 0.53$) or stimulation × block interaction ($p = 0.86$) on RTs. Accuracy was similarly unaffected by stimulation ($p = 0.58$), block ($p = 0.78$), or their interaction ($p = 0.77$).

In the high-load condition, the factor stimulation had a significant main effect on Accuracy ($p = 0.03$, $p\eta^2 = 0.04$). Post-hoc comparisons revealed higher Accuracy in the Active control condition compared to TMS_{cb}+$tTIS_{str}$ ($p = 0.03$, $d = 0.48$). Bayesian analysis suggested a similar direction of effect (Estimate = −0.13, 95% CI [−0.22, −0.03]), indicating lower Accuracy during TMScb+tTISstr compared to Active control. However, the Bayes Factor ($BF_{10} = 0.28$) indicated moderate evidence in favor of the null hypothesis, suggesting that the evidence for a true disturbance effect of TMS_{cb}+$tTIS_{str}$ relative to Active control is moderate. No significant main effects of block ($p = 0.75$) or stimulation × block interaction ($p = 0.97$) were observed for Accuracy. RTs were not significantly influenced by stimulation ($p = 0.76$), block ($p = 0.90$), or their interaction ($p = 0.58$).

In the post-stimulation block analysis of the HC cohort, the LMM revealed no significant effects or trends in Accuracy or RTs across stimulation conditions. Accuracy in the distraction and high-load conditions showed no differences ($p = 0.31$ and $p = 0.73$, respectively), nor did RTs in these conditions ($p = 0.91$ and $p = 0.99$, respectively).

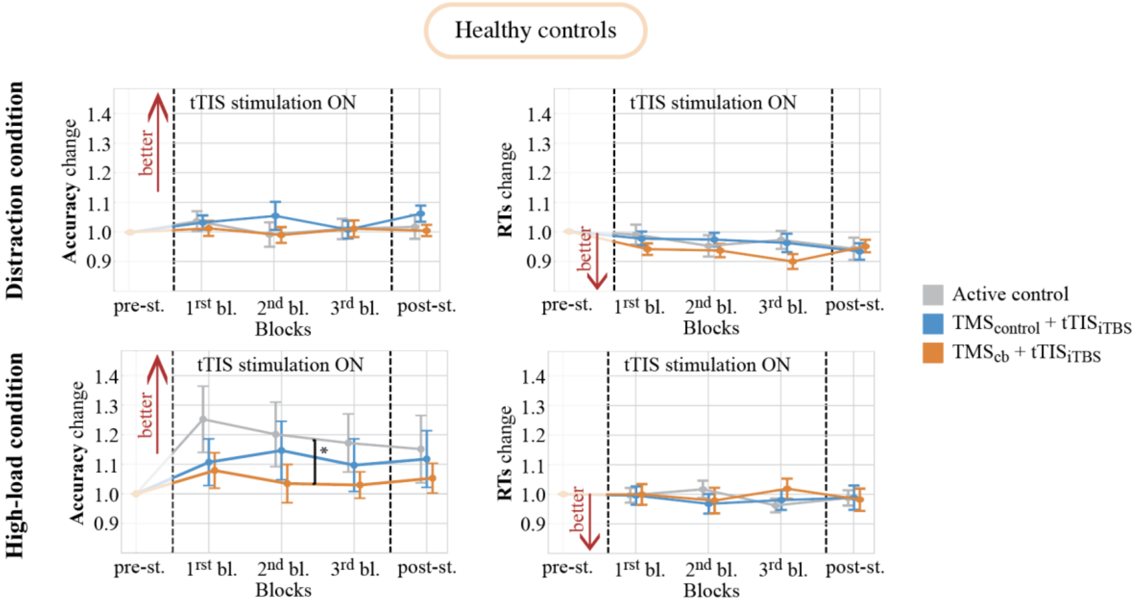

eFigure 9. Stimulation effects on visuospatial WM performance in HC cohort (n = 20).

During the distraction condition, no significant main effects were observed. In the high-load condition, the factor stimulation had a significant main effect on Accuracy, with higher performance during Active control compared to TMS_{cb}+$tTIS_{str}$. No significant main effects were found for RTs.

Lines represent group means across blocks, with error bars indicating standard error of the mean (SEM). *$p < 0.05$; †trend $p < 0.10$. Acc = Accuracy; RTs = Mean Reaction Times; Pre-st. = pre-stimulation block; Post-st. = post-stimulation block; Bl. = blocks.

6. Adverse Events, perceived sensations and blinding efficacy

During the course of the study, no harms or unintended adverse events were observed in any of the participants in either group. Perceived sensations and blinding efficacy were only evaluated in the EPFL cohort. Overall, attention levels in MCI did not change before and after stimulation sessions, regardless of the stimulation type. Although patients reported feeling more fatigued after interventional sessions, this effect reached significance only during the Active control session. Furthermore, overall fatigue changes were not dependent on the type of stimulation. There were also no differences across stimulation days in how long participants slept or how sleepy they felt. Similarly, stimulation conditions had no effect on emotional states (including anger, anxiety, desire,

disgust, fear, happiness, relaxation, or sadness) nor on impulsive decision-making (Supplementary eFigures 10, 11 and 12). During the evaluation of perceived tTIS sensations, prior to stimulation, at the experimental stimulation intensity (2mA), nearly half of the patients and participants reported no sensation during $tTIS_{control}$ or $tTIS_{str}$ in the titration phase. Only 6.7% reported a strong sensation during $tTIS_{control}$, and 4.4% during $tTIS_{str}$ (Supplementary eFigure 13a). Among individuals who did report a sensation, tingling was the most frequently described (Supplementary eFigure 13b). These findings are comparable with current literature.[1,50,73,74] At the end of all three experimental sessions, participants at EPFL were asked to report any sensations experienced during concurrent tTIS and task performance for each session, and to guess whether they had received $tTIS_{str}$ or $tTIS_{control}$ in each case. Participants were unable to reliably distinguish between the $tTIS_{control}$ and $tTIS_{str}$. Among those who attempted to identify the tTIS session, accuracy of their guesses did not exceed chance level (binomial test: $p = 0.36$; Supplementary eFigure 13c).

© 2026 Nencha U et al. *JAMA Network Open*.

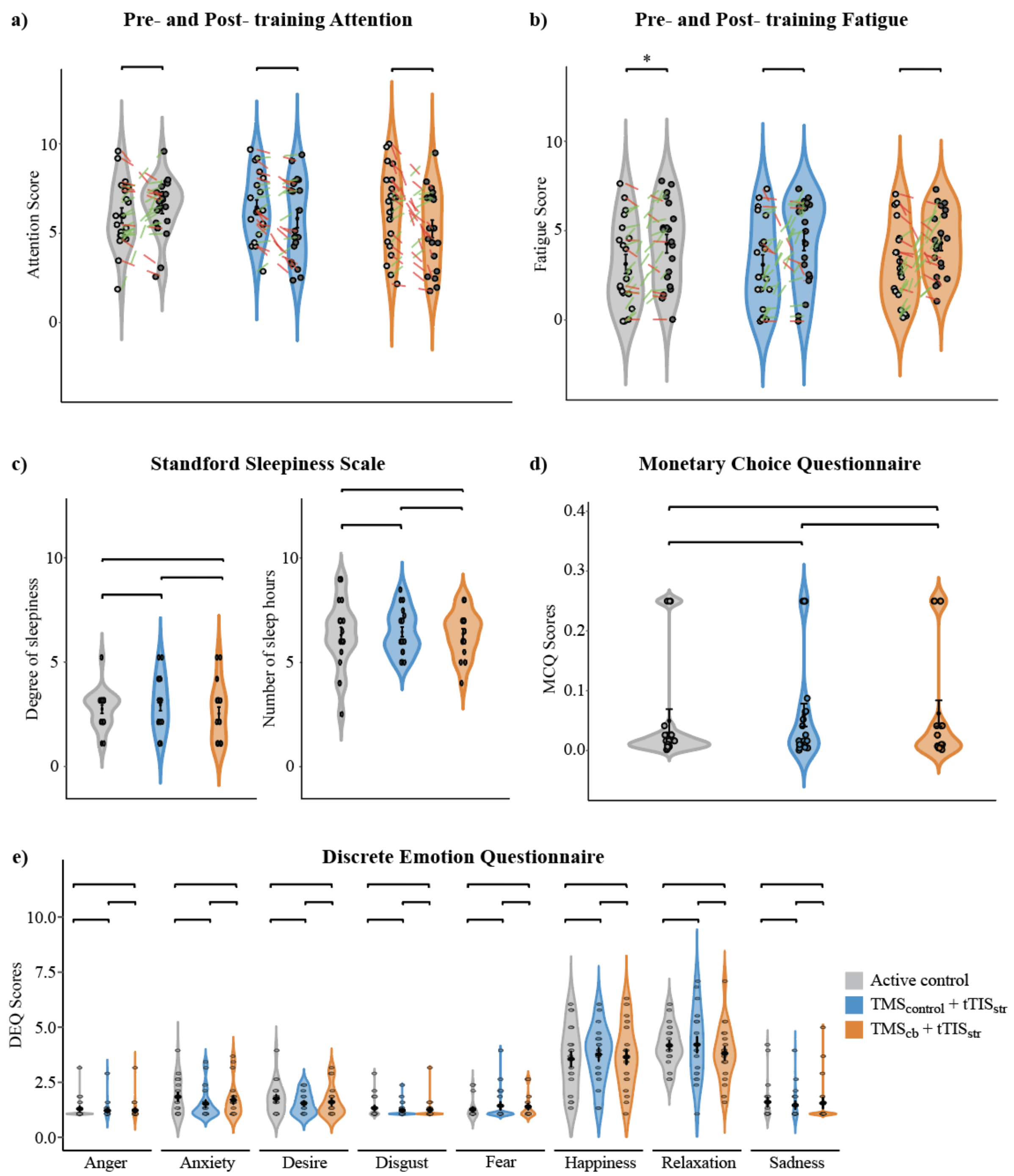

eFigure 10. Effects of stimulation on attention, fatigue, sleep, decision-making, and emotion in the EPFL MCI cohort.

Distributions of scores across stimulation types (Active control, $TMS_{control}$+$tTIS_{str}$, and TMS_{cb}+$tTIS_{str}$) are shown for each measure. Violin plots indicate the score distributions, with individual data points, group means, and standard errors overlaid. Before performing group comparisons, we assessed the normality of each group's data distribution using the Shapiro-Wilk test. If all groups for a given measure were normally distributed, we used parametric t-tests for comparisons; if any group was not normally distributed, we used the non-parametric Wilcoxon rank-sum test instead. Adjusted p-values (Bonferroni correction) are displayed on each plot.

a) A slight, non-significant decrease in attention was observed post-stimulation, with no dependence on stimulation type.

b) Fatigue increased slightly post-stimulation in the aMCI cohort, with significance observed only in the Active control condition. Furthermore, the fatigue deltas were not dependent on stimulation type.

c) No differences were found in sleep duration or subjective sleepiness across stimulation conditions.

d) Stimulation had no effect on impulsive decision-making.

© 2026 Nencha U et al. *JAMA Network Open*.

e) No changes were observed in emotional states, including anger, anxiety, desire, disgust, fear, happiness, relaxation, or sadness.
*$p < 0.05$.

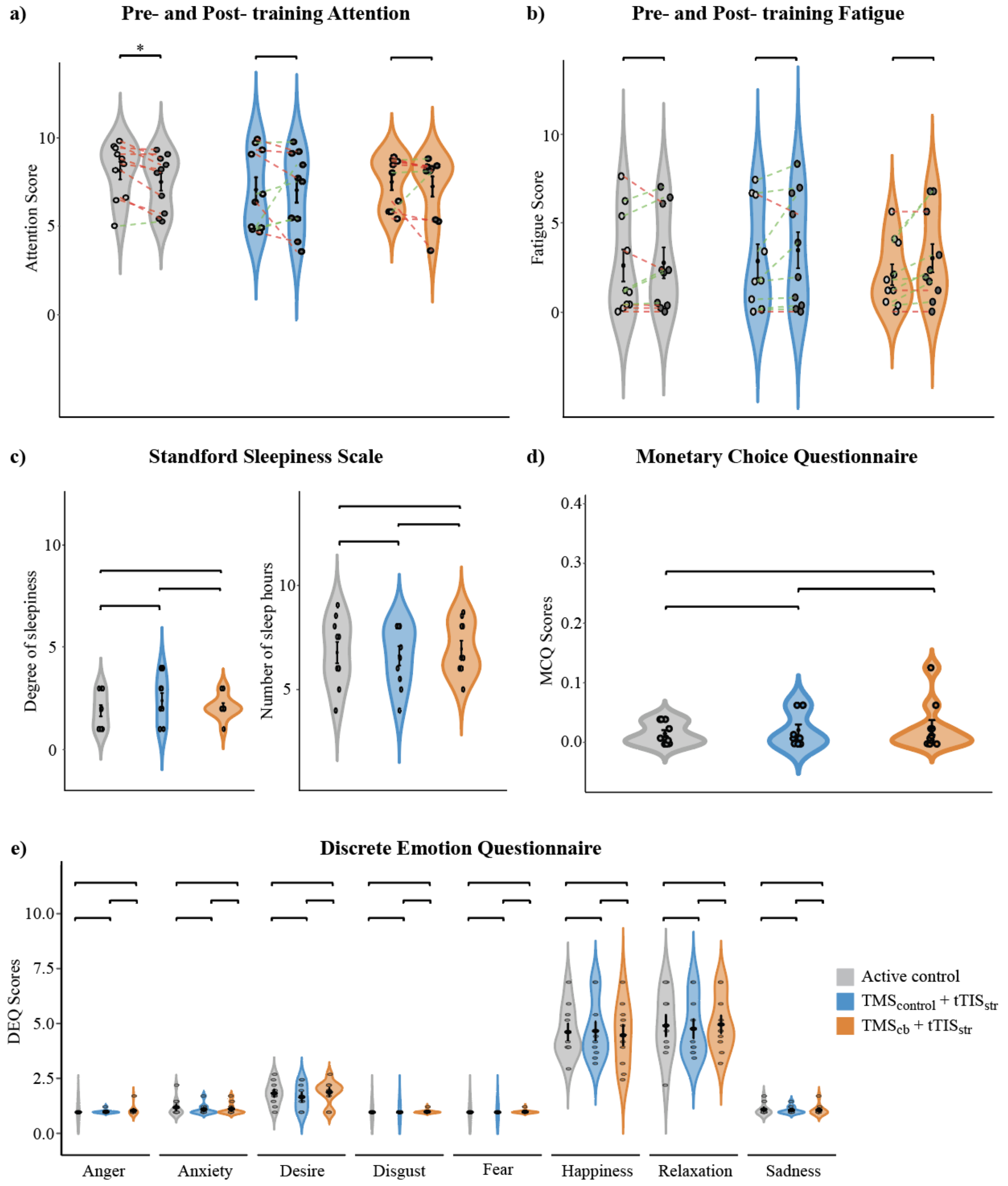

eFigure 11. Effects of stimulation on attention, fatigue, sleep, decision-making, and emotion in the EPFL HC cohort.
Distributions of scores across stimulation types (Active control, $TMS_{control}$+$tTIS_{str}$ and TMS_{cb}+$tTIS_{str}$) are shown for each measure. Violin plots indicate the score distributions, with individual data points, group means, and standard errors overlaid. Before performing group comparisons, we assessed the normality of each group's data distribution using the Shapiro-Wilk test. If all groups for a given measure were normally distributed, we used parametric t-tests for comparisons; if any group was not normally distributed, we used the non-parametric Wilcoxon rank-sum test instead. Adjusted p-values (Bonferroni correction) are displayed on each plot.

© 2026 Nencha U et al. *JAMA Network Open*.

a) A significant decrease in attention was observed post-stimulation only in the Active control, with no significant change following $TMS_{control}$+$tTIS_{str}$ or TMS_{cb}+$tTIS_{str}$ sessions. However, no dependence on stimulation type was seen.
b) Fatigue slightly increased post-stimulation, never reaching significance, with no dependence on stimulation type.
c) No differences were found in sleep duration or subjective sleepiness across stimulation conditions.
d) Stimulation had no effect on impulsive decision-making.
e) No changes were observed in emotional states, including anger, anxiety, desire, disgust, fear, happiness, relaxation, or sadness.
*$p < 0.05$.

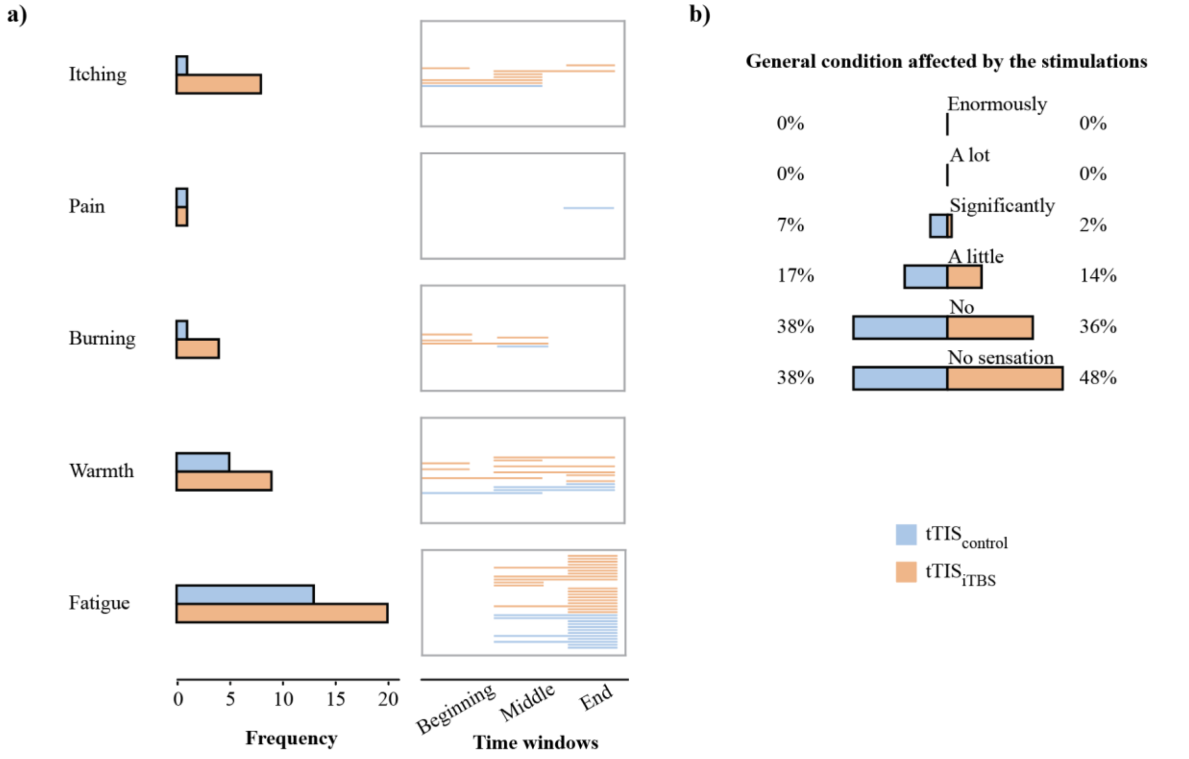

eFigure 12. Sensations reported for tTIS by EPFL participants.
This figure summarizes the sensations reported by EPFL participants following tTIS. Participants completed a questionnaire after all the three stimulation sessions, without knowing whether they received $tTIS_{control}$ or $tTIS_{str}$. Due to the study design, twice as many sessions involved $tTIS_{str}$, resulting in 60 $tTIS_{str}$ sessions and 30 $tTIS_{control}$ sessions. Five sessions were excluded due to lack of clear memory from participants.
a) Participants were asked whether they experienced any itching, pain, burning, warmth, fatigue, or metallic taste during the stimulation sessions. No participant reported a metallic taste. For $tTIS_{control}$, sensations were reported in one session for itching, one for pain, one for burning, five for warmth, and 13 for fatigue, with one additional report of a pressure sensation. For $tTIS_{str}$, itching was reported in eight sessions, pain in one, burning in four, warmth in nine, and fatigue in 20. Additional sensations including tingling (two times), tickling (one time), and pressure (one time) were reported during $tTIS_{str}$. While absolute counts are shown, these should be interpreted relative to the total number of sessions per condition. Time windows showed that itching and warmth were variably distributed throughout the sessions; burning occurred mainly at the beginning; pain was reported at the end (though timing data for $tTIS_{str}$-related pain is missing); and fatigue typically began in the middle or toward the end of the tTIS.
b) Participants also indicated how much these sensations affected their general condition. For $tTIS_{control}$, 38% reported no sensation, 38% reported sensations with no impact, 17% reported slight impact, and 7% reported being significantly affected. For $tTIS_{str}$, 48% reported no sensation, 36% no impact, 14% slight impact, and 2% significant impact. No participants in either group reported that the stimulation affected them a lot or enormously. These results suggest that while some sensations were reported, they were generally mild and had limited impact on participants' overall condition.

© 2026 Nencha U et al. *JAMA Network Open*.

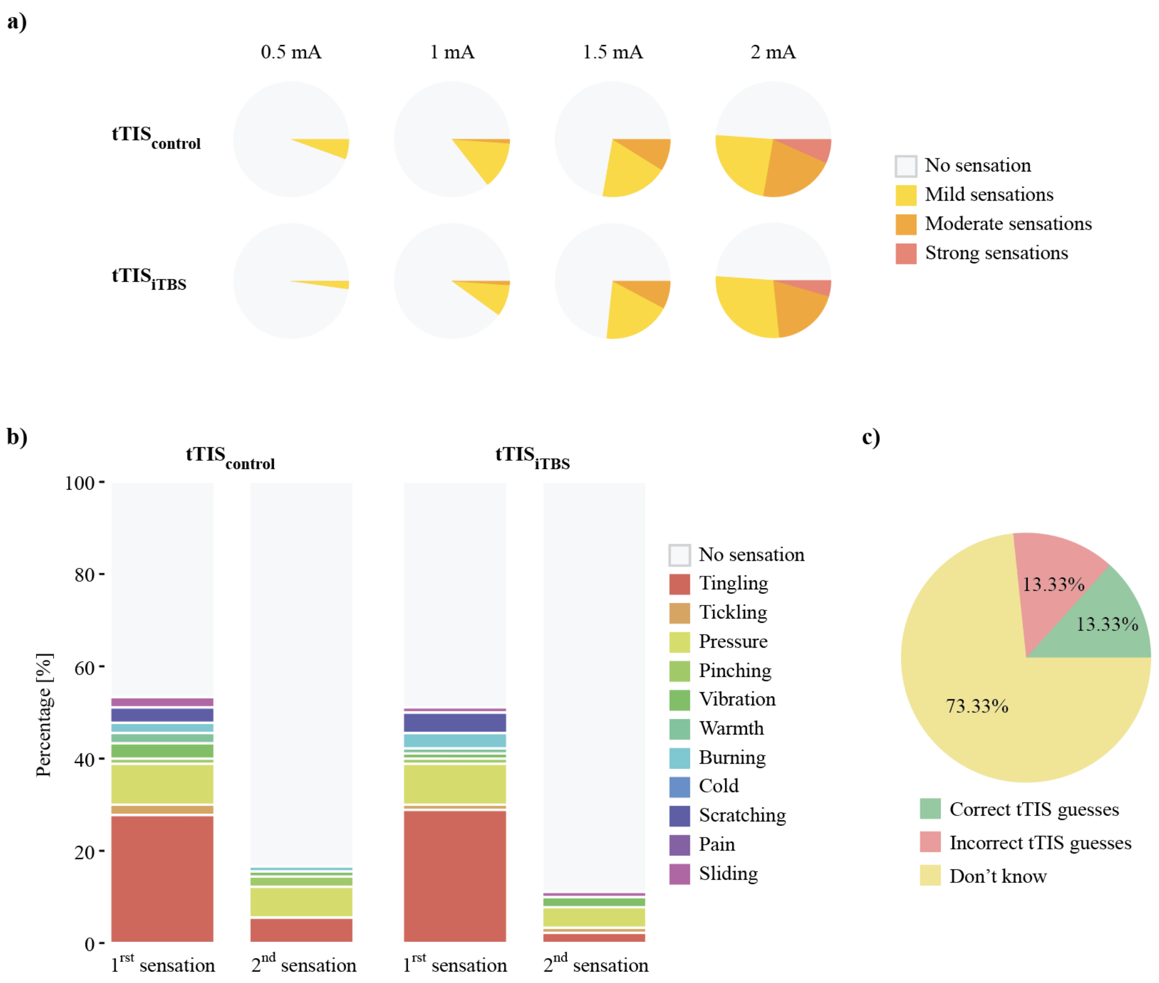

eFigure 13. Evaluation of perceived tTIS sensations and blinding efficacy to tTIS stimulation condition for EPFL participants.

a) Participants were exposed to two different tTIS protocols ($tTIS_{control}$ and $tTIS_{str}$) in short 20 second trials to assess perceived sensations while testing increasing current amplitudes per channel: 0.5 mA, 1 mA, 1.5 mA, and 2 mA. For each condition, participants rated any perceived sensation on a scale from 1 (mild) to 3 (strong) and provided descriptive adjectives (e.g., tingling, itching).

b) Frequency of the most commonly reported sensations across conditions, including first and second descriptors when applicable. Sensations were systematically recorded to characterize tolerability and subjective experience.

c) After completing all sessions, 30 EPFL subjects (90 tTIS sessions in total) were asked to identify which session involved which tTIS condition ($tTIS_{control}$ or $tTIS_{str}$). The majority answered "Don't know" (73.33%), while correct and incorrect guesses were equally distributed (13.33% each).

© 2026 Nencha U et al. *JAMA Network Open*.

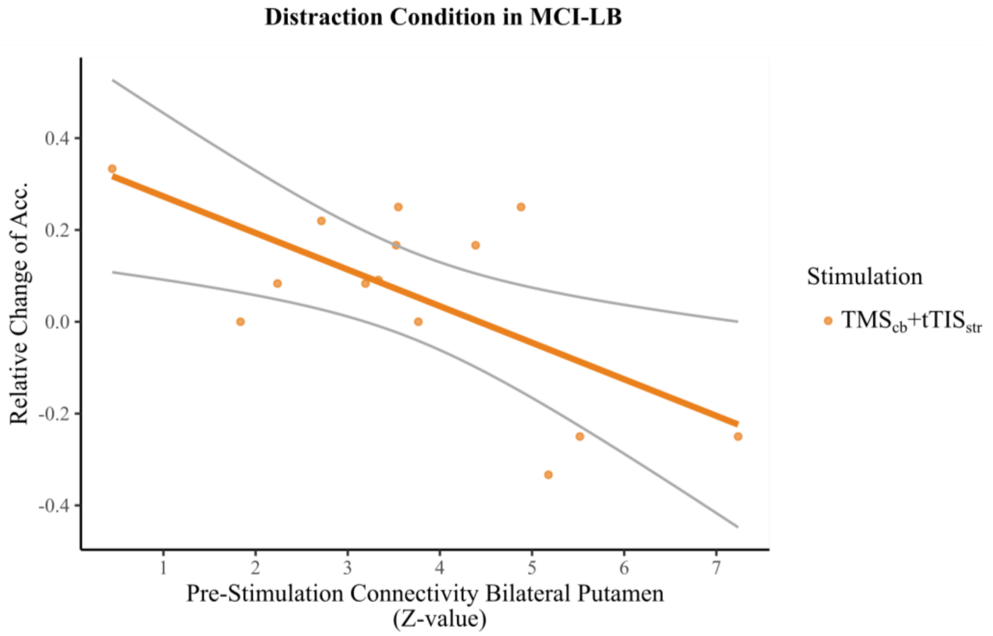

eFigure 14. Associations between pre-stimulation putaminal connectivity and stimulation-induced behavioural changes in the MCI-LB group.
Scatterplot illustrating a significant negative correlation (r = -0.66, p = 0.03) between pre-stimulation connectivity of the bilateral putamen and normalized Accuracy changes under trials involving a distractor, during the TMS_{cb}+$tTIS_{str}$ session.
Acc. = Accuracy.

Pre-stimulation connectivity from	Relative change of Acc. Distraction			
	Active control		TMS_{cb}+$tTIS_{str}$	
	r	p	r	p
Bilateral Striatum	-0.55	0.081	-0.25	0.572
Bilateral Putamen	-0.34	0.195	-0.66*	0.032
Bilateral Caudate	-0.39	0.195	0.03	0.915

eTable 11. Correlations between pre-stimulation basal ganglia connectivity and stimulation-induced changes in Accuracy under distraction in the MCI-LB group.
Shown are Pearson correlation coefficients (r) and FDR-corrected p-values for both Active control ($TMS_{control}$+$tTIS_{control}$) and TMS_{cb}+$tTIS_{str}$ conditions. The only significant correlation observed was between bilateral putamen connectivity and normalized Accuracy change in the TMS_{cb}+$tTIS_{str}$ condition.
*Significant correlation at FDR-corrected $p < 0.05$; Acc. = Accuracy.

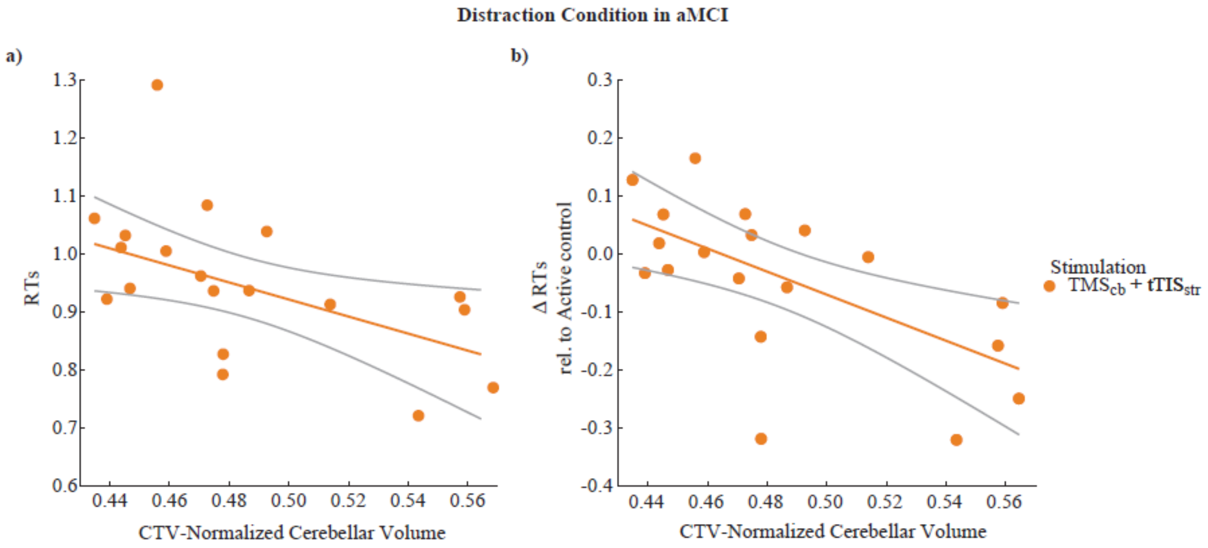

eFigure 15. Associations between cerebellar volume and behavioural performance in the aMCI group.
a) Scatter plot illustrating the negative correlation between CTV-normalized cerebellar volume and RTs during the online TMS$_{cb}$+tTIS$_{str}$ training condition in trials involving a distractor. RTs were normalized by calculating their relative ratio to the pre-stimulation baseline.
b) Scatter plot illustrating the negative correlation between CTV-normalized cerebellar volume and the difference in RTs between the TMS$_{cb}$+tTIS$_{str}$ and the Active control conditions. RTs during both Active control and TMS$_{cb}$+tTIS$_{str}$ conditions were normalized to the pre-stimulation baseline.
ΔRTs = RTs (TMS$_{cb}$+tTIS$_{str}$ - Active control), rel. = relative.

7. Supportive correlation results for structural imaging

To verify the validity of regression analyses results, considering deviations from normality in the data distribution, Spearman's rank correlation was also performed and supported both findings (RTs in TMS$_{cb}$+tTIS$_{str}$: ρ = -0.62, p = 0.006; RTs in TMS$_{control}$+tTIS$_{str}$: ρ = -0.51, p = 0.03, RTs difference between TMS$_{cb}$+tTIS$_{str}$ and Active control: ρ = -0.64, p = 0.004).

8. Stimulation effects in each cluster

In Cluster 0 (Supplementary eFigure 16a), a significant main effect of stimulation was found on Accuracy in the distraction condition (p = 0.002, $p\eta^2$ = 0.09), with a higher Accuracy during TMS$_{cb}$+tTIS$_{str}$ (mean 1.15, 95% CI 1.07-1.23) compared to TMS$_{control}$+tTIS$_{str}$ (mean 0.99, 95% CI 0.91-1.06; p = 0.002, d = -0.70) and a trend toward higher Accuracy during Active control (mean 1.09, 95% CI 1.01-1.17) compared to TMS$_{control}$+tTIS$_{str}$ (p = 0.06, d = 0.47). However, no main effect of stimulation was observed in RTs (p = 0.13). Similarly, no significant main effect of stimulation was observed on Accuracy (p = 0.61) or RTs (p = 0.91) in the high-load condition. Only a trend for an increase in speed (p = 0.07, $p\eta^2$ = 0.04) was observed between the first and the third block during the online performances (1^{rst} block: mean 1.02, 95% CI 0.97-1.07; 3rd block: mean 0.96, 95% CI 0.91-1.01; p = 0.07, d = 0.46). **In Cluster 1** (Supplementary eFigure 16b), a trend toward a main effect of stimulation on Accuracy in the distraction condition was observed (p = 0.06, $p\eta^2$ = 0.07), with higher performance during TMS$_{control}$+tTIS$_{str}$ (mean 1.06, 95% CI 0.98-1.13) compared to the Active control stimulation (mean 0.95, 95% CI 0.87-1.02; p = 0.06, d = -0.57). However, no significant main effect of stimulation was found for RTs (p = 0.21). In contrast, in the high-load condition, a stimulation had a significant main effect on Accuracy (p < 0.001, $p\eta^2$ = 0.18), with higher Accuracy during TMS$_{control}$+tTIS$_{str}$ (mean 1.30, 95% CI 1.19-1.41) compared to the Active control (mean 1.02, 95% CI 0.91-1.13; p = 0.001, d = -0.91) and TMS$_{cb}$+tTIS$_{str}$ (mean 1.02, 95% CI 0.91-1.13; p = 0.001, d = 0.90). Additionally, a significant main effect of stimulation was observed for RTs in the high-load condition (p = 0.03, $p\eta^2$ = 0.09). Post-hoc comparisons showed significantly faster RTs during the Active control (mean 0.97, 95% CI 0.88-1.05) compared to TMS$_{cb}$+tTIS$_{str}$ (mean 1.06, 95% CI 0.97-1.14; p = 0.05, d = -0.59) and a trend towards faster RTs in Active control compared to TMS$_{control}$+tTIS$_{str}$ (mean 1.06, 95% CI 0.97-1.14; p = 0.05, d = -0.58). **In Cluster 2** (Supplementary eFigure 16c), there was a trend toward a main effect stimulation on Accuracy in the distraction condition (p = 0.07, $p\eta^2$ = 0.05), while no such trend was observed for RTs (p = 0.17). Post-hoc analyses revealed trend for a higher Accuracy during TMS$_{control}$+tTIS$_{str}$ (mean 1.06, 95% CI 0.97-1.14) compared to the Active control (mean 0.97, 95% CI 0.88-1.05; p = 0.06, d = -0.52). In the high-load condition, no significant main effects were found for either Accuracy (p = 0.26) or RTs (p = 0.50).

© 2026 Nencha U et al. *JAMA Network Open*.

Additionally, no significant stimulation × block interactions were found in any condition across the three clusters. Furthermore, no main effect of stimulation was observed in the post-stimulation analysis.
In addition, cluster differences in demographic variables (age, sex, YOE), baseline neuropsychological performance profile at baseline and CTV-normalized regional volumes were assessed (Supplementary eFigure 17a and 17b). Cluster 2 had significantly lower YOE compared to Cluster 0 ($p = 0.01$) and significantly lower verbal memory scores as compared to Cluster 1 ($p = 0.02$). The volumes analyzed included the hippocampus, caudate, putamen, striatum, and cerebellum. Among these, only the cerebellar volume normalized by CTV showed a significant difference between Clusters 0 and 1, that survived correction for multiple comparisons ($p = 0.03$).

© 2026 Nencha U et al. *JAMA Network Open*.

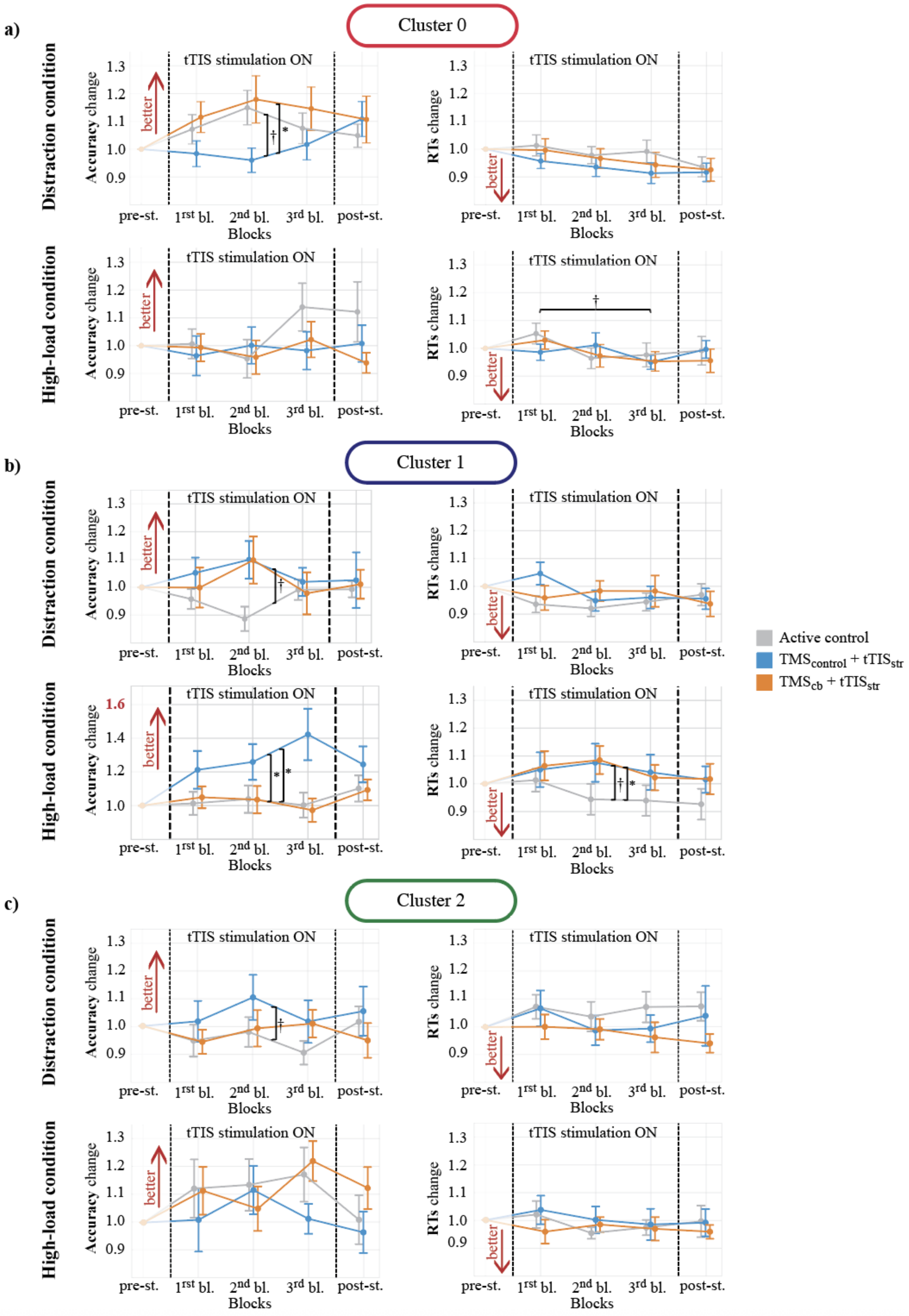

© 2026 Nencha U et al. *JAMA Network Open*.

eFigure 16. Stimulation effects on visuospatial WM performance across patient clusters.
Changes in Accuracy and RTs were calculated as a ratio relative to the pre-stimulation block.
a) Cluster 0: in the distraction condition, the factor stimulation showed a significant main effect on Accuracy, with higher performance during TMS_{cb}+tTIS_{str} vs. $\text{TMS}_{\text{control}}$+$\text{tTIS}_{\text{str}}$ and a trend for higher Accuracy in Active control vs. $\text{TMS}_{\text{control}}$+$\text{tTIS}_{\text{str}}$. No significant main effects were found on RTs or Accuracy in the high-load condition, though a trend for faster responses across blocks was observed.
b) Cluster 1: in the distraction condition, a trend toward a main effect of stimulation on Accuracy was found, with higher performance during $\text{TMS}_{\text{control}}$+$\text{tTIS}_{\text{str}}$ vs. Active control. In the high-load condition, the factor stimulation significantly affected both Accuracy and RTs, with $\text{TMS}_{\text{control}}$+$\text{tTIS}_{\text{str}}$ outperforming Active control and TMS_{cb}+tTIS_{str} in Accuracy, and Active control yielding faster RTs than the other conditions.
c) Cluster 2: in the distraction condition, the factor stimulation tended to influence Accuracy, with better performance during $\text{TMS}_{\text{control}}$+$\text{tTIS}_{\text{str}}$ vs. Active control. A trend toward faster RTs was observed for TMS_{cb}+tTIS_{str} vs. Active control. No additional trends or effects were observed.
Lines represent group means across blocks, with error bars indicating SEM. *$p < 0.05$; †trend $p < 0.10$; RTs = Mean Reaction Times; Pre-st. = pre-stimulation block; Post-st. = post-stimulation block; Bl. = blocks.

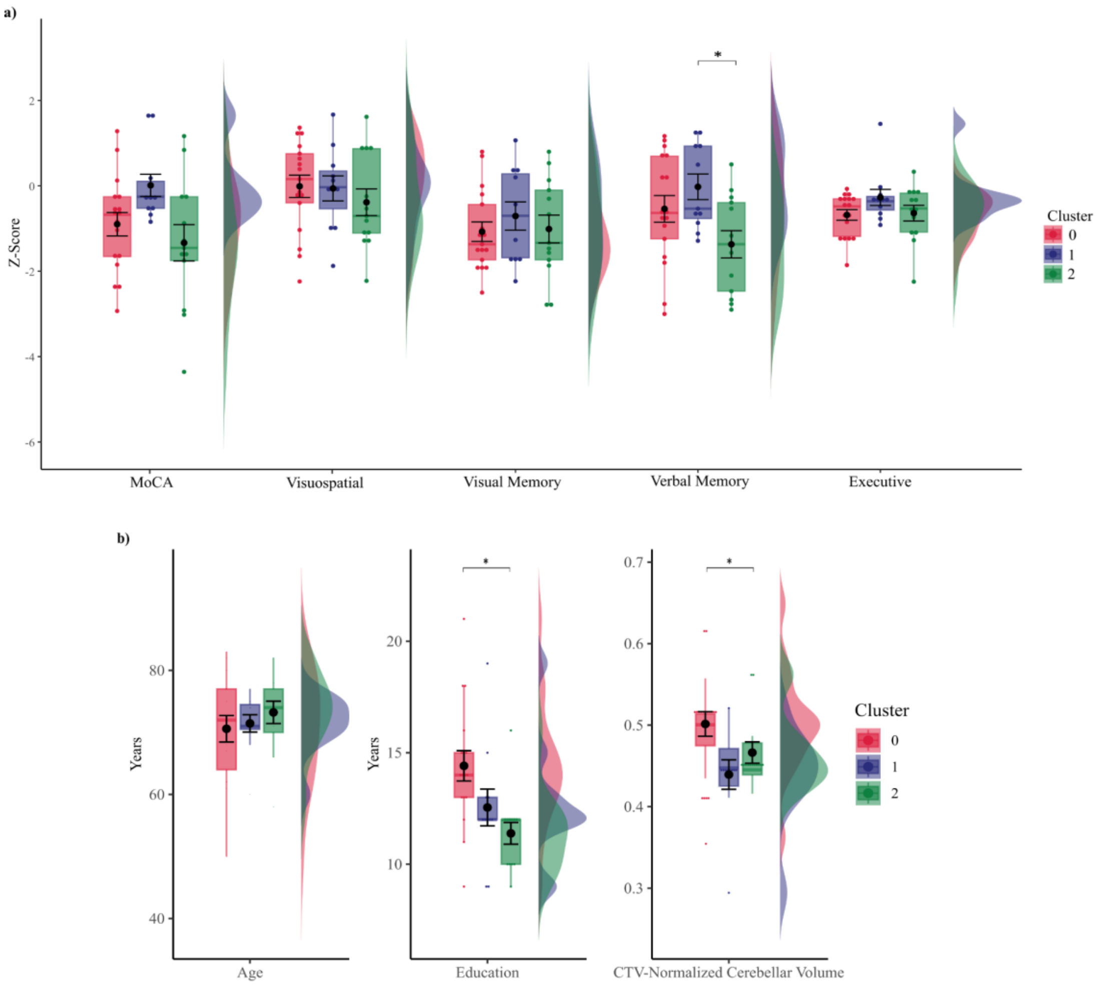

eFigure 17. Group differences across clusters in baseline neuropsychological performance (a) and demographic variables and regional brain volumes (b).
Cluster 2 showed significantly lower YOE compared to Cluster 0 ($p = 0.004$) and lower verbal memory scores compared to Cluster 1 ($p = 0.02$). Cluster 0 showed significantly higher cerebellar volume compared to Cluster 1 ($p = 0.03$).
*significant changes between clusters from post-hoc comparisons, multiple comparisons corrected $p < 0.05$.

© 2026 Nencha U et al. *JAMA Network Open*.

eReferences:

1. Wessel MJ, Beanato E, Popa T, et al. Noninvasive theta-burst stimulation of the human striatum enhances striatal activity and motor skill learning. *Nat Neurosci*. 2023;26(11):2005-2016. doi:10.1038/s41593-023-01457-7
2. Mitterová K, Výtvarová E, Kovářová A, Lamoš M, Fousek J, Rektorová I. Early functional changes in lewy body dementia: roles of dynamics, locus coeruleus, and compensation. *Alzheimers Res Ther*. 2025;17(1):199. doi:10.1186/s13195-025-01828-1
3. Železníková Ž, Nováková L, Vojtíšek L, et al. Early Changes in the Locus Coeruleus in Mild Cognitive Impairment with Lewy Bodies. *Mov Disord*. 2025;40(2):276-284. doi:10.1002/mds.30058
4. Novakova L, Gajdos M, Barton M, et al. Striato-cortical functional connectivity changes in mild cognitive impairment with Lewy bodies. *Parkinsonism Relat Disord*. 2024;121:106031. doi:10.1016/j.parkreldis.2024.106031
5. Albert MS, DeKosky ST, Dickson D, et al. The diagnosis of mild cognitive impairment due to Alzheimer's disease: Recommendations from the National Institute on Aging-Alzheimer's Association workgroups on diagnostic guidelines for Alzheimer's disease. *Alzheimers Dement*. 2011;7(3):270-279. doi:10.1016/j.jalz.2011.03.008
6. McKeith IG, Ferman TJ, Thomas AJ, et al. Research criteria for the diagnosis of prodromal dementia with Lewy bodies. *Neurology*. 2020;94(17):743-755. doi:10.1212/WNL.0000000000009323
7. Litvan I, Goldman JG, Tröster AI, et al. Diagnostic criteria for mild cognitive impairment in Parkinson's disease: *Movement* Disorder Society Task Force guidelines. *Mov Disord*. 2012;27(3):349-356. doi:10.1002/mds.24893
8. Nasreddine ZS, Phillips NA, Bédirian V, et al. The Montreal Cognitive Assessment, MoCA: A Brief Screening Tool For Mild Cognitive Impairment. *J Am Geriatr Soc*. 2005;53(4):695-699. doi:10.1111/j.1532-5415.2005.53221.x
9. Movement Disorder Society Task Force on Rating Scales for Parkinson's Disease. The Unified Parkinson's Disease Rating Scale (UPDRS): Status and recommendations. *Mov Disord*. 2003;18(7):738-750. doi:10.1002/mds.10473
10. Ferman TJ, Smith GE, Boeve BF, et al. DLB fluctuations: Specific features that reliably differentiate DLB from AD and normal aging. *Neurology*. 2004;62(2):181-187. doi:10.1212/WNL.62.2.181
11. Yesavage JA, Brink TL, Rose TL, et al. Development and validation of a geriatric depression screening scale: A preliminary report. *J Psychiatr Res*. 1982;17(1):37-49. doi:10.1016/0022-3956(82)90033-4
12. Stiasny-Kolster K, Mayer G, Schäfer S, Möller JC, Heinzel-Gutenbrunner M, Oertel WH. The REM sleep behavior disorder screening questionnaire—A new diagnostic instrument. *Mov Disord*. 2007;22(16):2386-2393. doi:10.1002/mds.21740
13. Cummings JL, Mega M, Gray K, Rosenberg-Thompson S, Carusi DA, Gornbein J. The Neuropsychiatric Inventory: Comprehensive assessment of psychopathology in dementia. *Neurology*. 1994;44(12):2308-2308. doi:10.1212/WNL.44.12.2308

© 2026 Nencha U et al. *JAMA Network Open*.

14. Johns MW. A New Method for Measuring Daytime Sleepiness: The Epworth Sleepiness Scale. *Sleep*. 1991;14(6):540-545. doi:10.1093/sleep/14.6.540

15. Benedict RHB, Schretlen D, Groninger L, Dobraski M, Shpritz B. Revision of the Brief Visuospatial Memory Test: Studies of normal performance, reliability, and validity. *Psychol Assess*. 1996;8(2):145-153. doi:10.1037/1040-3590.8.2.145

16. Bezdicek O, Libon DJ, Stepankova H, et al. Development, Validity, and Normative Data Study for the 12-Word Philadelphia Verbal Learning Test [czP(r)VLT-12] Among Older and Very Old Czech Adults. *Clin Neuropsychol*. 2014;28(7):1162-1181. doi:10.1080/13854046.2014.952666

17. Wechsler D. Wechsler Adult Intelligence Scale--Third Edition. Published online February 11, 2019. doi:10.1037/t49755-000

18. Nikolai T, Štěpánková H, Michalec J, et al. Tests of Verbal Fluency, Czech Normative Study in Older Patients. *Čes Slov Neurol Neurochir*. 2015;78/111(3):292-299. doi:10.14735/amcsnn2015292

19. Woodard JL, Benedict RHB, Salthouse TA, Toth JP, Zgaljardic DJ, Hancock HE. Normative Data for Equivalent, Parallel Forms of the Judgment ofLine Orientation Test. *J Clin Exp Neuropsychol*. 1998;20(4):457-462. doi:10.1076/jcen.20.4.457.1470

20. Walter U, Školoudík D. Transcranial Sonography (TCS) of Brain Parenchyma in Movement Disorders: Quality Standards, Diagnostic Applications and Novel Technologies. *Ultraschall Med - Eur J Ultrasound*. 2014;35(04):322-331. doi:10.1055/s-0033-1356415

21. Mijajlovic MD, Tsivgoulis G, Sternic N. Transcranial Brain Parenchymal Sonography in Neurodegenerative and Psychiatric Diseases. *J Ultrasound Med*. 2014;33(12):2061-2068. doi:10.7863/ultra.33.12.2061

22. Verdon V, Candal-Zurcher A. Appréciation du degré de gravité en neuropsychologie et estimation de la capacité de travail. 2021.

23. Benedict RHB, Schretlen D, Groninger L, Brandt J. Hopkins Verbal Learning Test – Revised: Normative Data and Analysis of Inter-Form and Test-Retest Reliability. *Clin Neuropsychol*. 1998;12(1):43-55. doi:10.1076/clin.12.1.43.1726

24. Sudarshan NJ, Bowden SC. Common Factor Structure of the Ten Subtest Wechsler Adult Intelligence Scale-Fourth Edition in a Clinical Sample and 15 Subtest Version in the Standardization Sample. *Arch Clin Neuropsychol Off J Natl Acad Neuropsychol*. 2023;38(8):1646-1658. doi:10.1093/arclin/acad035

25. Bowden SC, Saklofske DH, Weiss LG. Augmenting the Core Battery With Supplementary Subtests: Wechsler Adult Intelligence Scale—IV Measurement Invariance Across the United States and Canada. *Assessment*. 2011;18(2):133-140. doi:10.1177/1073191110381717

26. St-Hilaire A, Hudon C, Vallet GT, et al. Normative data for phonemic and semantic verbal fluency test in the adult French–Quebec population and validation study in Alzheimer's disease and depression. *Clin Neuropsychol*. 2016;30(7):1126-1150. doi:10.1080/13854046.2016.1195014

27. Gaudreau AS, Macoir J, Hudon C. Normative data for the Color Trails Test in middle-aged and elderly Quebec-French people. *Appl Neuropsychol Adult*. 2025;32(1):116-124. doi:10.1080/23279095.2022.2156291

28. Tremblay MP, Potvin O, Callahan BL, et al. Normative Data for the Rey-Osterrieth and the Taylor Complex Figure Tests in Quebec-French People. *Arch Clin Neuropsychol*. 2015;30(1):78-87. doi:10.1093/arclin/acu069

© 2026 Nencha U et al. *JAMA Network Open*.

29. Bradshaw JL, Nettleton NC, Nathan G, Wilson L. Head and body space to left and right, front and rear—II. Visuotactual and kinesthetic studies and left-side underestimation. *Neuropsychologia*. 1983;21(5):475-486. doi:10.1016/0028-3932(83)90004-0

30. Buysse DJ, Reynolds CF, Monk TH, Berman SR, Kupfer DJ. The Pittsburgh sleep quality index: A new instrument for psychiatric practice and research. *Psychiatry Res*. 1989;28(2):193-213. doi:10.1016/0165-1781(89)90047-4

31. Pfeffer RI, Kurosaki TT, Harrah CH, Chance JM, Filos S. Measurement of Functional Activities in Older Adults in the Community. *J Gerontol*. 1982;37(3):323-329. doi:10.1093/geronj/37.3.323

32. Nikolai T, Stepankova H, Kopecek M, Sulc Z, Vyhnalek M, Bezdicek O. The Uniform Data Set, Czech Version: Normative Data in Older Adults from an International Perspective. Bondi M, ed. *J Alzheimer's Dis*. 2018;61(3):1233-1240. doi:10.3233/JAD-170595

33. Larouche E, Tremblay MP, Potvin O, et al. Normative Data for the Montreal Cognitive Assessment in Middle-Aged and Elderly Quebec-French People. *Arch Clin Neuropsychol*. 2016;31(7):819-826. doi:10.1093/arclin/acw076

34. Drozdová K, Štěpánková H, Lukavský J, Bezdíček O, Kopeček M. Normative Data for the Rey-Osterrieth Complex Figure Test in Older Czech Adults. *Čes Slov Neurol*. 2015(78):529-549.

35. Havlík F, Mana J, Dušek P, et al. Brief Visuospatial Memory Test-Revised: normative data and clinical utility of learning indices in Parkinson's disease. *J Clin Exp Neuropsychol*. 2020;42(10):1099-1110. doi:10.1080/13803395.2020.1845303

36. Bezdicek O, Motak L, Axelrod BN, et al. Czech Version of the Trail Making Test: Normative Data and Clinical Utility. *Arch Clin Neuropsychol*. 2012;27(8):906-914. doi:10.1093/arclin/acs084

37. Nikolai T, Stepankova H, Kopecek M, Sulc Z, Vyhnalek M, Bezdicek O. The Uniform Data Set, Czech Version: Normative Data in Older Adults from an International Perspective. *J Alzheimer's Dis*. 2018;61(3):1233-1240. doi:10.3233/JAD-170595

38. Bezdíček O, Lukavský J, Preiss M. Functional Activities Questionnaire, Czech Version - a Validation Study. *Ceska Slov Neurol Neurochir*. 2011(74):36-42.

39. Manková D, Dudysová D, Novák J, et al. Reliability and Validity of the Czech Version of the Pittsburgh Sleep Quality Index in Patients with Sleep Disorders and Healthy Controls. Angkurawaranon C, ed. *BioMed Res Int*. 2021;2021(1):5576348. doi:10.1155/2021/5576348

40. Fazekas F, Niederkorn K, Schmidt R, et al. White matter signal abnormalities in normal individuals: correlation with carotid ultrasonography, cerebral blood flow measurements, and cerebrovascular risk factors. *Stroke*. 1988;19(10):1285-1288. doi:10.1161/01.STR.19.10.1285

41. Scheltens P, Leys D, Barkhof F, et al. Atrophy of medial temporal lobes on MRI in "probable" Alzheimer's disease and normal ageing: diagnostic value and neuropsychological correlates. *J Neurol Neurosurg Psychiatry*. 1992;55(10):967-972. doi:10.1136/jnnp.55.10.967

42. Harrington DL, Shen Q, Vincent Filoteo J, et al. Abnormal distraction and load-specific connectivity during working memory in cognitively normal Parkinson's disease. *Hum Brain Mapp*. 2020;41(5):1195-1211. doi:10.1002/hbm.24868

43. Popa T, Russo M, Meunier S. Long-lasting inhibition of cerebellar output. *Brain Stimulat*. 2010;3(3):161-169. doi:10.1016/j.brs.2009.10.001

© 2026 Nencha U et al. *JAMA Network Open*.

44. Koch G, Mori F, Marconi B, et al. Changes in intracortical circuits of the human motor cortex following theta burst stimulation of the lateral cerebellum. *Clin Neurophysiol*. 2008;119(11):2559-2569. doi:10.1016/j.clinph.2008.08.008

45. Kishore A, Popa T, Balachandran A, et al. Cerebellar Sensory Processing Alterations Impact Motor Cortical Plasticity in Parkinson's Disease: Clues from Dyskinetic Patients. *Cereb Cortex*. 2014;24(8):2055-2067. doi:10.1093/cercor/bht058

46. Deng ZD, Lisanby SH, Peterchev AV. Electric field depth–focality tradeoff in transcranial magnetic stimulation: Simulation comparison of 50 coil designs. *Brain Stimulat*. 2013;6(1):1-13. doi:10.1016/j.brs.2012.02.005

47. Sobczak-Edmans M, Lo YC, Hsu YC, et al. Cerebro-Cerebellar Pathways for Verbal Working Memory. *Front Hum Neurosci*. 2019;12:530. doi:10.3389/fnhum.2018.00530

48. Viñas-Guasch N, Ng THB, Heng JG, et al. Cerebellar Transcranial Magnetic Stimulation (TMS) Impairs Visual Working Memory. *The Cerebellum*. 2022;22(3):332-347. doi:10.1007/s12311-022-01396-2

49. Huang YZ, Edwards MJ, Rounis E, Bhatia KP, Rothwell JC. Theta Burst Stimulation of the Human Motor Cortex. *Neuron*. 2005;45(2):201-206. doi:10.1016/j.neuron.2004.12.033

50. Vassiliadis P, Beanato E, Popa T, et al. Non-invasive stimulation of the human striatum disrupts reinforcement learning of motor skills. *Nat Hum Behav*. 2024;8(8):1581-1598. doi:10.1038/s41562-024-01901-z

51. Beanato E, Moon HJ, Windel F, et al. Noninvasive modulation of the hippocampal-entorhinal complex during spatial navigation in humans. *Sci Adv*. 2024;10(44):eado4103. doi:10.1126/sciadv.ado4103

52. Antal A, Alekseichuk I, Bikson M, et al. Low intensity transcranial electric stimulation: Safety, ethical, legal regulatory and application guidelines. *Clin Neurophysiol*. 2017;128(9):1774-1809. doi:10.1016/j.clinph.2017.06.001

53. Grossman N, Bono D, Dedic N, et al. Noninvasive Deep Brain Stimulation via Temporally Interfering Electric Fields. *Cell*. 2017;169(6):1029-1041.e16. doi:10.1016/j.cell.2017.05.024

54. Stoupis D, Samaras T. Non-invasive stimulation with temporal interference: optimization of the electric field deep in the brain with the use of a genetic algorithm. *J Neural Eng*. 2022;19(5):056018. doi:10.1088/1741-2552/ac89b3

55. Fan L, Li H, Zhuo J, et al. The Human Brainnetome Atlas: A New Brain Atlas Based on Connectional Architecture. *Cereb Cortex*. 2016;26(8):3508-3526. doi:10.1093/cercor/bhw157

56. Gajdoš M, Mikl M, Mareček R. Mask_explorer: A tool for exploring brain masks in fMRI group analysis. *Comput Methods Programs Biomed*. 2016;134:155-163. doi:10.1016/j.cmpb.2016.07.015

57. Power JD, Barnes KA, Snyder AZ, Schlaggar BL, Petersen SE. Spurious but systematic correlations in functional connectivity MRI networks arise from subject motion. *NeuroImage*. 2012;59(3):2142-2154. doi:10.1016/j.neuroimage.2011.10.018

58. Calhoun VD, Liu J, Adalı T. A review of group ICA for fMRI data and ICA for joint inference of imaging, genetic, and ERP data. *NeuroImage*. 2009;45(1, Supplement 1):S163-S172. doi:10.1016/j.neuroimage.2008.10.057

59. Ma S, Correa NM, Li XL, Eichele T, Calhoun VD, Adali T. Automatic Identification of Functional Clusters in fMRI Data Using Spatial Dependence. *IEEE Trans Biomed Eng*. 2011;58(12):3406-3417. doi:10.1109/TBME.2011.2167149

© 2026 Nencha U et al. *JAMA Network Open*.

60. Li YO, Adalı T, Calhoun VD. Estimating the number of independent components for functional magnetic resonance imaging data. *Hum Brain Mapp*. 2007;28(11):1251-1266. doi:10.1002/hbm.20359

61. Bates D, Mächler M, Bolker B, Walker S. Fitting Linear Mixed-Effects Models Using **lme4**. *J Stat Softw*. 2015;67(1). doi:10.18637/jss.v067.i01

62. Luke SG. Evaluating significance in linear mixed-effects models in R. *Behav Res Methods*. 2017;49(4):1494-1502. doi:10.3758/s13428-016-0809-y

63. Lenth R. emmeans: Estimated Marginal Means, aka Least-Squares Means. https://github.com/rvlenth/emmeans

64. Ryu E. Effects of skewness and kurtosis on normal-theory based maximum likelihood test statistic in multilevel structural equation modeling. *Behav Res Methods*. 2011;43(4):1066-1074. doi:10.3758/s13428-011-0115-7

65. Ben-Shachar M, Lüdecke D, Makowski D. effectsize: Estimation of Effect Size Indices and Standardized Parameters. *J Open Source Softw*. 2020;5(56):2815. doi:10.21105/joss.02815

66. Cohen J. *Statistical Power Analysis for the Behavioral Sciences*. 2. ed., reprint. Psychology Press; 2009.

67. Bürkner PC. brms: Bayesian Regression Models using "Stan." Published online May 8, 2015:2.22.0. doi:10.32614/CRAN.package.brms

68. Vehtari A, Gelman A, Simpson D, Carpenter B, Bürkner PC. Rank-Normalization, Folding, and Localization: An Improved Rˆ for Assessing Convergence of MCMC (with Discussion). *Bayesian Anal*. 2021;16(2). doi:10.1214/20-BA1221

69. Wagenmakers EJ, Lodewyckx T, Kuriyal H, Grasman R. Bayesian hypothesis testing for psychologists: A tutorial on the Savage–Dickey method. *Cognit Psychol*. 2010;60(3):158-189. doi:10.1016/j.cogpsych.2009.12.001

70. Gabry J, Simpson D, Vehtari A, Betancourt M, Gelman A. Visualization in Bayesian Workflow. *J R Stat Soc Ser A Stat Soc*. 2019;182(2):389-402. doi:10.1111/rssa.12378

71. Maceira-Elvira P, Popa T, Schmid AC, et al. Native learning ability and not age determines the effects of brain stimulation. *Npj Sci Learn*. 2024;9(1):69. doi:10.1038/s41539-024-00278-y

72. Sharko J, Grinstein G, Marx KA. Vectorized Radviz and Its Application to Multiple Cluster Datasets. *IEEE Trans Vis Comput Graph*. 2008;14(6):1444-1427. doi:10.1109/TVCG.2008.173

73. Vassiliadis P, Stiennon E, Windel F, Wessel MJ, Beanato E, Hummel FC. Safety, tolerability and blinding efficiency of non-invasive deep transcranial temporal interference stimulation: first experience from more than 250 sessions. *J Neural Eng*. 2024;21(2):024001. doi:10.1088/1741-2552/ad2d32

74. Piao Y, Ma R, Weng Y, et al. Safety Evaluation of Employing Temporal Interference Transcranial Alternating Current Stimulation in Human Studies. *Brain Sci*. 2022;12(9):1194. doi:10.3390/brainsci12091194

© 2026 Nencha U et al. *JAMA Network Open*.
