# Supplementary material for: Multifocal Noninvasive Deep Brain Stimulation to Enhance Cognition in Mild Cognitive Impairment: A Crossover Trial
Source: JAMA Netw Open. 2026 Jul 6;9(7):e2621756. doi: 10.1001/jamanetworkopen.2026.21756 (PMC13338810; doi:10.1001/jamanetworkopen.2026.21756)
Supplement: Supplement 3. — Data Sharing Statement [file jamanetwopen-e2621756-s003.pdf]

Data Sharing Statement

Nencha. Multifocal Noninvasive Deep Brain Stimulation to Enhance Cognition in Mild Cognitive Impairment. *JAMA Netw Open*. Published July 06, 2026.
doi:10.1001/jamanetworkopen.2026.21756

Data
Additional Information: In this quadruple-blind, randomized, placebo-controlled crossover-design study, we enrolled MCI patients and age-matched healthy controls (HC) from two research sites (EPFL, Switzerland and MUNI, Czech Republic). All studies were conducted in accordance with the Declaration of Helsinki and approved by the Cantonal Ethics Committee of Vaud, Switzerland (SNCTP000003928 | BASEC2020-00127) and ethics committee of Masaryk University (EKV-2020-019). All participants provided written informed consent before the data acquisition. The study is registered at ClinicalTrials.gov (NCT07090681).
Data available: Yes
Data types: Deidentified participant data
How to access data: irena.rektorova@fnusa.cz for MUNI cohort friedhelm.hummel@epfl.ch for EPFL cohort Data available upon request
When available: With publication

Supporting Documents
Document types: None

Additional Information
Who can access the data: researchers whose proposed use of the data has been approved
Types of analyses: any purpose
Mechanisms of data availability: after approval of a proposal with a signed data access agreement
Any additional restrictions: none
